# Lewis Structures from Open Quantum Systems Natural Orbitals: Real Space Adaptive Natural Density Partitioning

**DOI:** 10.1021/acs.jpca.1c01689

**Published:** 2021-04-28

**Authors:** Evelio Francisco, Aurora Costales, María Menéndez-Herrero, Ángel Martín Pendás

**Affiliations:** Departamento de Química Física y Analítica, Facultad de Química, Universidad de Oviedo, 33006 Oviedo, Spain

## Abstract

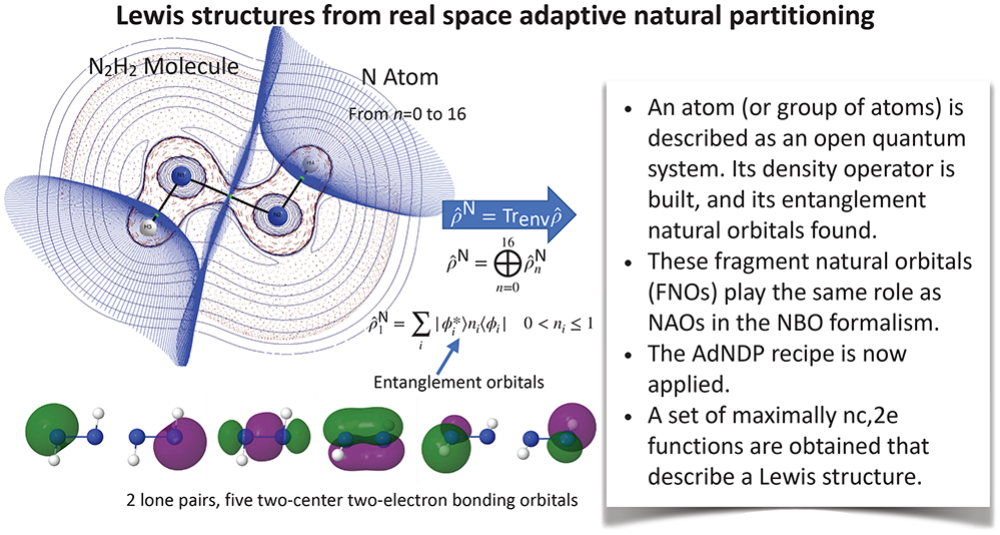

Building chemical models from state-of-the-art electronic structure calculations is not an easy task, since the high-dimensional information contained in the wave function needs to be compressed and read in terms of the accepted chemical language. We have already shown (Phys. Chem. Chem. Phys.2018, 20, 213683009582910.1039/c8cp04090g) how to access Lewis structures from general wave functions in real space by reformulating the adaptive natural density partitioning (AdNDP) method proposed by Zubarev and Boldyrev (Phys. Chem. Chem. Phys.2008, 10, 52071872886210.1039/b804083d). This provides intuitive Lewis descriptions from fully orbital invariant position space descriptors but depends on not immediately accessible higher order cumulant density matrices. By using an open quantum systems (OQS) perspective, we here show that the rigorously defined OQS fragment natural orbitals can be used to build a consistent real space adaptive natural density partitioning based only on spatial information and the system’s one-particle density matrix. We show that this rs-AdNDP approach is a cheap, efficient, and robust technique that immerses electron counting arguments fully in the real space realm.

## Introduction

Few chemical concepts are more venerable than the hundred-year-old two-center, two-electron (2c,2e) bond introduced by Lewis.^1^ Its importance can only be judged appropriately after noticing that, as new knowledge appeared, the model was suitably generalized while maintaining its core safe and sound, as when the debate on the structure and chemistry of boron compounds was settled by Lipscomb^2^ with the introduction of three-center, two-electron links. Today, an extended multicenter framework in which Lewis pairs engage in *n*-center bonds has been shown to provide appropriate descriptions of the electronic structure of a vast number of compounds. Recovering Lewis structures from the output of accurate electronic structure calculations also has implications in modern chemistry and is of paramount importance to connect modern energy decomposition analyses and the theory of chemical bonding.^3^

From the theoretical point of view, however, the recovery of Lewis (or extended-Lewis) pictures from the everyday more accurate calculations at hand has faced several conceptual difficulties, for it is only when minimal basis sets and mean-field descriptions are used that a simple association of doubly occupied one-electron states and Lewis structures becomes possible. When this model realm is abandoned, we are in need of efficient ways (i) to recast the computed wave functions in terms of effective minimal basis sets and (ii) to build effective one-electron states in the case of nonmean-field descriptions to populate the Lewis structure(s).

Both requisites are intimately linked to orbital localization strategies,^4−14^ which rotate a given one-electron basis by means of an arbitrarily chosen maximization criterion, and also to the natural basis concept,^15^ which introduces a set of maximally occupied one-electron states from a general multiconfigurational description by diagonalizing the one-particle density matrix (1RDM). A well developed and mostly popular implementation of this program is the natural bond orbital (NBO) formalism of Weinhold and co-workers,^16−19^ that successively diagonalizes atomic, diatomic or generally *n*-center blocks of the 1RDM, written in a localized basis, to get chemically appealing Lewis pictures. The NBO paradigm has been extremely successful but suffers from the arbitrariness and cumbersome^20^ character of the procedure that builds the starting natural atomic orbital (NAO) basis set. A somewhat similar approach that shares the quasi-minimal basis provided by the NAO algorithm while generalizing the NBO recipe to true multicenter cases was provided by Zubarev and Boldyrev.^21^ In their so-called adaptive natural density partitioning (AdNDP), the 1RDM is written in the NAO basis and its *n*-center blocks are iteratively built from *n* = 1 and diagonalized after being depleted from the contributions that come from the eigenvectors with large (≈2) occupancies that were obtained in the (*n* – 1)-center step of the process. AdNDP has found its way in cluster and solid state chemistry, since it has also been generalized to periodic systems.^22−26^

Both NBO and AdNDP depend crucially on the NAO prescription, i.e., on the construction of a quasi-minimal basis set, and are not invariant under orbital transformations. Leaving orbital in favor of real space provides a potential means to improve this situation, since it has been shown that well-behaved, orbital invariant descriptors of *n*-center bonds can be built from *n*th-order spatially reduced density matrices (nRDMs).^27^ For instance, natural adaptive orbitals^27,28^ (NAdOs) obtained from the cumulant part of the nRDMs recover NBO-like images that include electron correlation explicitly. Since there is usually no free-lunch, the NAO arbitrariness is substituted in these techniques by the choice of the atomic partition. Many of these exist that are usually divided into the so-called fuzzy and exhaustive categories, depending on whether the atomic (or fragment) domains interpenetrate or not. In the former class we can find the Hirshfeld partitioning and its many variants (see ref (29) and references therein), while in the latter we may consider the quantum theory of atoms in molecules (QTAIM),^30^ or any other topological decomposition induced by the gradient of a scalar field. Although choosing one or other partitioning is a matter of taste for some, we think that the conceptual rigor of the QTAIM, which provides atomic kinetic energies better defined than in other partitioning procedures, makes it stand out among all the others, and we have chosen it in the following.

We have shown recently^31^ how to build a hierarchy of real space analogues of the AdNDP scheme, which we call the *real space adaptive natural cumulant partitioning* (rs-AdNCP). In its simplest version, it takes profit of the exact reconstruction of the total 1RDM from the second-order cumulant density, ρ_c_^2^, . Here Ω_a_ is a QTAIM atomic domain. The atomic densities ρ^a^ can be expanded in a given basis set, so this procedure effectively bypasses the NAO prescription in the standard AdNDP. Once the ρ^a^ matrices have been obtained for all atomic domains and diagonalized, their high occupation eigenvectors are selected and stored, and the AdNDP game is started. In the two-center step, the one-particle matrices for all the AB pairs of centers are built and the set of all previously found highly occupied eigenvectors is subtracted from them. These new objects are diagonalized, and their dominant eigenvectors selected as two-center links. The procedure proceeds iteratively until all electrons have been taken into account (up to a threshold). It was shown that the rs-AdNCP image is indistinguishable from the AdNDP one in simple cases.

The use of cumulants has a number of advantages but also some drawbacks. As already stated, the AdNCP (*n*c,2e) links take into account *explicitly* electron correlation, not only through its mean-field effect on the 1RDM. This makes this procedure particularly useful in strong correlation cases, like homolytic bond dissociations. In turn, the need to compute the second order cumulant density makes the strategy computationally intensive and not immediately available from the output of standard electronic structure packages. Thus, a 1RDM-only, still real space alternative to the rs-AdNCP methodology should be wellcome.

A possible solution to this problem is provided here by considering an atom or fragment as an open quantum system (OQS). We have already shown how to define the reduced density operators of a real space subsystem^32^ and have used them to provide a very clear interpretation of atomic local spins.^33^ We expect the OQS perspective to provide interesting insights into the theory of chemical bonding in the near future. One of the simplest results emanating from an OQS viewpoint is a rigorous definition of the 1RDM of a fragment that is independent of the cumulant expansion. Using it, we place ourselves at the end of the first step of any AdNDP-like algorithm: having solved the arbitrary step of obtaining a quasi-minimal description of the atomic one-particle density. After this, the standard AdNDP machinery does the rest of the work.

We will first introduce real space OQSs succinctly. Then the *real space adaptive natural density partitioning* (rs-AdNDP) algorithm will be presented, and finally some simple cases analyzed in detail to demonstrate the overall good performance of the new method. We will end with a short summary and some conclusions.

## Methods

### Brief Survey of Open Quantum Subsystems in Real Space

Any quantum mechanical subsystem is an object coupled to its environment and can be rigorously studied from the open quantum system (OQS) point of view. This discipline is expanding quickly due to its importance in emerging technologies such as quantum control or quantum computing.^34,35^ A more comprehensive account of real space OQSs can be found in ref (32). Here we will only consider a system S described by a pure state from which we extract a subsystem A such that A ∪ A̅ = S, B ≡ A̅.

In this situation, the A subsystem expectation value of an operator *Ô*, ⟨*O*_A_⟩ can be obtained from the so-called reduced density operator of subsystem A, ρ̂^A^, as ⟨*O*_A_⟩ = Tr(*Oρ̂̂*^A^). The reduced operator is obtained by tracing out all the degrees of freedom of A̅ from the full density operator, i.e., integrating out B: ρ̂^A^ = Tr_B_ρ̂. It is important to recognize that although S is in a pure state, A and B are not in general, being instead described by a statistical mixture of pseudopure states characterized, among other things, by different number of particles. In this sense, the average number of electrons in subsystem A, *N*_A_ = *∑*_*i*_*p*^A^(*n*_*i*_) × *n*_*i*_, is obtained in terms of the probabilities that the subsystem be found with an exact integer number of electrons *n*_*i*_.^36−40^

For an *N* electron system, and using *x*, *r* for spin-spatial and spatial-only coordinates, respectively, the full density operator ρ̂ ≡ Ψ*(*x*′) Ψ(*x*), where *x* = *x*_1_, ..., *x*_*N*_. ρ̂_A_ can be obtained^32^ by using *n*-electron spatial projectors , where ω_A_(*x*) is a Heaviside-like weight function defined as
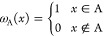
1By noticing that 1 = ω_A_(*x*) + ω_B_(*x*) for each electron, an *N*-electron unit operator  is immediately defined. Applying it to the ρ̂ operator, 2^2*N*^ terms in which primed and unprimed coordinates are separated into A and B regions appear. The trace over B can be recovered if we integrate all coordinates over the B region, leaving only 2^*N*^ nonzero terms.^32^ Each of these contributions contains a given number of α and β electrons in A, a spin sector. If spin is disregarded, we come to a set of groups with common total number of electrons, which are simply called sectors:
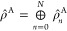
2

In this expression, with ρ̂_0_^A^ = ∫_B_Ψ*(*x*_1_...*x*_*N*_) Ψ(*x*_1_...*x*_*N*_) d*x*_1_...d*x*_*N*_ and, for *n* ≥ 1,
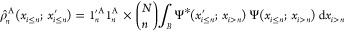
3For each electron sector we can also define reduced density matrices when a given number of its electrons are also integrated out. The reduced density matrix of order *m* ≤ *n* (*m*RDM) of sector *n* is
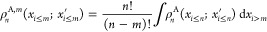
4Spinless versions are immediately written. Taking now eq 3, ρ_*n*_^A,*m*^ can be recast as
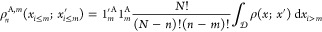
5with  being a domain such that electrons *m* + 1 to *n* are integrated over A, and electrons *n* + 1 to *N* over B. With this, the sum over all sectors provides . This means that if we are not interested in each electron or spin sector, the global subsystem mRDMs can be obtained by simple *m*-particle projection. We now apply this idea to the 1RDM.

### Open Quantum Systems Natural Orbitals

Let us now concentrate on the first-order density matrix of subsystem A. The results of the previous section allow us to write ρ^A,1^(*x*; *x*′) = ω_A_(*x*′) ω_A_(*x*) ρ^1^(*x*; *x*′). Now, if (|*u*_1_⟩···|*u*_*n*_⟩) = |*u*⟩ is an orthonormal basis of molecular spin–orbitals (MSO) in *R*^3^ (for instance, the canonical MSOs of the full system), and we expand ρ^1^(*x*; *x*′) in terms of them we arrive at
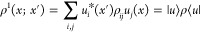
6and 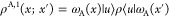
7This simply indicates that the representation matrix of ρ^A,1^(*x*; *x*′) in the basis |*u*⟩ is given by **S**^A^*ρ***S**^A^, where **S**^A^ is the atomic overlap matrix (AOM) of the |*u*_*i*_⟩ MSOs in A, . Notice that ρ^A,1^(*x*; *x*′) only exists when *x*, *x*′ lie in region A.

To obtain the open system fragment natural orbitals (FNOs) of A we must now diagonalize **S**^A^*ρ***S**^A^. Since |*u*⟩ is not orthonormal in A, the following generalized eigenvalue equation must be solved, (**S**^A^*ρ***S**^A^)**C** = **SC** diag(λ), which is equivalent to diagonalizing ρ^A^ = (**S**^A^)^1/2^ρ(**S**^A^)^1/2^, i.e., ρ^A^**U** = **U** diag(λ). We can alternatively arrive at the last equation by expressing the 1RDM in the basis |*u*^*p*^⟩, obtained from |*u*⟩ by means of a Löwdin symmetric orthogonalization procedure, |*u*^*p*^⟩ = |*u*⟩(**S**^A^)^−1/2^. In the |*u*^*p*^⟩ basis, which is orthonormal in A, the matrix representation of ρ^A,1^(*x*; *x*′) is directly ρ^A^. After ρ^A^ is diagonalized, the FNOs of A are given by |φ⟩ = |*u*^*p*^⟩**U** = |*u*⟩(**S**^A^)^−1/2^**U** ≡ |*u*⟩**C**. The φ_*i*_’s form an orthonormal one-electron basis in A, ⟨φ|φ⟩_A_ = *I*, although they are not orthonormal in *R*^3^, , since **C** is not unitary. Again, the values of all these functions are only relevant within region A, although it is customary to show pictorial representations in the full 3D space.

For single-determinant wave functions (SDW), the ρ matrix is the unit matrix, ρ = *I*, so that the matrix of ρ^A^ coincides with **S**^A^. If **U** is the matrix that diagonalizes **S**^A^, the FNOs can be written as |φ⟩ = |*u*⟩**U** diag(λ_*i*_^–1/2^), where the λ_*i*_’s are the eigenvalues of **S**^A^ and . In this case, the φ_*i*_’s are orthogonal, but not normalized, in *R*^3^. As already noticed,^32^ the FNOs in this case are directly Ponec’s domain natural orbitals (DNOs).^41,42^

Starting from the |*u*^*p*^⟩ = |*u*⟩(**S**^A^)^−1/2^ orbitals, and defining |*u̅*^*p*^⟩ = |*u*⟩(**S**^A^)^−1/2^**V**, where **V** is an arbitrary unitary matrix, the set |*u̅*^*p*^⟩ is also orthonormal in A. In this new basis, the matrix representation of ρ^A,1^ becomes ρ̅^A^ = **V**^†^ρ^A^**V**. Choosing **V** = **U** in the case of a SDW makes ρ̅^A^ already diagonal, i.e., ρ̅^A^ = diag(λ_*i*_). In the general multideterminant wave function (MDW) case, it can be shown that the FNOs |φ⟩ and their eigenvalues λ_*i*_ are the same regardless ρ^A^ or ρ̅^A^ being diagonalized. This is related to the Schmidt decomposition of the underlying Hilbert space.

Since both **S**^A^ and ρ are definite positive matrices, their natural occupations λ_*i*_ satisfy 0 ≤ λ_*i*_ ≤ 1 always. For this reason, these FNOs admit a simpler chemical interpretation than those defined by Ponec in the case of MDWs, which can lead to negative occupation numbers. We have also shown^36^ that, in the case of single SDWs, the natural occupations have a statistical interpretation: each λ_*i*_ is equal to the probability of finding an electron described by the FNO φ_*i*_ in region A and 1 – λ_*i*_ in the complementary region A̅. Since  and ⟨φ_*i*_|φ_*i*_⟩_A_ = 1, a FNO with λ_*i*_ close to 1.0 has ⟨φ_*i*_|φ_*i*_⟩_A̅_ ≃ 0; i.e., it is almost entirely localized in A.

It is thus clear that a fully consistent OQS generalization of the concept of 1RDM for a general spatial domain exists (i.e., eq 7). This is our starting point for an AdNDP-like construction that does not depend on further order cumulant density matrices.

### A Real Space Adaptive Natural Density Partitioning Algorithm

The usefulness of FNOs (or that of NAdOs in the cumulant variant) to solve the natural atomic orbital step of the standard NBO and AdNDP recipes lies in their localization properties. Since FNOs of an atomic region A with saturated occupancies (λ_*i*_ ≈ 1 or λ_*i*_ ≈ 2 when spin is traced out in closed-shell cases) are (almost) fully localized in A, they must exactly correspond to either cores or lone pairs (or localized spins in radical cases). No minimal basis construction is thus needed to deplete the OQS 1RDM from them. They are directly built and immediately found. Similarly, if the 1RDM of the AB diatomic pair is depleted from the previously found cores and lone pairs of both A and B, then its occupation saturated FNOs will again be fully localized in the AB region, thus corresponding to two-center links. No extra steps are needed, and the iterative AdNDP recipe can be applied straightforwardly.

The strategy toward an OQS rs-AdNDP algorithm is as follows. The atomic overlap matrices **S**^A^ of all the centers of the system are computed and diagonalized to construct the (**S**^A^)^1/2^ and ρ^A^ matrices. Each ρ^A^ is diagonalized and its eigenvectors φ_*i*_^A^ with occupations (λ_*i*_^A^) close to 1.0 selected (a threshold value must be chosen) and stored. Each eigenvector φ_*i*_^A^ with λ_*i*_^A^ ≃ 1.0 is associated with a core electron (or an electron forming part of a lone pair) of atom A. After all centers have been considered, in a second step, ρ_1_(*x*; *x*′) is depleted from the 1RDM due to the set of all previously found highly occupied eigenvectors (expressed back in the canonical basis), giving
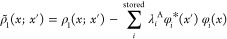
8and the matrices ρ̃^A+B^ defined as ρ̃^A+B^ = (*S*^A^ + *S*^B^)^1/2^ρ̃(*S*^A^ + *S*^B^)^1/2^ are built and diagonalized for all AB pairs, selecting and storing again the eigenvectors with occupations (λ_*i*_^AB^) close to 1.0. They represent electrons involved in two-center (2c) bonds. The procedure is then repeated with trios of atoms ABC, building ρ̃(*x*) as
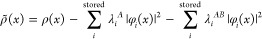
9and diagonalizing ρ̃^A+B+C^ = (**S**^A^ + **S**^B^ + **S**^C^)^1/2^ρ̃(**S**^A^ + **S**^B^ + **S**^C^)^1/2^ for all ABC trios, etc. This iterative process is repeated until the total number of electrons has been exhausted. The final result is a generalized Lewis structure and a partition of ρ(*x*) into orbital contributions which, in turn, are grouped into one-center (1c), (2c), three-center (3c), ... categories. Since each of these functions satisfies , each term of the form λ_*i*_^A^|φ_*i*_(*x*)|^2^, λ_*i*_^AB^|φ_*i*_(*x*)|^2^, ... from eqs 8, 9, ... accounts exactly for the density of a single electron when it is integrated in *R*^3^, so that at the end of the process there are no electrons left. Although the functions are only true FNOs in the one-center step, we will call them generalized FNOs or simply FNOs in the following.

Actually, eqs 8 and 9 can also be written in an equivalent form by eliminating from them the eigenvalues λ_*i*_ and replacing each φ_*i*_ by the normalized in *R*^3^ MSO φ̃_*i*_ = λ_*i*_^1/2^φ_*i*_. A molecule with only core electrons and lone pairs is revealed by the fact that ρ̃(*x*) of eq 8 is zero. If the molecule also has two-center two-electrons (2c,2e) bonds, ρ̃(*x*) of eq 9 is zero, etc.

In closed-shell molecules, the above procedure is carried out in a somewhat different way. Instead of working with ρ(*x*; *x*′), we handle its purely euclidean analogue ρ(*r*; *r*′), obtained from ρ(*x*; *x*′) after integrating the spin variables. This means that the eigenvectors resulting from the diagonalization of the initial ρ^A^ which are selected and stored are those with λ_*i*_^A^ ≃ 2.0, which correspond to core or lone-pair molecular orbitals (MO), the eigenvectors stored and saved in the second step have λ_*i*_^AB^ ≃ 2.0 and represent the prototypical (2c,2e) bonds, eigenvectors with λ_*i*_^ABC^ ≃ 2.0 in the third step are (3c,2e) bonds, and so on.

Considerable amounts of time can be saved in the procedure by following the same prescriptions that were already mentioned in our first rs-AdNCP implementation.^31^ Usually, hydrogen atoms can be safely skipped in the first one-center diagonalizations, since there are hardly any hydrogens in molecular environments with an electronic charge that is almost exactly 2.0. Two-center diagonalizations can be limited to AB pairs with A and B separated by no more than, e.g., *nb* ∼ 2–4 links, which are determined by pure geometrical recipes from the atomic radii of the involved atoms, and the search for trios, quartets, ..., n-tuples of atoms similarly limited to cases where the selected group is connected; i.e., each atom of the n-tuple is geometrically linked with at least another atom of the group.

The above strategies to save computer time do not prevent the procedure from being quasi-automatic, and the user simply needs to decide whether the hydrogen atoms are skipped or not, to provide a value for *nb*, and to ascertain whether two atoms are geometrically linked or not. A manual mode is also available in which the atoms, pairs, and general atomic n-tuples to be searched for are directly established by the user.

To end this section, we want to note that the present formalism can be applied not only with exhaustive partitions of *R*^3^ but also with fuzzy decompositions such as the Hirshfeld-like ones, based on information theory grounds. If subsystem A is made up of a single atom, ω_A_(*x*) changes smoothly with *x*, approaching 1.0 when *x* is close to the nucleus of the atom and vanishing as one moves away from it. All the equations of the last three subsections remain valid, but the computation of the atomic overlap matrices **S**^A^, defined as , requires now three-dimensional integrations extended to *R*^3^.

### Implementation of the rs-AdNDP Algorithm

**Figure 1:**
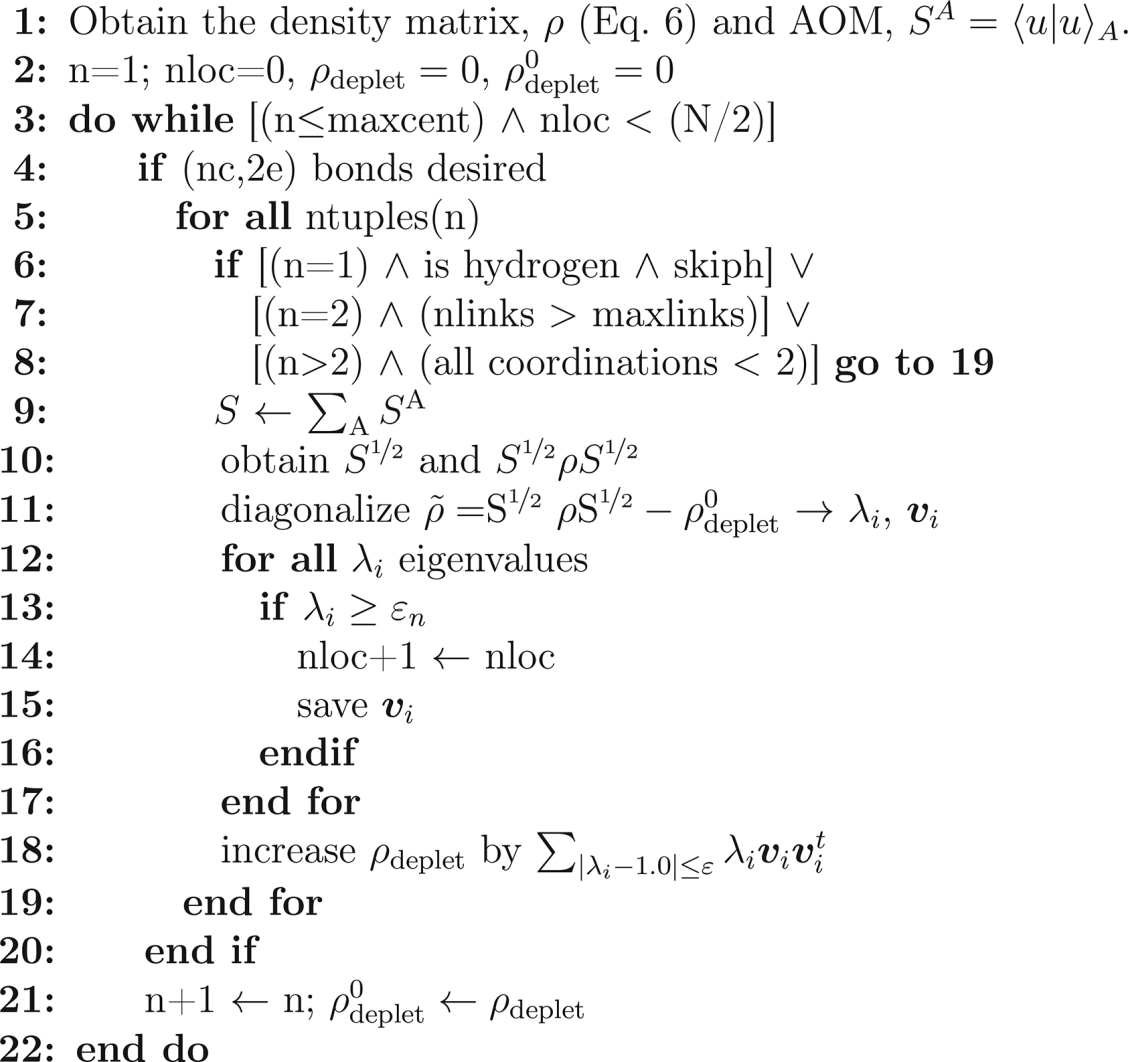
Simplified pseudocode flowchart of the present rs-AdNDP algorithm.

The method just outlined has been implemented as shown in the flowchart diagram of Figure 1. For the sake of simplicity, the chart corresponds to a closed shell system, although the changes that would have to be included either for open-shell systems or for unrestricted descriptions are minimal. In these cases, the flowchart should simply be run twice, once for each spin projection (α or β).

The starting point of the process (line 1) is computing ρ, the density matrix representation of ρ_1_(*x*; *x*′) on the basis of canonical MOs (or ρ^σ^ with σ = α, β in the case of open-shell or unrestricted wave functions) and the AOM between these orbitals for every atomic domain, **S**^A^ = ⟨*u*|*u*⟩_A_. In step 2, we set the value of n, the starting number of centers for which the algorithm will attempt a search of localized FNOs. Usually, we will choose n=1 and the localized FNOs found in the first iteration will correspond to cores or lone pairs. The number of localized FNOs is also initialized in step 1 (nloc=0), as well as ρ_deplet_ and ρ_deplet_^0^, the matrix representation of the already found localized FNOs in the canonical basis, and ε_*n*_, an occupation threshold that separates nonlocalized from localized FNOs according to the value of the eigenvalues of ρ̃ (step 11). Any eigenvalue satisfying λ_*i*_ ≥ ε_*n*_ is assumed to be associated with an FNO localized in the current n-tuple region that is being explored.

The loop starting on line 3 runs over all the desired values of n, the number of centers for which searches will be tried. The maximum value of n (maxcent) has to be defined in advance, and specific values of n can be skipped if desired (see line 4). This loop ends when nloc=N/2, where N is the total number of electrons, or when nloc=N^σ^ in the case of open-shell or UHF wave functions. Given a value of n, the **for all** loop in line 5 runs over all possible n-tuples of atoms that can be formed with ncent centers (ncent for n=1,(ncent×(ncent-1))/2 for n = 2, ...), with ncent being the number of atoms of the molecule. Very important to save computer time is line 6: A 1-tuple is skipped if it corresponds to a hydrogen atom, and the choice skiph=.true. has been selected in the input. 2-tuples are also skipped if the number of links necessary to go from the first atom A to the second atom B of the pair (nlinks) is greater than a maximum predefined value (maxlinks). Finally, n-tuples with n≥3 are not considered if the coordination of all the atoms on the n-tuple are smaller than 2. This circumstance necessarily implies that at least one atom of the n-tuple is disconnected from the others.

It is interesting to explain, even briefly, how the coordination of an atom is determined in the method through the so-called minimum length path between atoms *i* and *j* (i.e., the minimum number of links that are needed to reach atom *j* from atom *i*, or vice versa), ω_*ij*_^min^. First, the (ncent×ncent) symmetric matrix **d** with all the interatomic distances *d*_*ij*_ is obtained, taking *d*_*ii*=0_. Second, two atoms *i* and *j*, with covalent radii *r*_*i*_ and *r*_*j*_, are considered to be *geometrically* linked if **d**_*ij*_ ≤ (*r*_*i*_ + *r*_*j*_) × *f*, where *f* is a numerical factor that is chosen greater than 1.0. If this happens, we set **L**_*ij*_ = 1, where **L** is the adjacency matrix. Otherwise, we set **L**_*ij*_ = 0. Too large values of *f* increase the probability that **L**_*ij*_ = 1, and hence the number of pairs that have to be explored in the search of localized FNOs. The coordination of atom *i* is simply the number of ones in the row or column of matrix **L** associated with this atom, and ω_*ij*_^min^ is given by ω_*ij*_^min^ = min{ν|(**L**^ν^)_*ij*_ ≠ 0}, where **L**^ν^ is the νth power of **L**. If (**L**^ν^)_*ij*_ = 0 for all ν ≤ ncent, the system under consideration is formed by at least two nonconnected or *isolated* molecules.

The following step in the procedure (line 9) is computing **S** = *∑*_A_**S**^A^, where the summation runs over all the atoms A of the current n-tuple. In the following step (line 10) we compute **S**^1/2^ and **S**^1/2^*ρ***S**^1/2^. The latter, depleted from ρ_deplet_^0^, is diagonalized in step 11, obtaining its eigenvalues λ_*i*_ and eigenvectors ***v***_*i*_. In lines 12–17, all λ_*i*_’s close enough to one (λ_*i*_ ≥ ε_*n*_) and their associated ***v***_*i*_’s are stored, and nloc is increased accordingly. In the following step (line 18) ρ_deplet_ is increased by the density due to the just found ***v***_*i*_’s with λ_*i*_ ≃ 1.0, expressed back in the canonical basis of MOs. Finally, ρ_deplet_^0^ is updated with the most recent ρ_deplet_, and n increased by 1 in line 21. When *n* = 2, the loop starting at the line 3 will locate the prototypical (2c,2e) bonding MOs, when *n* = 3 the algorithm will attempt to find (3c,2e) MOs, etc.

After successfully feeding the flowchart of Figure 1, the 1RDM will have been distributed into one-center, two-center, etc., contributions, associated respectively to FNOs mainly localized over atomic n-tuples. In a similar way, the transformation from the original molecular orbitals to the final localized ones is given by |φ⟩ = |*u*⟩**C**, where the first columns of **C** represent MOs localized in a single atom (*n* = 1), the following ones are MOs localized in two atoms, and so on.

We have also found relevant to explore how to obtain a set of orthonormal orbitals (in *R*^3^) |φ^ortho^⟩ as similar as possible to the |φ⟩ set. These can be obtained by maximizing each diagonal element  with the condition . As it is well-known, this is achieved through the transformation |φ^ortho^⟩ = |φ⟩**S**^–1/2^, where . Writing |φ^ortho^⟩ as |φ^ortho^⟩ = |*u*⟩*C*^ortho^, a measure of the similarity between the nonorthonormal and orthonormal MOs after this orthonormalization process is given by the matrix ⟨φ|φ^ortho^⟩ = **C**^†^**C**^ortho^. The more similar both types of orbitals are, the more similar to the identity matrix **C**^†^**C**^ortho^ will be as in Figure 1.

One almost final consideration is also due regarding a quantity that will be used when analyzing and discussing the results of the present algorithmic implementation. Consider an orbital φ that is normalized in *R*^3^. The effective number of centers expanded by φ, a measure of how many atomic fragments the function delocalizes over, can be measured by the quantity *n*_eff_(φ)=1/∑_A_⟨φ|φ⟩_A_^2^. When φ is fully localized in A, ⟨φ|φ⟩_A_ ≃ 1, so that ⟨φ|φ⟩_B≠A_ ≃ 0 and *n*_eff_(φ) ≃ 1. On the contrary, if it is equally localized in only two centers A and B, ⟨φ|φ⟩_A_ ≃ ⟨φ|φ⟩_B_ ≃ 1/2, and *n*_eff_(φ) ≃ 2. Finally, if φ is equally delocalized in *n* centers A_1_, ..., A_*n*_, we will have ⟨φ|φ⟩_*Ai*_ = 1/*n* and *n*_eff_(φ) = *n*.

Finally, it is important to make one last comment regarding the choice of the ε_*n*_ thresholds. The rs-AdNDP strategy requires that these quantities be supplied to the program in order for it to classify the FNOs as 1c, 2c, 3c MOs, etc. A ε_*n*_ less than but extremely close to 1.0, will result in the method being unable to find *n*-center FNOs (except maybe for the (1c,2e) 1s atomic core orbitals), and a too low ε_*n*_ will cause almost any MO to fit into the category of (*n*c,2e) FNOs. In short, it seems that the choice in the algorithm of the ε_*n*_’s is a delicate matter and, in a sense, it is. On the other hand, this inconvenience is not exclusive to the present rs-AdNDP method but is also inherent to the NBO, AdNDF, and rs-AdNCP strategies. After completion, the method can give rise, in conflicting cases, to different classifications of the total set of MOs depending of the choice of these ε_*n*_. However, it is important to note that the relevant properties of the FNOs (degree of localization in the different atoms of the system, λ_*i*_ values, their appearance when they are graphically represented, etc.) do not depend on the choice of the ε’s, as long as the latter are not chosen in an arbitrary way. This means that, regardless of the (automatic) classification given by the algorithm of the FNOs into the different categories, a critical analysis of the aforementioned properties must always be carried out in order to know the true character and nature of each of them.

## Results and Discussion

We will now discuss how the implementation of the rs-AdNDP algorithm performs in a number of examples. We have used wave functions obtained at different levels of theory and using different codes, but our domestic program promolden(43) was systematically employed to compute the atomic overlap matrices (AOM) that are necessary. To increase the accuracy of these AOMs, β-spheres were always employed in their calculation, using restricted angular Lebedev quadratures with a variable number of points, depending on the molecule, inside and outside the β-spheres, and Gauss–Chebyshev mapped radial grids (also with a different number of points in each case). In general, we have found that each (*i*, *j*) element of the computed matrix **S**_tot_, defined as **S**_tot_ = *∑*_A_**S**^A^, differs from its exact value (δ_*ij*_) by less than 0.001–0.002. With the AOM and wave functions available, the rs-AdNDP analyses were performed with the in-house edf program.^44^

We will comment on a set of simple closed-shell molecules that exemplify several bonding situations, a couple of prototypical reactions, a 3d-transition metal complex that allows us to relate our method to the unambiguous assignment of oxidation states, and the tetrahedral PtO_4_^2+^ cation, which was controversial a few years ago and that was also examined in our first work on the subject.^31^

### Simple Examples

#### CH_4_

We start with a basic system, the CH_4_ molecule computed at the RHF//cc-pVTZ level, which is well described by a single Lewis structure. Recall that the (*n*c,2e) functions provided by the rs-AdNDP procedure are not truly FNOs beyond *n* = 1, but that we will use a relaxed language and call all of them generalized FNOs. Thus, in methane we can form five FNOs from its five canonical MOs (see the Supporting Information). Since ρ = *I* in this case, diagonalizing the density matrix of the carbon atom, ρ^textC^ = (**S**^C^)^1/2^ρ(**S**^C^)^1/2^ is equivalent to diagonalizing its AOM, so that the (1c,2e) FNOs are directly equivalent to Ponec’s domain natural orbitals.^41,42^

Choosing a conventional threshold parameter ε_1_ = 0.95, a single 1-center FNO with λ = 0.99998, fully localized in C, is obtained that corresponds to the carbon 1s core. After depleting ρ from the density due to this MO (see eq 8), diagonalizing  (*i* = 1–4), and setting ε_1_ = 0.95, we obtain four equivalent 2-center FNOs, with λ = 0.97302, which are associated with the classical σ C–H bonds. Each of them is only slightly more localized over the C atom (49.3%) than over the H_*i*_ one (48.0%).

The four σ C–H MOs have *n*_eff_(φ_*i*_) = 2.109, which means that each of them is barely delocalized over the remaining three hydrogen atoms; i.e., each function is almost a pure (2c,2e) orbital.

#### SO_4_^2–^

The sulfate anion provides another interesting example where basic chemistry ideas can be put to the test. This time we will use DFT to show that the procedure works equally well. We stress that we are here approximating the one-particle density through the pseudo Kohn–Sham Fock–Dirac 1RDM, since there is no well-defined first-order density matrix in Kohn–Sham DFT. This is quite a standard practice in chemical bonding analysis.

We have optimized the *T*_*d*_ structure of the sulfate anion at the B3LYP//def2-QZVPPD level using the GAMESS-US code.^45^ The λ_*i*_ eigenvalues and their corresponding degrees of localization are collected in Table 1. We report results obtained with ε_*i*_ = 0.95, but the image is independent of this value.

**Table 1:** rs-AdNDP Picture for the SO_4_^2–^ Anion Described at the B3LYP//def2-QZVPPD Level^a^

S	%loc (S)		O	%loc (O)
λ_1_ = 1.00000	100.0		λ_6_ = 1.00000	100.0
λ_2_ = 0.99844	99.8		λ_7_ = 0.99758	99.8
λ_3_ = 0.99770	99.8			
λ_4_ = 0.99770	99.8			
λ_5_ = 0.99770	99.8			

S–O pair	%loc (S)	%loc (O)
λ_8_ = 0.97088	18.9(S)	78.1(O)
λ_9_ = 0.96895	4.5(S)	92.4(O)
λ_10_ = 0.96895	4.5(S)	92.4(O)

a The ε threshold was chosen equal to 0.95 in. The degree of localization of each (*n*c,2e) function over its *n* centers is shown in parentheses.

**Figure 2:**
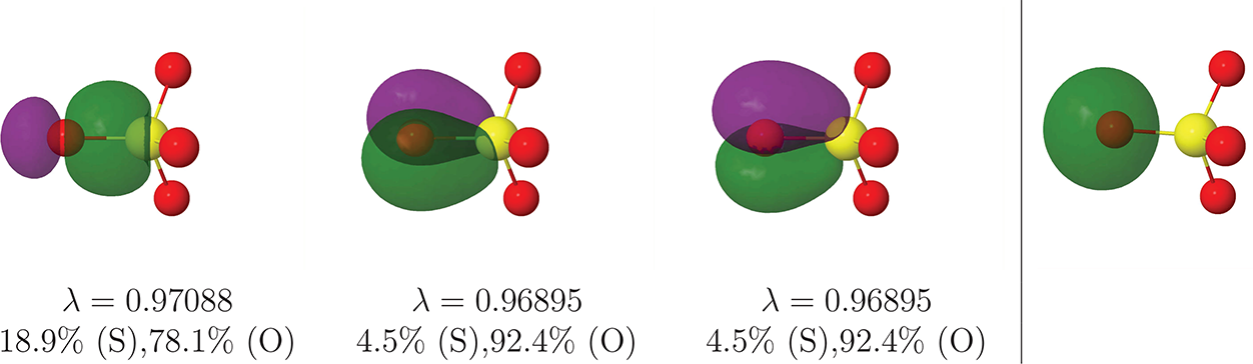
|φ| = 0.05 au isosurface of the σ (left) and π (center and right) S–O FNOs of the SO_4_^2–^ anion at the B3LYP//def2-QZVPPD level of calculation. The rightmost graph corresponds to the lone pair of the oxygen atom.

Five and two (1c,2e) FNOs are found for the sulfur and oxygen atoms, respectively. All of them are fully localized in their atomic basins. Neglecting the core–shells, the only relevant contribution is ϕ_7_, a σ-like oxygen lone pair with a rather large 2s contribution, as evidenced in Figure 2. The rest of the electron pairs are located in the two-center step. Each S–O pair hosts a very clear σ (2c,2e) link, and two rotationally equivalent π contributions that are consistent with the *C*_3*v*_ symmetry of the S–O bonds. These three pairs are well localized in each two-center fragment, spreading less than 3.5% over other centers. A closer look, however, discloses that the π contributions are very localized on the O atoms and delocalize as much over the sulfur as over the rest of the system. It is thus a matter of viewpoint whether to consider them as lone pairs centered at the oxygens or true (2c,2e) links. Even the σ bond is quite polarized, with a barely 20% S contribution.

The generalized FNOs introduced here can be used in the formalism derived by Salvador and co-workers^46^ to unambiguously assign the so-called effective oxidation states (EOS). Very briefly, the ionic approximation is used for each electron pair, which is entirely assigned to the center on which the pair is preferentially localized (in our case, the %loc descriptors). This obviously leads to +6 and −2 EOS for the sulfur and oxygen atoms, respectively. Notice that given the OQS nature of our prescription, these assignments are also compatible with sector probabilities, or with our previously defined electron distribution functions (EDFs).^36−40^ We are working on a general electron counting framework that we will present elsewhere.

**Figure 3:**
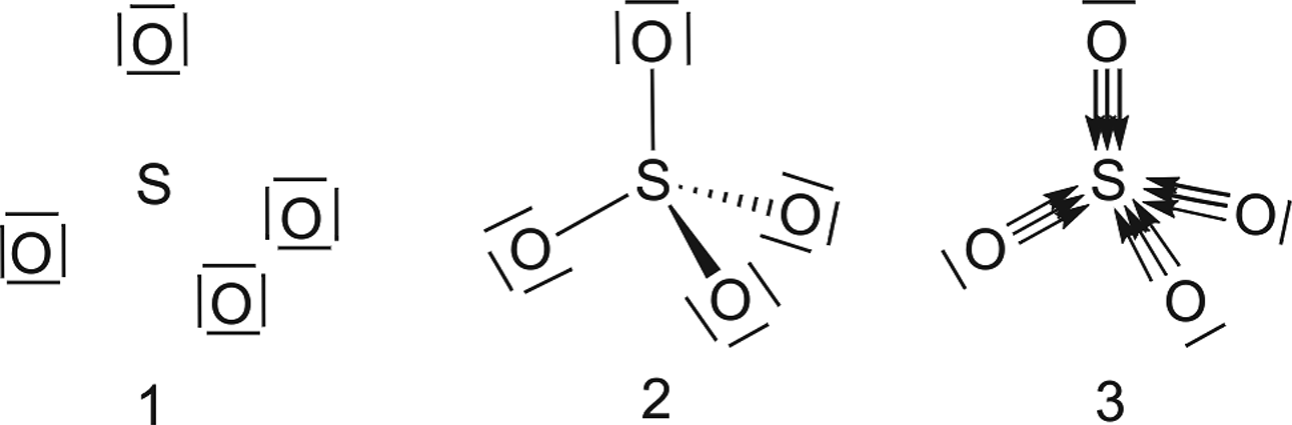
Evolution of the Lewis structures compatible with the rs-AdNDP partition as we loosen or tighten the ionic approximation criterion.

The rs-AdNDP picture provides a solid bridge between quantum mechanical calculations, electron counting techniques, and Lewis structures. If, in line with the EOS ionic approximation, all ϕ_8_, ϕ_9_, ϕ_10_ functions are taken as oxygen lone pairs (thus neglecting the S 20% share in ϕ_8_), an ionic image of SO_4_^2–^ emerges, which justifies the large atomic QTAIM charges of the system and the positive Laplacian of the electron density at the S–O bond critical points, ∇^2^ρ(**r**_bcp_) = 0.249 au, and the large QTAIM charge of the S atom, equal to +3.94 |*e*|. Two other possibilities arise as we loosen the ionic criterion: if the ϕ_8_ contribution is understood as a (very) polar standard (2c,2e) link, then an octet-preserving Lewis structure with +2 and −1 formal charges for the S and O atoms, respectively, appears. Finally, if all the σ, π are taken into account as two-center bonds, fully or partially dative structures that elude any octet expansion are possible, as shown in Figure 3. Overall, what these results show is how different sensitivities when assigning electrons to centers impact the final Lewis image. All valence electrons but the O σ lone pairs are involved in S–O bonding to some extent, and a transition from an ionic to a covalent picture arises when we loosen the criteria to consider electrons fully or partially localized.

No octet expansion is needed. For instance, an analysis of the occupations of the sulfur atomic natural orbitals shows that only 0.3 electrons come from d-like contributions. We will see that this is not necessarily the case.

#### N_2_H_2_

**Figure 4:**
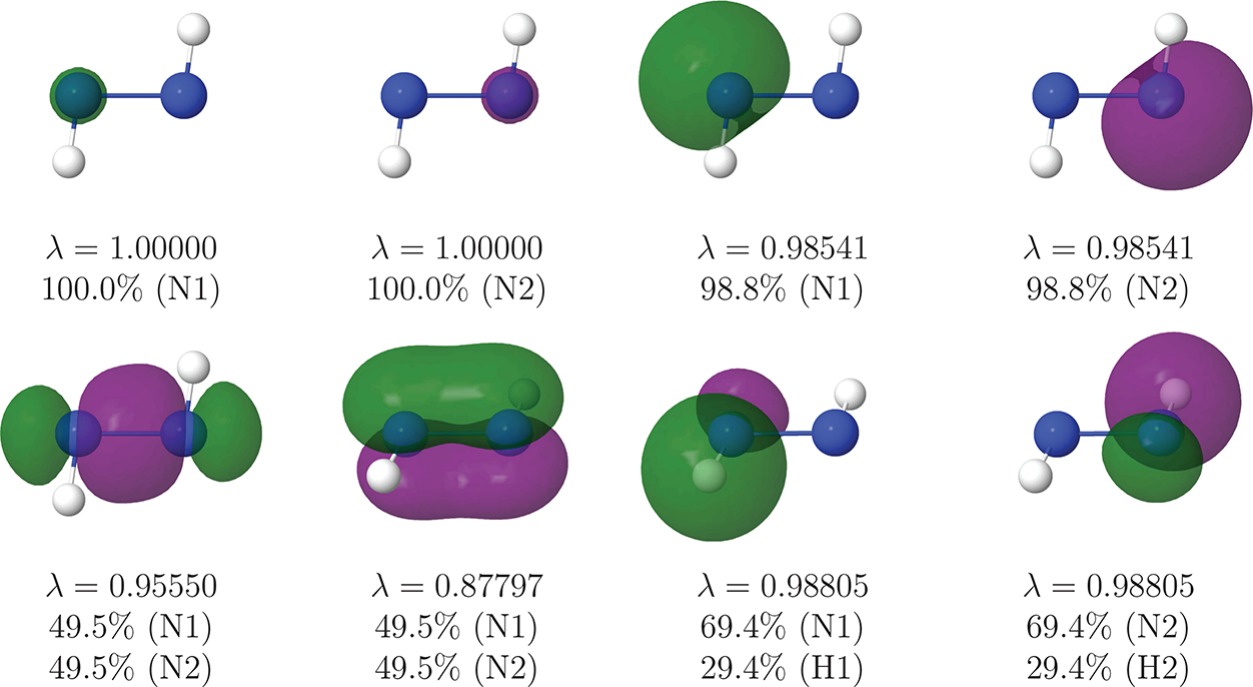
|φ| = 0.05 au isosurface of the eight highest λ_*i*_ FNOs of the N_2_H_2_ molecule at the CAS[12,8]//6-311G(d) level of calculation.

A potentially controversial bonding situation is found N_2_H_2_. To show that the rs-AdNDP procedure is equally powerful at all levels of calculation, we have examined this molecule at the CAS[12,8]//6-311G(d) level. The generalized FNOs are shown in Figure 4.

The image provided is again robust with respect to thresholds, and unique. Besides core and N–H pairs, each N atom hosts a very localized lone sp^2^-like lone pair, and the N–N link is made up of two symmetric σ and π bonds. This picture is also that found with the electron localization function (ELF).^47^ Notice that the N–H bonds are polarized, with a 70/30 share in the N/H atoms, respectively. The Lewis structure describing the system is thus H–N=N–H.

### Chemical Reactions

The rs-AdNDP can also be useful when following changes in the chemical bond along a chemical process. We have considered the canonical *cis*-butadiene plus ethylene Diels–Alder (DA) cycloaddition and the symmetric F^–^+CH_3_F → FCH_3_ + F^–^ S_N_2 substitution.

### *cis*-Butadiene plus Ethylene Diels–Alder (DA) Reaction

We have chosen this reaction as a prototype of simultaneous bond breaking and bond forming process. First, we located the transition state (TS) at the aug-cc-pVDZ^48^/B3LYP^49,50^ level with the GAMESS-US code,^45^ ensuring the existence of a single imaginary frequency. Then, wave functions were derived at 15 points along the intrinsic reaction coordinate (IRC) path^51^ with the def2-QZVPPD^52^ basis set, using the density fitting technique and the corresponding auxiliary def2-QZVPP-jkFIT^53^ basis set, all with the B3LYP functional and a standard Becke grid.^54^

**Figure 5:**
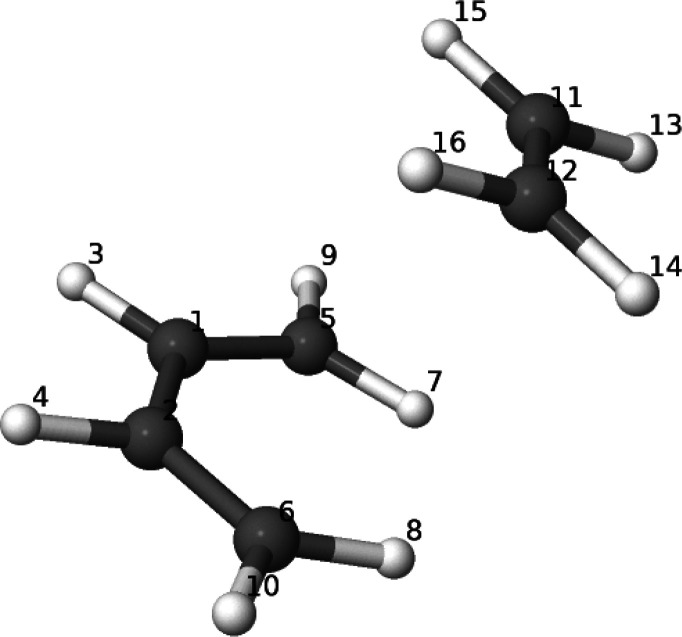
Numbering scheme of the atoms of the *cis*-butadiene plus ethylene system.

All these wave functions were generated with the PySCF code.^55^ The rs-AdNDP image was obtained through our promolden(43) and edf(44) codes, as described above, used to compute the AOM integrals and to get the generalized FNOs, respectively. Atom numbers to be used in the following appear in Figure 5. The IRC has been projected onto the C–C distance (*R*) of any of the two single σ bonds (C_5_–C_11_ or C_6_–C_12_) that are formed during the cycloaddition. The TS is located at *R* = 2.26 Å.

**Figure 6:**
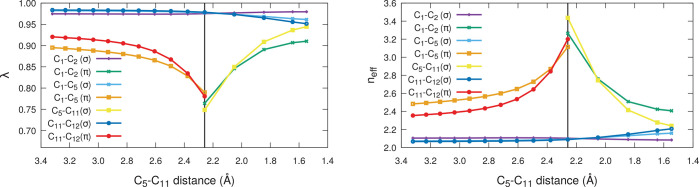
Eigenvalues (λ, left) and effective number of atoms expanded by all the C–C FNOs (*n*_eff_(φ), right) for the butadiene + ethylene DA reaction at the B3LYP//def2-QZVPPD level along the IRC defined in the text. The vertical line signals the transition state (TS).

The evolution with *R* of the λ eigenvalues and the effective number of centers (*n*_eff_) expanded by the FNOs associated with C–C bonds is shown in Figure 6. As expected, the σ skeleton of the butadiene and ethene moieties remains mostly unaltered during the process, as evidenced by the λ eigenvalues and *n*_eff_ values of the σ C_1_–C_2_, C_1_–C_5_, C_2_–C_6_ (equivalent to C_1_–C_5_, not shown in the figure), and C_11_–C_12_ bonds, which change only marginally throughout the reactive process.

On the contrary, the figure shows that the C_1_–C_5_(π), C_2_–C_6_(π), and C_11_–C_12_(π) functions, up to the TS, and the C_1_–C_2_(π), C_5_–C_11_(σ), and C_6_–C_12_(σ), after it, suffer considerable changes, showing a cusp-like behavior close to the transition state. We have found that the first set of solutions get more and more delocalized as we approach the TS, becoming more and more sensitive to the ε threshold. The contrary occurs to the second set. This behavior is compatible with the standard interpretation where an aromatic TS is postulated. As it is well-known, NBO cannot provide a unique Lewis structure for, e.g., benzene, and the two Kekulé resonance structures are found randomly depending on the starting point. A similar behavior is shown by AdNDP (which is inherited by rs-AdNDP). If 6-center links are searched for after the σ skeleton is depleted, then the canonical π orbitals of benzene are obtained. Otherwise, oscillations between the two Kekulé possibilities are found. In the present case, the cusp clearly indicates the inadequacy of a single (2c,2e) description for the system as the TS is approached.

Be that as it may, the DA example shows how bond breaking and bond forming processes can be followed via rs-AdNDP, and also how the procedure includes simple indicators that unveil regions where the single Lewis structure character of a wave function becomes compromised.

**Figure 7:**
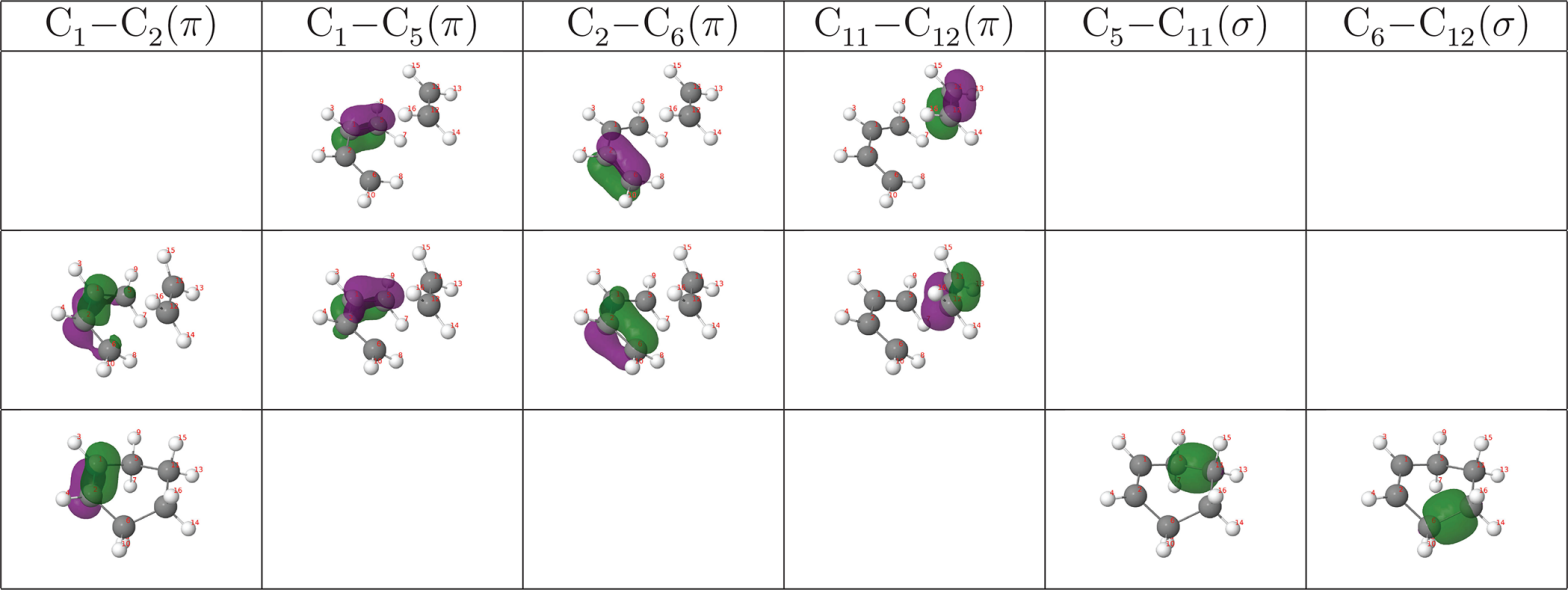
|φ| = 0.1 au isosurface of the six quickly evolving σ and π functions of the butadiene + ethylene DA reaction, computed at the B3LYP//def2-QZVPPD level. Upper, middle, and lower rows correspond to the starting, (close to) TS, and ending points along the reaction path, respectively.

The evolution of the six (2c,2e) functions that evolve along the IRC can be found in Figure 7. Only three of them are populated at any point of the IRC. The increasing delocalization of the butadiene π functions in the TS region stands out.

### F^–^ + CH_3_F → FCH_3_ + F^–^ Reaction

**Figure 8:**
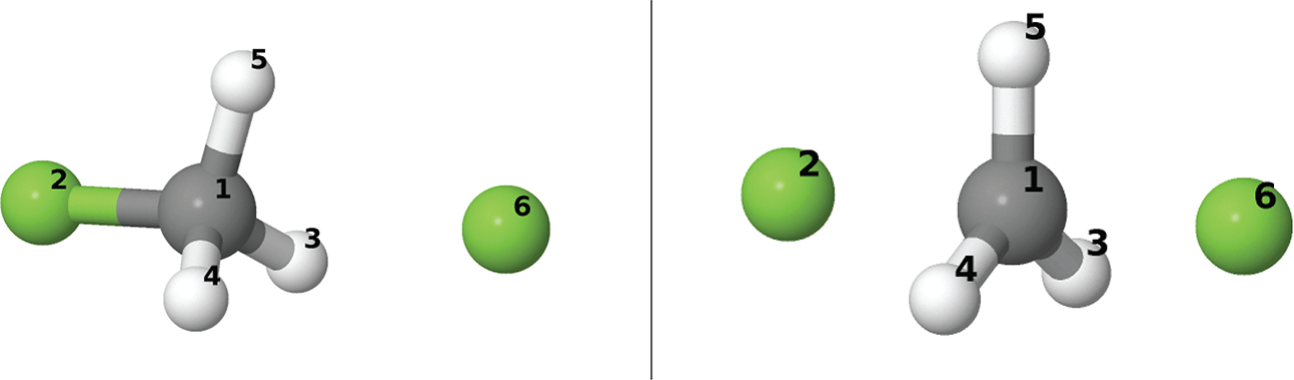
Atomic labels along the fluoride exchange reaction considered in the text. The initial molecular complex as well as the TS geometries are found in the left and right panels, respectively.

We now briefly study the S_N_2 fluoride exchange in fluoromethane, computed at the B3LYP/aug-cc-pVDZ level using the Gaussian09^56^ suite. The TS was located via the QST3 algorithm, and only the forward IRC was examined. Atomic labels are provided in Figure 8.

**Figure 9:**
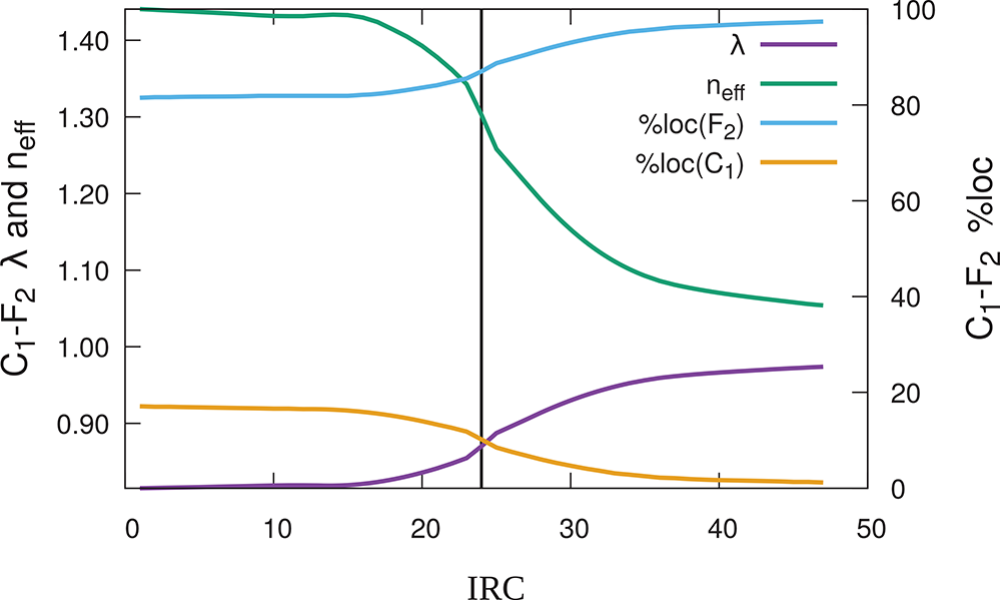
λ and *n*_eff_ (left scale) and degree of localization in C_1_ and F_2_ (right scale) of the (predominantly) C_1_–F_2_ FNO. The vertical line signals the transition state (TS) of the reaction. The ε_*i*_ value was selected equal 0.70.

We have performed the rs-AdNDP analysis using several choices of the ϵ thresholds. The picture is extremely simple and stable, but due to the compact character of the F atom, the classification of the C–F links as one- or two-center bonds depends on the threshold. For instance, when ε_*i*_ = 0.70, only three (2c,2e) C–H σ bonds are found throughout the full IRC, localized about 50–52% in the C atom, 44–45% in one of the H atoms, and negligibly over the rest. The rest are classified as single-center contributions. Besides the expected core and e-symmetry fluorine lone pairs, fully localized along the IRC, the two remaining a-symmetry functions that are classified also as lone pairs suffer a clear evolution in their degree of localization, as shown for one of the fluorine atoms in Figure 9.

It is obvious that it is the very polar nature of the C–F bond that precludes classifying it as a (2c,2e) bond. At the starting point of the IRC, this function is only 17.1/81.1% delocalized over the C/F atoms, but this parameter evolves as it is expected for a bond that breaks, with an inflection point at about the TS. Notice less than 3% spreads over the rest of the system. This can thus be safely considered a very polar (2c,2e) bond, which obviously evolves toward a pure 2p orbital.

The traditional picture is easily recovered if we select a more standard threshold, for instance ε = 0.9. If this is done, the function above is now classified as a (2c,2e) link at the beginning, and a lone pair at the end, and the TS is a normal pentacoordinated one. Although the classification scheme changes with the threshold, the functions do not, remaining basically unaltered over a wide range of ε values.

#### FeF_6_^3–^

We have optimized the geometry and determined the wave function of the FeF_6_^3–^ complex at the unrestricted DFT M06-2X//aug-cc-PVDZ level, both for the *O*_*h*_ high spin (t_2g_^3^e_g_^2^-^6^A_1g_) and the *D*_4*h*_ low spin (e_g_^4^b_2g_^1^-^2^B_2g_) multielectron states. We think that this provides a simple example of how the technique may help in the assignment of electron configurations or effective oxidation states in transition metal chemistry.

The six *O*_*h*_ Fe–F distances (2.0026 Å) are slightly greater than those in the *D*_4*h*_ complex, 1.90608 and 1.96971 Å for equatorial and axial F atoms, respectively. Therefore, there is no difficulty in carrying out a direct comparison of the rs-AdNDP results obtained in both cases. The effective number of centers (*n*_eff_), λ eigenvalues, and % of localization of the FNOs for both complexes are gathered in Tables 2–5. These results were obtained with ε_*i*_ = 0.85. Again, the large difference in electronegativity leads to a description with only one-center contributions. As in our previous example, the two-center nature of the Fe–F links is uncovered if the threshold is increased to 0.90. All our considerations in the last subsection apply here untouched.

**Table 2:** Effective Number of Centers (*n*_eff_), λ Eigenvalues, Percent Localization, and Type of Function for the First 19 α FNOs of the *O*_*h*_ High Spin t_2g_^3^e_g_^2^-^6^A_1g_ State of the FeF_6_^3–^ Complex^a^

*n*_eff_	λ	%loc(Fe)	%loc(F_1_)	type
1.00000	1.00000	100.0	0.0	∼Fe(1s a_1g_)
1.00000	1.00000	100.0	0.0	∼Fe(2s a_1g_)
1.00000	1.00000	100.0	0.0	∼Fe(2p t_1u_)
1.00000	1.00000	100.0	0.0	∼Fe(2p t_1u_)
1.00000	1.00000	100.0	0.0	∼Fe(2p t_1u_)
1.00231	0.99885	99.9	0.0	∼Fe(3s a_1g_)
1.00584	0.99709	99.7	0.1	∼Fe(3p t_1u_)
1.00584	0.99709	99.7	0.0	∼Fe(3p t_1u_)
1.00584	0.99709	99.7	0.1	∼Fe(3p t_1u_)
1.05371	0.97411	97.4	0.6	∼Fe(3d t_2g_)
1.05371	0.97411	97.4	0.6	∼Fe(3d t_2g_)
1.05370	0.97411	97.4	0.1	∼Fe(3d t_2g_)
1.06182	0.97035	97.0	0.6	∼Fe(3d e_g_)
1.06182	0.97035	97.0	0.4	∼Fe(3d e_g_)
0.99760	1.00000	0.0	100.0	∼F_1_(1s a_1_)
1.00000	1.00000	0.0	100.0	∼F_1_(2s a_1_)
1.02849	0.98602	0.7	98.6	∼F_1_(2p e)
1.02849	0.98602	0.7	98.6	∼F_1_(2p e)
1.10107	0.95217	3.9	95.2	∼F_1_(2p a_1_)

a Only one subset of fluorine-center functions is shown. The remaining five are obtained through symmetry operations. ε_*i*_ = 0.85.

**Table 3:** Effective Number of Centers (*n*_eff_), λ Eigenvalues, Percent Localization, and Type of Function for the First 14 β FNOs of the *O*_*h*_ High Spin t_2g_^3^e_g_^2^-^6^A_1*g*_ State in the FeF_6_^3–^ Complex^a^

*n*_eff_	λ	%loc(Fe)	%loc(F_1_)	type
1.00000	1.00000	100.0	0.0	∼Fe(1s a_1g_)
1.00000	1.00000	100.0	0.0	∼Fe(2s a_1g_)
1.00000	1.00000	100.0	0.0	∼Fe(2p t_1u_)
1.00000	1.00000	100.0	0.0	∼Fe(2p t_1u_)
1.00000	1.00000	100.0	0.0	∼Fe(2p t_1u_)
1.00242	0.99879	99.9	0.0	∼Fe(3s a_1g_)
1.00688	0.99658	99.7	0.0	∼Fe(3p t_1u_)
1.00688	0.99658	99.7	0.1	∼Fe(3p t_1u_)
1.00688	0.99658	99.7	0.1	∼Fe(3p t_1u_)
0.99817	1.00091	0.1	100.0	∼F_1_(1s a_1_
1.00000	1.00000	0.0	100.0	∼F_1_(2s a_1_)
1.04995	0.97579	1.5	97.6	∼F_1_(2p e)
1.04992	0.97579	1.5	97.6	∼F_1_(2p e)
1.18414	0.91584	7.6	91.6	∼F_1_(2p a_1_)

a Only F_1_ functions are shown. ε_*i*_ = 0.85.

**Table 4:** Effective Number of Centers (*n*_eff_), λ Eigenvalues, Percent Localization, and Type of Function for the First 17 α FNOs of the *D*_4*h*_ Low Spin e_g_^4^b_2g_-^2^B_2g_ State in the FeF_6_^3–^ Complex^a^

*n*_eff_	λ	%loc(Fe)	%loc(F_1_)	type
1.00000	1.00000	100.0	0.0	∼Fe(1s a_1g_)
1.00000	1.00000	100.0	0.0	∼Fe(2s a_1g_)
1.00000	1.00000	100.0	0.0	∼Fe(2p a_2u_)
1.00000	1.00000	100.0	0.0	∼Fe(2p e_u_)
1.00000	1.00000	100.0	0.0	∼Fe(2p e_u_)
1.00367	0.99817	99.8	0.0	∼Fe(3s a_1g_)
1.00762	0.99621	99.6	0.0	∼Fe(3p a_2u_)
1.00917	0.99544	99.5	0.1	∼Fe(3p e_u_)
1.00917	0.99544	99.5	0.1	∼Fe(3p e_u_)
1.07115	0.96609	96.6	0.9	∼Fe(3d e_g_)
1.07115	0.96609	96.6	0.1	∼Fe(3d e_g_)
1.07273	0.96536	96.5	0.8	∼Fe(3d b_2g_)
0.99864	1.00000	0.1	100.0	∼F_1_(1s a_1_)
1.00000	1.00000	0.0	100.0	∼F_1_(2s a_1_)
1.03199	0.98433	0.8	98.4	∼F_1_(2p b_1_)
1.03252	0.98433	0.6	98.4	∼F_1_(2p b_2_)
1.22460	0.89917	9.0	89.9	∼F_1_(2p a_1_)

a Although axial and equatorial F atoms are nonequivalent, their FNOs differ slightly, and only those for F_1_ are shown. ε_*i*_ = 0.85.

**Table 5:** Effective Number of Centers (*n*_eff_), λ Eigenvalues, Pecent Localization, and Type of Function for the First 16 β FNOs of the *D*_4*h*_ Low Spin e_g_^4^b_2g_-^2^B_2g_ State in the FeF_6_^3–^ Complex^a^

*n*_eff_	λ	%loc(Fe)	%loc(F_1_)	type
1.00000	1.00000	100.0	0.0	∼Fe(1s a_1g_)
1.00000	1.00000	100.0	0.0	∼Fe(2s a_1g_)
1.00000	1.00000	100.0	0.0	∼Fe(2p a_2u_)
1.00000	1.00000	100.0	0.0	∼Fe(2p e_u_)
1.00000	1.00000	100.0	0.0	∼Fe(2p e_u_)
1.00372	0.99814	99.8	0.0	∼Fe(3s a_1g_)
1.00781	0.99612	99.6	0.0	∼Fe(3p a_2u_)
1.00962	0.99522	99.5	0.1	∼Fe(3p e_u_)
1.00962	0.99522	99.5	0.2	∼Fe(3p e_u_)
1.07665	0.96362	96.4	0.6	∼Fe(3d e_g_)
1.07665	0.96362	96.4	0.4	∼Fe(3d e_g_)
0.99806	1.00096	0.1	100.0	∼F_1_(1s a_1_)
1.00000	1.00000	0.0	100.0	∼F_1_(2s a_1_)
1.05735	0.97233	1.8	97.2	∼F_1_(2p b_1_)
1.03245	0.98412	0.6	98.4	∼F_1_(2p b_2_)
1.19627	0.91076	8.0	91.1	∼F_1_(2p a_1_)

a Although axial and equatorial F atoms are nonequivalent, their FNOs differ slightly, and only those for F_1_ are shown. ε_*i*_ = 0.85.

FNOs can be easily classified in both complexes, and all of the 1s to 3p Fe functions delocalize less than 0.5% over the rest of the system. The procedure clearly distinguishes two types of 3p-like orbitals in the *D*_4*h*_ case: the equatorial 3p_*x*_- and 3p_*y*_-like FNOs, with *n*_eff_ = 1.0092, λ = 0.9954, and %loc(Fe) = 99.5, and the marginally less localized axial 3p_*z*_-like FNO, with *n*_eff_ = 1.0076, λ = 0.9962, and %loc(Fe) = 99.6. These values for the three equivalent 3p-like FNOs in the *O*_*h*_ complex are *n*_eff_ = 1.0059, λ = 0.9971, and %loc(Fe) = 99.7.

As far as 3d-like functions are concerned, they also turn out to be quasi-atomic in character, as their localization in the Fe atom (although not so large as in the 1s–3p cases) is greater than 97.0% and 96.5% in the *O*_*h*_ and *D*_4*h*_ complexes, respectively. Notice that these values are clearly smaller than those in the truly core functions, but nevertheless extremely high. Since one-center diagonalizations preserve the point group symmetry, all these functions belong to a specific irreducible representation. For instance, the 3d-like *O*_*h*_ FNOs are of two types: three of them can be identified with the t_2g_ representation and the other two with the e_g_ one, the latter being slightly more delocalized than the former, also in agreement with chemical wisdom. As expected, no 4s function is found.

Similarly, the three F 2p–like FNOs are to be classified in the *C*_4*v*_ group for the *O*_*h*_ complex and in the *C*_2*v*_ group for the *D*_4*h*_ one. It is found that the a_1_ functions that point toward the central iron, which would correspond to the (2c,2e) Fe–F links, are the most delocalized among the set, although always less than 10%. As it can be seen, the low-spin complex is slightly more covalent than the high-spin one, also in agreement with conventional wisdom.

A very rewarding feature emanating from the goodness of the ionic approximation in these simple complexes is that the rs-AdNDP picture is exactly that provided by crystal field theory. If we stay within the one-center image here described (which is stable for a wide range of thresholds), we come to a Fe^3+^ ion surrounded by six fluoride anions. The electronic structure of the metal in its high- and low-spin versions coincides exactly with that coming from conventional crystal or ligand fields: t_2g_^3^e_g_^2^ in the high-spin case, e_g_^4^b_2g_^1^ for the low-spin one. The oxidation state of iron is thus easily set to +3, a result again in agreement with the QTAIM atomic charges (*Q*(Fe) = 2.17, 1.98 *e* for the high and low-spin complexes), the positive Laplacian at the Fe–F bond critical points, and the electron distribution function.

#### PtO_4_^2+^

We end the discussion by considering the tetrahedral complex [PtO_4_]^2+^, an example in which all of the skills of the method developed in this work can be fully illustrated and its power fully demonstrated. This cation has recently raised attention due to the purported X oxidation state of the Pt atom,^57^ and we have already considered it in the previous rs-AdNCP formalism.^31^

**Figure 10:**
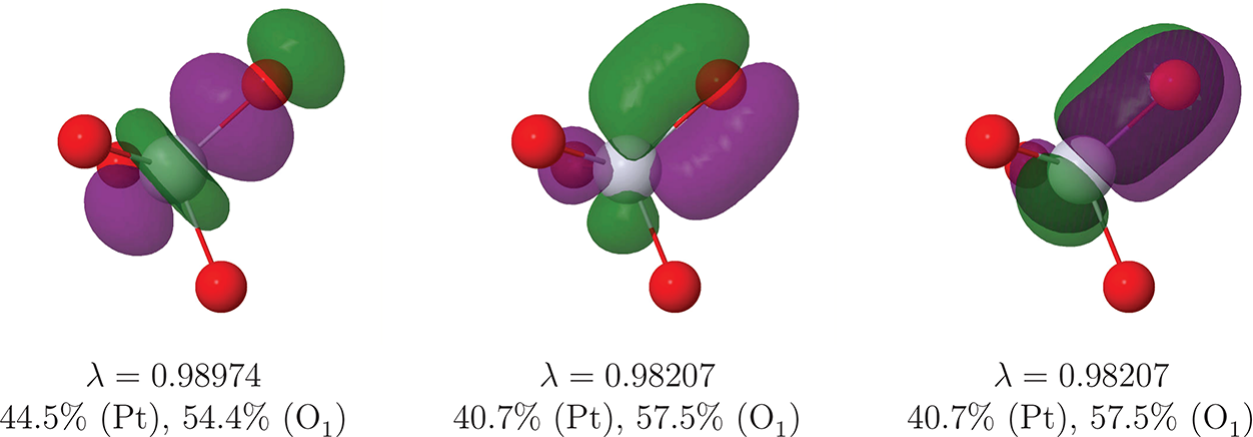
|φ| = 0.05 au isosurface of the σ (left) and π (center and right) Pt–O_1_ FNOs of the PtO_4_^2+^ complex as obtained from heat batch CI (HCI) calculations as described in the text.

We have generated its wave function through heat bath CI (HCI) calculations,^58^ performed with the PySCP suite^55^ and the adZP(Pt)/def2-QZVPD (O) basis sets. Our results are summarized in Figure 10 and Table 6.

**Table 6:** Effective Number of Centers (*n*_eff_), λ Eigenvalues, Percent Localization, and Type of Function for the FNOs of the *T*_*d*_ PtO_4_^2+^ Complex^a^

*n*_eff_	λ	%loc(Pt)	%loc(O_1_)	type
1.00003	0.99998	100.0	0.0	Pt(4f)
1.00006	0.99997	100.0	0.0	Pt(4f)
1.00006	0.99997	100.0	0.0	Pt(4f)
1.00006	0.99997	100.0	0.0	Pt(4f)
1.00009	0.99996	100.0	0.0	Pt(4f)
1.00009	0.99996	100.0	0.0	Pt(4f)
1.00009	0.99996	100.0	0.0	Pt(4f)
1.00010	0.99995	100.0	0.0	Pt(4d)
1.00010	0.99995	100.0	0.0	Pt(4d)
1.00010	0.99995	100.0	0.0	Pt(4d)
1.00011	0.99994	100.0	0.0	Pt(4d)
1.00011	0.99994	100.0	0.0	Pt(4d)
1.00600	0.99701	99.7	0.1	Pt(5s)
1.01234	0.99388	99.4	0.3	Pt(5p)
1.01235	0.99388	99.4	0.1	Pt(5p)
1.01236	0.99387	99.4	0.1	Pt(5p)
1.03081	0.98486	1.2	98.5	O_1_(2s)
2.02123	0.98974	44.5	54.4	Pt–O_1_(σ)
2.01393	0.98207	40.7	57.5	Pt–O_1_(π)
2.01391	0.98206	40.7	57.5	Pt–O_1_(π)

a All the [Kr] Pt and 1s O core orbitals display λ = 1.0000 and are skipped. Only one set of four oxygen functions is shown. ε_*i*_ = 0.90.

This time, 12 (2c,2e) Pt–O links are clearly found, and the metal center displays a fully filled, extremely localized [Xe]4f^14^ core. No valence (6s or 5d) localized orbitals are found at the Pt center. Simultaneously, each O atom bears a σ lone pair with a large 2s character. The remaining 12 two-center links are found in Figure 10, which shows one out of the four equivalent sets of one σ plus two equivalent axi-symmetric π functions. These three links are slightly polarized toward the oxygen. We should notice that the QTAIM Pt charge is +2.84 *e*. If the plain ionic approximation is applied and the 3 × 4 bonding functions are assigned to the O atoms, a X effective oxidation state is really obtained. However, it is clearly seen that the Pt–O bonds are only slightly heteropolar, and that this binary assignment is not clearly justified.

We can relate this image to the more conventional MO picture easily. As we already discussed,^32^ the pure OQS Pt natural orbitals display occupation numbers of 1.14 and 1.34 for the 5d-t_2_ and 5d-e orbitals, respectively, 0.35 for the 6s function, 0.19 for the 6p functions and 0.05 for the 5f-t_2_ manifold, with much smaller contributions from 6d orbitals which are to be assigned to dynamic correlation effects. All but the 5d functions have large contributions from the O ligands. This means that the 12 Pt–O bonds can be understood as a result of the combination of the empty 5d+6s valence +6p+5f-t_2_ Pt virtual space of Pt with 12 fully occupied O 2p functions. The final space is populated with with 24 electrons.

A final point is due. As the SO_4_^2–^ and PtO_4_^2+^ examples have shown, the A–O link (A = S, Pt) displays one σ and two axi-symmetric π contributions. We have found this result to be rather general, and we plan to examine it further in future works. In the sulfate case, the π contributions are so localized over the oxygens (92%) that it is more than sensible to exclude any hypervalency. In the Pt case, on the contrary, all the σ, π links are only slightly polarized, and clear Pt–O triple bonds are observed.

## Summary and Conclusions

Extracting chemical models from high level calculations is both a necessary and at the same time ill-defined enterprise. A working approximation used in the past has been to derive, by whatever means, a quasi minimal basis atomic basis from the computed wave functions that is then used to recover simple chemical pictures through electron counting arguments. This has given rise to the highly successful NBO procedure^16−19^ and to the AdNDP algorithm.^21^ Both are based on relatively arbitrary procedures to build the natural atomic orbital (NAO) set from the one-particle density matrix.

We have previously shown that adopting a real space point of view provides an orbital invariant alternative to the NAO problem that rests only on a predefined exhaustive partition of the molecular space into atomic fragments. Since there are solid physically sound ways to do that (e.g., through the quantum theory of atoms in molecules), we already mimicked the AdNDP prescription in real space by reconstructing atomic density matrices from further order cumulant density matrices in the so-called real space adaptive natural cumulant partitioning (rs-AdNCP).^31^ This procedure takes into account explicitly electron correlation effects but rests on difficult to obtain, nonstandard density matrices that are not immediately output by standard computational packages.

Taking a quantum open systems (OQS) perspective, we here show that the open system fragment one-particle density matrix that we already defined^32^ provides an extremely simple way to access a direct real space analogue of the AdNDP formalism. We have called this procedure the real space adaptive natural density partition method, rs-AdNDP. It provides a set of generalized (*n*c,2e) fragment natural orbitals with which a Lewis structure of a molecular system can be proposed and analyzed.

We have shown that the procedure provides the expected Lewis structures in a number of simple tests, at any level of theory. The method just needs the standard one-particle density matrix, easily accessible from most electronic structure packages, and the atomic overlap matrices that can be obtained also from any of the many QTAIM codes available. The fragment natural orbitals can also be used to assign effective oxidation states. Since the formalism is obviously compatible with its underlying QTAIM basis, all the real space chemical bonding machinery is also compatible with it. This means that Laplacians at bond critical points, delocalization indices, electron distribution functions, or interacting quantum atoms energetic decompositions, to name just a few, all weave a unified and compatible description with the new rs-AdNDP technique.

## Supporting Information Available

Generalized FNOs for all the systems studied, with eigenvalues and localization information, eigenvectors depicted through conventional orbital isosurfaces, and the Cartesian coordinates of the geometries examined (PDF)
