# Supplementary material for: Lewis Structures from Open Quantum Systems Natural Orbitals: Real Space Adaptive Natural Density Partitioning
Source: J Phys Chem A. 2021 Apr 28;125(18):4013–25. doi: 10.1021/acs.jpca.1c01689 (PMC8900138; doi:10.1021/acs.jpca.1c01689)
Supplement: Supplementary file 1 — jp1c01689_si_001.pdf [file jp1c01689_si_001.pdf]

Supporting Information

Lewis Structures from Open Quantum Systems Natural Orbitals: Real Space Adaptive Natural Density Partitioning

Evelio Francisco,* Aurora Costales, María Menéndez-Herrero, and Ángel Martín Pendás*

E-mail:evelio@uniovi.es, ampendas@uniovi.es

Departamento de Química Física y Analítica. Facultad de Química. Universidad de Oviedo. 33006 Oviedo, Spain

Contents

S1 Generalized Fragment Natural Orbitals (FNO): Eigenvalues and localization S4

S1.1 CH_4 . . . S4
S1.2 SO_4^{2-} . . . S4
S1.3 N_2H_2 . . . S5
S1.4 The cis-butadiene plus ethylene Diels-Alder (DA) reaction . . . S6
S1.4.1 IRC Step 00 . . . S6
S1.4.2 IRC Step 02 . . . S7
S1.4.3 IRC Step 04 . . . S9
S1.4.4 IRC Step 06 . . . S10
S1.4.5 IRC Step 08 . . . S12
S1.4.6 IRC Step 10 . . . S13
S1.4.7 IRC Step 12 . . . S15
S1.4.8 IRC Step 14 . . . S16
S1.4.9 IRC Step 16 . . . S18
S1.4.10 IRC Step 18 . . . S19
S1.4.11 IRC Step 20 . . . S21
S1.4.12 IRC Step 22 . . . S22
S1.4.13 IRC Step 24 . . . S24
S1.4.14 IRC Step 26 . . . S25
S1.4.15 IRC Step 28 . . . S27
S1.5 The F^-+CH_3F $\rightarrow$ FCH_3+ F^- reaction . . . S29
S1.5.1 IRC Step 1 . . . S29
S1.5.2 IRC Step 2 . . . S29
S1.5.3 IRC Step 3 . . . S30
S1.5.4 IRC Step 4 . . . S31
S1.5.5 IRC Step 5 . . . S31
S1.5.6 IRC Step 6 . . . S32
S1.5.7 IRC Step 7 . . . S33
S1.5.8 IRC Step 8 . . . S33
S1.5.9 IRC Step 9 . . . S34
S1.5.10 IRC Step 10 . . . S35
S1.5.11 IRC Step 11 . . . S35
S1.5.12 IRC Step 12 . . . S36
S1.5.13 IRC Step 13 . . . S37
S1.5.14 IRC Step 14 . . . S37
S1.5.15 IRC Step 15 . . . S38
S1.5.16 IRC Step 16 . . . S39
S1.5.17 IRC Step 17 . . . S39
S1.5.18 IRC Step 18 . . . S40
S1.5.19 IRC Step 19 . . . S41
S1.5.20 IRC Step 20 . . . S41
S1.5.21 IRC Step 21 . . . S42
S1.5.22 IRC Step 22 . . . S43
S1.5.23 IRC Step 23 . . . S43
S1.5.24 IRC Step 24 . . . S44
S1.6 FeF_6^{3-} . . . S45
S1.6.1 FeF_6^{3-} *Oh* High spin, ($t_{2g}^3e_g^2 -^6 A_{1g}$) . . . S45
S1.6.2 FeF_6^{3-} D_{4h} Low spin ($e_g^4b_{2g}^1 -^2 B_{2g}$) . . . S49
S1.7 PtO_4^{2+} . . . S53

S2 Generalized Fragment Natural Orbitals (FNO): Eigenvectors S55

S2.1 CH_4 . . . S55
S2.2 SO_4^{2-} . . . S55
S2.3 N_2H_2 . . . S56
S2.4 The cis-butadiene plus ethylene Diels-Alder (DA) reaction . . . S57
S2.4.1 Reactants . . . S57
S2.4.2 Transition state . . . S58
S2.4.3 Products . . . S59
S2.5 The F^- + CH_3F $\rightarrow$ FCH_3 + F^- reaction . . . S60
S2.5.1 Reactants/Products . . . S60
S2.5.2 Transition State . . . S61

S2.6 FeF_6^{3-} complex in Oh (high spin) and D_{4h} (low spin) multielectron states. S62
S2.6.1 FeF_6^{3-} Oh High spin, ($t_{2g}^3e_g^2 -^6 A_{1g}$), $\alpha-$orbitals S62
S2.6.2 FeF_6^{3-} Oh High spin, ($t_{2g}^3e_g^2 -^6 A_{1g}$), $\beta-$orbitals S63
S2.6.3 FeF_6^{3-} D_{4h} Low spin ($e_g^4b_{2g}^1 -^2 B_{2g}$), $\alpha-$orbitals S64
S2.6.4 FeF_6^{3-} D_{4h} Low spin ($e_g^4b_{2g}^1 -^2 B_{2g}$) $\beta-$orbitals S65
S2.7 PtO_4^{2+} S66

S3 Cartesian coordinates **S67**
S3.1 CH_4 . . . . S67
S3.2 SO_4^{2-} S67
S3.3 N_2H_2 . . . . S67
S3.4 The cis-butadiene plus ethylene Diels-Alder (DA) reaction S67
S3.4.1 Reactants S67
S3.4.2 Transition state S68
S3.4.3 Products S68
S3.5 The F^- + CH_3F $\rightarrow$ FCH_3 + F^- reaction S68
S3.5.1 Reactants/Products S68
S3.5.2 Transition state S68
S3.6 FeF_6^{3-} complex in Oh (high spin) and D_{4h} (low spin) multielectron states. S69
S3.6.1 FeF_6^{3-} Oh S69
S3.6.2 FeF_6^{3-} D_{4h} S69
S3.7 PtO_4^{2+} S69

S1 Generalized Fragment Natural Orbitals (FNO): Eigenvalues and localization

λ's, percentage of localization and number of atoms expanded by each FNO for the systems examined. Notice that the numbering of FNOs does not coincide with that in the text.

S1.1 CH_4

1-center FNO analysis, $\varepsilon = 0.95000$		
MO	λ	Atoms
1	0.99998	C_1
2-center FNO analysis, $\varepsilon = 0.95000$		
MO	λ	Atoms
2	0.97302	C_1-H_2
3	0.97302	C_1-H_3
4	0.97302	C_1-H_4
5	0.97302	C_1-H_5

Localization and atoms expanded by each FNO						
FNO	Loc_1	Loc_2	Loc_3	Loc_4	Loc_5	n_{eff}
1	100.0%	0.0%	0.0%	0.0%	0.0%	1.00004
2	49.3%	48.0%	0.9%	0.9%	0.9%	2.11105
3	49.3%	0.9%	48.0%	0.9%	0.9%	2.11105
4	49.3%	0.9%	0.9%	48.0%	0.9%	2.11105
5	49.3%	0.9%	0.9%	0.9%	48.0%	2.11105

S1.2 SO_4^{2-}

1-center FNO analysis, $\varepsilon = 0.95000$		
MO	λ	Atoms
1	1.00000	S_1
2	0.99844	S_1
3	0.99770	S_1
4	0.99770	S_1
5	0.99770	S_1
6	1.00000	O_2
7	0.99758	O_2
8	1.00000	O_3
9	0.99758	O_3
10	1.00000	O_4
11	0.99758	O_4
12	1.00000	O_5
13	0.99758	O_5
2-center FNO analysis, $\varepsilon = 0.95000$		
MO	λ	Atoms
14	0.97088	S_1-O_2
15	0.96985	S_1-O_2
16	0.96985	S_1-O_2
17	0.97088	S_1-O_3
18	0.96985	S_1-O_3
19	0.96985	S_1-O_3
20	0.97088	S_1-O_4
21	0.96985	S_1-O_4
22	0.96985	S_1-O_4
23	0.97088	S_1-O_5
24	0.96985	S_1-O_5
25	0.96985	S_1-O_5

Localization and atoms expanded by each FNO						
FNO	Loc_1	Loc_2	Loc_3	Loc_4	Loc_5	n_{eff}
1	100.0%	-0.0%	-0.0%	-0.0%	-0.0%	1.00000
2	99.8%	0.0%	0.0%	0.0%	0.0%	1.00313
3	99.8%	0.1%	0.1%	0.1%	0.1%	1.00462
4	99.8%	0.1%	0.0%	0.0%	0.1%	1.00462
5	99.8%	0.0%	0.1%	0.1%	0.0%	1.00462
6	-0.0%	100.0%	0.0%	0.0%	0.0%	1.00000
7	0.1%	99.8%	0.0%	0.0%	0.0%	1.00486
8	-0.0%	0.0%	100.0%	0.0%	0.0%	1.00000
9	0.1%	0.0%	99.8%	0.0%	0.0%	1.00486
10	-0.0%	0.0%	0.0%	100.0%	0.0%	1.00000
11	0.1%	0.0%	0.0%	99.8%	0.0%	1.00486
12	-0.0%	0.0%	0.0%	0.0%	100.0%	1.00000
13	0.1%	0.0%	0.0%	0.0%	99.8%	1.00486
14	18.9%	78.1%	1.0%	1.0%	1.0%	1.54617
15	4.5%	92.4%	0.6%	1.3%	1.3%	1.16748
16	4.5%	92.4%	1.5%	0.8%	0.8%	1.16748
17	18.9%	1.0%	78.1%	1.0%	1.0%	1.54617
18	4.5%	0.6%	92.4%	1.3%	1.3%	1.16748
19	4.5%	1.5%	92.4%	0.8%	0.8%	1.16748
20	18.9%	1.0%	1.0%	78.1%	1.0%	1.54617
21	4.5%	1.3%	1.3%	92.4%	0.6%	1.16748
22	4.5%	0.8%	0.8%	92.4%	1.5%	1.16748
23	18.9%	1.0%	1.0%	1.0%	78.1%	1.54617
24	4.5%	1.3%	1.3%	0.6%	92.4%	1.16748
25	4.5%	0.8%	0.8%	1.5%	92.4%	1.16748

S1.3 N_2H_2

1-center FNO analysis, $\varepsilon = 0.85000$		
MO	λ	Atoms
1	1.00000	N_1
2	0.98541	N_1
3	1.00000	N_2
4	0.98541	N_2
2-center FNO analysis, $\varepsilon = 0.85000$		
MO	λ	Atoms
5	0.95550	N_1-N_2
6	0.87797	N_1-N_2
7	0.98805	N_1-H_3
8	0.98805	N_2-H_4

Localization and atoms expanded by each FNO					
FNO	Loc_1	Loc_2	Loc_3	Loc_4	n_{eff}
1	100.0%	0.0%	0.0%	0.0%	1.00000
2	98.8%	0.8%	0.3%	0.1%	1.02348
3	0.0%	100.0%	0.0%	0.0%	1.00000
4	0.8%	98.8%	0.1%	0.3%	1.02348
5	49.5%	49.5%	0.5%	0.5%	2.04294
6	49.3%	49.3%	0.7%	0.7%	2.04294
7	69.4%	1.1%	29.4%	0.1%	1.76675
8	1.1%	69.4%	0.1%	29.4%	1.76675

S1.4 The cis-butadiene plus ethylene Diels-Alder (DA) reaction

The IRC has been projected onto the C-C distance (R) of any of the two single σ bonds that are formed during the cycloaddition. In the next subsections the evolution with R of the eigenvalues and effective number of centers for all FNOs is show.

S1.4.1 IRC Step 00

1-center FNO analysis, $\varepsilon = 0.90000$		
MO	λ	Atoms
1	0.99999	C_1
2	0.99999	C_2
3	0.99999	C_5
4	0.99999	C_6
5	0.99999	C_{11}
6	0.99999	C_{12}
**2-center FNO analysis, $\varepsilon = 0.90000$**		
MO	λ	Atoms
7	0.97468	C_1-C_2
8	0.97485	C_1-H_3
9	0.98210	C_1-C_5
10	0.97473	C_2-H_4
11	0.98206	C_2-C_6
12	0.97550	C_5-H_7
13	0.98067	C_5-H_9
14	0.97549	C_6-H_8
15	0.98067	C_6-H_{10}
16	0.98360	$C_{11}-C_{12}$
17	0.92050	$C_{11}-C_{12}$
18	0.98030	$C_{11}-H_{13}$
19	0.97902	$C_{11}-H_{15}$
20	0.98026	$C_{12}-H_{14}$
21	0.97903	$C_{12}-H_{16}$
**3-center FNO analysis, $\varepsilon = 0.90000$**		
MO	λ	Atoms
22	0.94317	$C_1-C_2-C_5$
23	0.94317	$C_1-C_2-C_6$

Localization and atoms expanded by each FNO									
FNO	Loc_1	Loc_2	Loc_3	Loc_4	Loc_5	Loc_6	Loc_7	Loc_8	Loc_9
1	100.0%	0.0%	0.0%	0.0%	0.0%	0.0%	0.0%	0.0%	0.0%
2	0.0%	100.0%	0.0%	0.0%	0.0%	0.0%	0.0%	0.0%	0.0%
3	0.0%	0.0%	0.0%	0.0%	100.0%	0.0%	0.0%	0.0%	0.0%
4	0.0%	0.0%	0.0%	0.0%	0.0%	100.0%	0.0%	0.0%	0.0%
5	-0.0%	0.0%	0.0%	0.0%	0.0%	0.0%	0.0%	0.0%	0.0%
6	-0.0%	0.0%	0.0%	0.0%	0.0%	0.0%	0.0%	0.0%	0.0%
7	48.7%	48.7%	0.5%	0.5%	0.6%	0.6%	0.1%	0.1%	0.1%
8	51.8%	0.9%	45.7%	0.2%	1.0%	0.1%	0.1%	0.0%	0.2%
9	49.2%	0.4%	0.4%	0.1%	49.0%	0.1%	0.4%	0.0%	0.4%
10	0.9%	51.8%	0.2%	45.7%	0.1%	1.0%	0.0%	0.1%	0.0%
11	0.4%	49.2%	0.1%	0.4%	0.1%	49.0%	0.0%	0.4%	0.0%
12	0.8%	0.1%	0.1%	0.0%	53.0%	0.1%	44.5%	0.3%	0.6%
13	0.9%	0.1%	0.2%	0.0%	53.1%	0.0%	0.6%	0.0%	44.9%
14	0.1%	0.8%	0.0%	0.1%	0.1%	53.0%	0.3%	44.5%	0.0%
15	0.1%	0.9%	0.0%	0.2%	0.0%	53.1%	0.0%	0.6%	0.0%
16	0.0%	0.0%	0.0%	0.0%	0.0%	0.0%	0.0%	0.0%	0.0%
17	0.2%	0.2%	0.0%	0.0%	0.4%	0.4%	0.2%	0.2%	0.0%
18	0.0%	0.0%	0.0%	0.0%	0.1%	0.0%	0.1%	0.0%	0.0%
19	0.1%	0.0%	0.0%	0.0%	0.2%	0.0%	0.0%	0.0%	0.0%
20	0.0%	0.0%	0.0%	0.0%	0.0%	0.1%	0.0%	0.1%	0.0%
21	0.0%	0.1%	0.0%	0.0%	0.0%	0.2%	0.0%	0.0%	0.0%
22	46.1%	7.6%	1.6%	0.2%	40.6%	0.3%	1.2%	0.0%	1.4%
23	7.7%	46.1%	0.2%	1.6%	0.3%	40.6%	0.0%	1.2%	0.0%

Localization and atoms expanded by each FNO								
FNO	Loc_{10}	Loc_{11}	Loc_{12}	Loc_{13}	Loc_{14}	Loc_{15}	Loc_{16}	n_{eff}
1	0.0%	0.0%	0.0%	0.0%	0.0%	0.0%	0.0%	1.00002
2	0.0%	0.0%	0.0%	0.0%	0.0%	0.0%	0.0%	1.00002
3	0.0%	0.0%	0.0%	0.0%	0.0%	0.0%	0.0%	1.00002
4	0.0%	0.0%	0.0%	0.0%	0.0%	0.0%	0.0%	1.00002
5	0.0%	100.0%	0.0%	0.0%	0.0%	0.0%	0.0%	1.00002
6	0.0%	0.0%	100.0%	0.0%	0.0%	0.0%	0.0%	1.00002
7	0.1%	0.0%	0.0%	0.0%	0.0%	0.0%	0.0%	2.10472
8	0.0%	0.0%	0.0%	0.0%	0.0%	0.0%	0.0%	2.09531
9	0.0%	0.0%	0.0%	0.0%	0.0%	0.0%	0.0%	2.07328
10	0.2%	0.0%	0.0%	0.0%	0.0%	0.0%	0.0%	2.09585
11	0.4%	0.0%	0.0%	0.0%	0.0%	0.0%	0.0%	2.07342
12	0.0%	0.2%	0.0%	0.1%	0.0%	0.0%	0.0%	2.08528
13	0.0%	0.0%	0.0%	0.0%	0.0%	0.0%	0.0%	2.06456
14	0.6%	0.0%	0.2%	0.0%	0.1%	0.0%	0.0%	2.08531
15	44.9%	0.0%	0.0%	0.0%	0.0%	0.0%	0.0%	2.06460
16	0.0%	49.2%	49.2%	0.4%	0.4%	0.4%	0.4%	2.06698
17	0.0%	46.0%	46.0%	1.6%	1.6%	1.5%	1.5%	2.35483
18	0.0%	52.7%	0.9%	45.3%	0.1%	0.6%	0.1%	2.06879
19	0.0%	53.1%	0.9%	0.6%	0.1%	44.8%	0.1%	2.07138
20	0.0%	0.9%	52.7%	0.1%	45.3%	0.1%	0.6%	2.06898
21	0.0%	0.9%	53.1%	0.1%	0.6%	0.1%	44.8%	2.07138
22	0.0%	0.5%	0.1%	0.0%	0.0%	0.3%	0.0%	2.60685
23	1.4%	0.1%	0.5%	0.0%	0.0%	0.0%	0.3%	2.60772

S1.4.2 IRC Step 02

1-center FNO analysis, $\varepsilon = 0.90000$		
MO	λ	Atoms
1	0.99999	C_1
2	0.99999	C_2
3	0.99999	C_5
4	0.99999	C_6
5	0.99999	C_{11}
6	0.99999	C_{12}
2-center FNO analysis, $\varepsilon = 0.90000$		
MO	λ	Atoms
7	0.97462	$C_1{-}C_2$
8	0.97483	$C_1{-}H_3$
9	0.98201	$C_1{-}C_5$
10	0.97469	$C_2{-}H_4$
11	0.98196	$C_2{-}C_6$
12	0.97511	$C_5{-}H_7$
13	0.98063	$C_5{-}H_9$
14	0.97508	$C_6{-}H_8$
15	0.98064	$C_6{-}H_{10}$
16	0.98347	$C_{11}{-}C_{12}$
17	0.91860	$C_{11}{-}C_{12}$
18	0.97975	$C_{11}{-}H_{13}$
19	0.97869	$C_{11}{-}H_{15}$
20	0.97976	$C_{12}{-}H_{14}$
21	0.97871	$C_{12}{-}H_{16}$
3-center FNO analysis, $\varepsilon = 0.90000$		
MO	λ	Atoms
22	0.94193	$C_1{-}C_2{-}C_5$
23	0.94192	$C_1{-}C_2{-}C_6$

Localization and atoms expanded by each FNO									
FNO	Loc_1	Loc_2	Loc_3	Loc_4	Loc_5	Loc_6	Loc_7	Loc_8	Loc_9
1	100.0%	0.0%	0.0%	0.0%	0.0%	0.0%	0.0%	0.0%	0.0%
2	0.0%	100.0%	0.0%	0.0%	0.0%	0.0%	0.0%	0.0%	0.0%
3	0.0%	0.0%	0.0%	0.0%	100.0%	0.0%	0.0%	0.0%	0.0%
4	0.0%	0.0%	0.0%	0.0%	0.0%	100.0%	0.0%	0.0%	0.0%
5	-0.0%	0.0%	0.0%	0.0%	0.0%	0.0%	0.0%	0.0%	0.0%
6	-0.0%	0.0%	0.0%	0.0%	0.0%	0.0%	0.0%	0.0%	0.0%
7	48.7%	48.7%	0.5%	0.5%	0.6%	0.6%	0.1%	0.1%	0.1%
8	51.8%	0.9%	45.7%	0.2%	1.0%	0.1%	0.1%	0.0%	0.2%
9	49.2%	0.4%	0.4%	0.1%	49.0%	0.1%	0.4%	0.0%	0.4%
10	0.9%	51.8%	0.2%	45.7%	0.1%	1.0%	0.0%	0.1%	0.0%
11	0.4%	49.2%	0.1%	0.4%	0.1%	49.0%	0.0%	0.4%	0.0%
12	0.8%	0.1%	0.1%	0.0%	53.0%	0.1%	44.5%	0.3%	0.6%
13	0.9%	0.1%	0.2%	0.0%	53.1%	0.0%	0.6%	0.0%	44.9%
14	0.1%	0.8%	0.0%	0.1%	0.1%	53.0%	0.3%	44.5%	0.0%
15	0.1%	0.9%	0.0%	0.2%	0.0%	53.1%	0.0%	0.6%	0.0%
16	0.0%	0.0%	0.0%	0.0%	0.0%	0.0%	0.0%	0.0%	0.0%
17	0.3%	0.3%	0.0%	0.0%	0.5%	0.5%	0.2%	0.2%	0.0%
18	0.0%	0.0%	0.0%	0.0%	0.1%	0.0%	0.1%	0.0%	0.0%
19	0.1%	0.0%	0.0%	0.0%	0.2%	0.0%	0.0%	0.0%	0.0%
20	0.0%	0.0%	0.0%	0.0%	0.0%	0.1%	0.0%	0.1%	0.0%
21	0.0%	0.1%	0.0%	0.0%	0.0%	0.2%	0.0%	0.0%	0.0%
22	46.0%	7.8%	1.6%	0.2%	40.4%	0.3%	1.2%	0.0%	1.4%
23	7.8%	46.0%	0.2%	1.6%	0.3%	40.4%	0.0%	1.2%	0.0%

Localization and atoms expanded by each FNO								
FNO	Loc_{10}	Loc_{11}	Loc_{12}	Loc_{13}	Loc_{14}	Loc_{15}	Loc_{16}	n_{eff}
1	0.0%	0.0%	0.0%	0.0%	0.0%	0.0%	0.0%	1.00002
2	0.0%	0.0%	0.0%	0.0%	0.0%	0.0%	0.0%	1.00002
3	0.0%	0.0%	0.0%	0.0%	0.0%	0.0%	0.0%	1.00002
4	0.0%	0.0%	0.0%	0.0%	0.0%	0.0%	0.0%	1.00002
5	0.0%	100.0%	0.0%	0.0%	0.0%	0.0%	0.0%	1.00002
6	0.0%	0.0%	100.0%	0.0%	0.0%	0.0%	0.0%	1.00002
7	0.1%	0.0%	0.0%	0.0%	0.0%	0.0%	0.0%	2.10497
8	0.0%	0.0%	0.0%	0.0%	0.0%	0.0%	0.0%	2.09543
9	0.0%	0.0%	0.0%	0.0%	0.0%	0.0%	0.0%	2.07364
10	0.2%	0.0%	0.0%	0.0%	0.0%	0.0%	0.0%	2.09606
11	0.4%	0.0%	0.0%	0.0%	0.0%	0.0%	0.0%	2.07386
12	0.0%	0.2%	0.0%	0.1%	0.0%	0.0%	0.0%	2.08693
13	0.0%	0.0%	0.0%	0.0%	0.0%	0.0%	0.0%	2.06477
14	0.6%	0.0%	0.2%	0.0%	0.1%	0.0%	0.0%	2.08710
15	44.9%	0.0%	0.0%	0.0%	0.0%	0.0%	0.0%	2.06476
16	0.0%	49.2%	49.2%	0.4%	0.4%	0.4%	0.4%	2.06751
17	0.0%	45.9%	45.9%	1.6%	1.6%	1.5%	1.5%	2.36453
18	0.0%	52.7%	0.9%	45.3%	0.1%	0.6%	0.1%	2.07132
19	0.0%	53.0%	0.9%	0.6%	0.1%	44.9%	0.1%	2.07304
20	0.0%	0.9%	52.7%	0.1%	45.3%	0.1%	0.6%	2.07128
21	0.0%	0.9%	53.0%	0.1%	0.6%	0.1%	44.9%	2.07294
22	0.0%	0.6%	0.2%	0.0%	0.0%	0.3%	0.0%	2.61928
23	1.4%	0.2%	0.6%	0.0%	0.0%	0.0%	0.3%	2.62014

S1.4.3 IRC Step 04

1-center FNO analysis, $\varepsilon = 0.90000$		
MO	λ	Atoms
1	0.99999	C_1
2	0.99999	C_2
3	0.99999	C_5
4	0.99999	C_6
5	0.99999	C_{11}
6	0.99999	C_{12}
2-center FNO analysis, $\varepsilon = 0.90000$		
MO	λ	Atoms
7	0.97462	C_1-C_2
8	0.97483	C_1-H_3
9	0.98201	C_1-C_5
10	0.97469	C_2-H_4
11	0.98196	C_2-C_6
12	0.97511	C_5-H_7
13	0.98063	C_5-H_9
14	0.97508	C_6-H_8
15	0.98064	C_6-H_{10}
16	0.98347	$C_{11}-C_{12}$
17	0.91860	$C_{11}-C_{12}$
18	0.97975	$C_{11}-H_{13}$
19	0.97869	$C_{11}-H_{15}$
20	0.97976	$C_{12}-H_{14}$
21	0.97871	$C_{12}-H_{16}$
3-center FNO analysis, $\varepsilon = 0.90000$		
MO	λ	Atoms
22	0.94193	$C_1-C_2-C_5$
23	0.94192	$C_1-C_2-C_6$

Localization and atoms expanded by each FNO									
FNO	Loc_1	Loc_2	Loc_3	Loc_4	Loc_5	Loc_6	Loc_7	Loc_8	Loc_9
1	100.0%	0.0%	0.0%	0.0%	0.0%	0.0%	0.0%	0.0%	0.0%
2	0.0%	100.0%	0.0%	0.0%	0.0%	0.0%	0.0%	0.0%	0.0%
3	0.0%	0.0%	0.0%	0.0%	100.0%	0.0%	0.0%	0.0%	0.0%
4	0.0%	0.0%	0.0%	0.0%	0.0%	100.0%	0.0%	0.0%	0.0%
5	0.0%	0.0%	0.0%	0.0%	0.0%	0.0%	0.0%	0.0%	0.0%
6	-0.0%	0.0%	0.0%	0.0%	0.0%	0.0%	0.0%	0.0%	0.0%
7	48.7%	48.7%	0.5%	0.5%	0.6%	0.6%	0.1%	0.1%	0.1%
8	51.8%	0.9%	45.7%	0.2%	1.0%	0.1%	0.1%	0.0%	0.2%
9	49.1%	0.4%	0.4%	0.1%	49.0%	0.1%	0.4%	0.0%	0.4%
10	0.9%	51.8%	0.2%	45.7%	0.1%	1.0%	0.0%	0.1%	0.0%
11	0.4%	49.1%	0.1%	0.4%	0.1%	49.0%	0.0%	0.4%	0.0%
12	0.8%	0.1%	0.1%	0.0%	53.0%	0.1%	44.5%	0.3%	0.6%
13	0.9%	0.1%	0.2%	0.0%	53.1%	0.0%	0.6%	0.0%	44.9%
14	0.1%	0.8%	0.0%	0.1%	0.1%	53.0%	0.3%	44.5%	0.0%
15	0.1%	0.9%	0.0%	0.2%	0.0%	53.1%	0.0%	0.6%	0.0%
16	0.0%	0.0%	0.0%	0.0%	0.0%	0.0%	0.0%	0.0%	0.0%
17	0.3%	0.3%	0.0%	0.0%	0.6%	0.6%	0.3%	0.3%	0.0%
18	0.0%	0.0%	0.0%	0.0%	0.1%	0.0%	0.1%	0.0%	0.0%
19	0.1%	0.0%	0.0%	0.0%	0.2%	0.0%	0.0%	0.0%	0.0%
20	0.0%	0.0%	0.0%	0.0%	0.0%	0.1%	0.0%	0.1%	0.0%
21	0.0%	0.1%	0.0%	0.0%	0.0%	0.2%	0.0%	0.0%	0.0%
22	46.0%	7.9%	1.6%	0.2%	40.1%	0.3%	1.2%	0.0%	1.4%
23	7.9%	46.0%	0.2%	1.6%	0.3%	40.1%	0.0%	1.2%	0.0%

Localization and atoms expanded by each FNO								
FNO	Loc_{10}	Loc_{11}	Loc_{12}	Loc_{13}	Loc_{14}	Loc_{15}	Loc_{16}	n_{eff}
1	0.0%	0.0%	0.0%	0.0%	0.0%	0.0%	0.0%	1.00002
2	0.0%	0.0%	0.0%	0.0%	0.0%	0.0%	0.0%	1.00002
3	0.0%	0.0%	0.0%	0.0%	0.0%	0.0%	0.0%	1.00002
4	0.0%	0.0%	0.0%	0.0%	0.0%	0.0%	0.0%	1.00002
5	0.0%	100.0%	0.0%	0.0%	0.0%	0.0%	0.0%	1.00002
6	0.0%	0.0%	100.0%	0.0%	0.0%	0.0%	0.0%	1.00002
7	0.1%	0.0%	0.0%	0.0%	0.0%	0.0%	0.0%	2.10527
8	0.0%	0.0%	0.0%	0.0%	0.0%	0.0%	0.0%	2.09566
9	0.0%	0.0%	0.0%	0.0%	0.0%	0.0%	0.0%	2.07420
10	0.2%	0.0%	0.0%	0.0%	0.0%	0.0%	0.0%	2.09626
11	0.4%	0.0%	0.0%	0.0%	0.0%	0.0%	0.0%	2.07426
12	0.0%	0.2%	0.0%	0.1%	0.0%	0.0%	0.0%	2.08903
13	0.0%	0.0%	0.0%	0.0%	0.0%	0.0%	0.0%	2.06505
14	0.6%	0.0%	0.2%	0.0%	0.1%	0.0%	0.0%	2.08950
15	44.9%	0.0%	0.0%	0.0%	0.0%	0.0%	0.0%	2.04459
16	0.0%	49.2%	49.2%	0.4%	0.4%	0.4%	0.4%	2.06771
17	0.0%	45.8%	45.8%	1.6%	1.6%	1.5%	1.5%	2.37475
18	0.0%	52.6%	0.9%	45.3%	0.1%	0.6%	0.1%	2.07430
19	0.0%	53.0%	0.9%	0.6%	0.1%	44.9%	0.1%	2.07440
20	0.0%	0.9%	52.6%	0.1%	45.3%	0.1%	0.6%	2.07451
21	0.0%	0.9%	53.0%	0.1%	0.6%	0.1%	44.9%	2.07400
22	0.0%	0.7%	0.2%	0.0%	0.0%	0.3%	0.0%	2.63467
23	1.4%	0.2%	0.7%	0.0%	0.0%	0.0%	0.3%	2.63497

S1.4.4 IRC Step 06

1-center FNO analysis, $\varepsilon = 0.90000$		
MO	λ	Atoms
1	0.99999	C_1
2	0.99999	C_2
3	0.99999	C_5
4	0.99999	C_6
5	0.99999	C_{11}
6	0.99999	C_{12}
2-center FNO analysis, $\varepsilon = 0.90000$		
MO	λ	Atoms
7	0.97446	$C_1{-}C_2$
8	0.97478	$C_1{-}H_3$
9	0.98173	$C_1{-}C_5$
10	0.97472	$C_2{-}H_4$
11	0.98181	$C_2{-}C_6$
12	0.97388	$C_5{-}H_7$
13	0.98055	$C_5{-}H_9$
14	0.97388	$C_6{-}H_8$
15	0.98053	$C_6{-}H_{10}$
16	0.98328	$C_{11}{-}C_{12}$
17	0.91386	$C_{11}{-}C_{12}$
18	0.97845	$C_{11}{-}H_{13}$
19	0.97798	$C_{11}{-}H_{15}$
20	0.97844	$C_{12}{-}H_{14}$
21	0.97801	$C_{12}{-}H_{16}$
3-center FNO analysis, $\varepsilon = 0.90000$		
MO	λ	Atoms
22	0.93882	$C_1{-}C_2{-}C_5$
23	0.93881	$C_1{-}C_2{-}C_6$

Localization and atoms expanded by each FNO									
FNO	Loc_1	Loc_2	Loc_3	Loc_4	Loc_5	Loc_6	Loc_7	Loc_8	Loc_9
1	100.0%	0.0%	0.0%	0.0%	0.0%	0.0%	0.0%	0.0%	0.0%
2	0.0%	100.0%	0.0%	0.0%	0.0%	0.0%	0.0%	0.0%	0.0%
3	0.0%	0.0%	0.0%	0.0%	100.0%	0.0%	0.0%	0.0%	0.0%
4	0.0%	0.0%	0.0%	0.0%	0.0%	100.0%	0.0%	0.0%	0.0%
5	0.0%	0.0%	0.0%	0.0%	0.0%	0.0%	0.0%	0.0%	0.0%
6	0.0%	0.0%	0.0%	0.0%	0.0%	0.0%	0.0%	0.0%	0.0%
7	48.7%	48.7%	0.5%	0.5%	0.6%	0.6%	0.1%	0.1%	0.1%
8	51.8%	0.9%	45.7%	0.2%	1.0%	0.1%	0.1%	0.0%	0.2%
9	49.1%	0.4%	0.4%	0.1%	49.1%	0.1%	0.4%	0.0%	0.4%
10	0.9%	51.8%	0.2%	45.7%	0.1%	1.0%	0.0%	0.1%	0.0%
11	0.4%	49.1%	0.1%	0.4%	0.1%	49.1%	0.0%	0.4%	0.0%
12	0.8%	0.1%	0.1%	0.0%	53.0%	0.1%	44.4%	0.3%	0.6%
13	0.9%	0.1%	0.2%	0.0%	53.1%	0.0%	0.6%	0.0%	44.9%
14	0.1%	0.8%	0.0%	0.1%	0.1%	53.0%	0.3%	44.4%	0.0%
15	0.1%	0.9%	0.0%	0.2%	0.0%	53.1%	0.0%	0.6%	0.0%
16	0.0%	0.0%	0.0%	0.0%	0.0%	0.0%	0.0%	0.0%	0.0%
17	0.4%	0.3%	0.0%	0.0%	0.6%	0.6%	0.3%	0.3%	0.0%
18	0.0%	0.0%	0.0%	0.0%	0.2%	0.0%	0.2%	0.0%	0.0%
19	0.1%	0.0%	0.0%	0.0%	0.2%	0.0%	0.0%	0.0%	0.0%
20	0.0%	0.0%	0.0%	0.0%	0.0%	0.2%	0.0%	0.2%	0.0%
21	0.0%	0.1%	0.0%	0.0%	0.0%	0.2%	0.0%	0.0%	0.0%
22	46.0%	8.1%	1.6%	0.2%	39.8%	0.3%	1.2%	0.0%	1.4%
23	8.2%	45.9%	0.2%	1.6%	0.3%	39.8%	0.0%	1.2%	0.0%

Localization and atoms expanded by each FNO								
FNO	Loc_{10}	Loc_{11}	Loc_{12}	Loc_{13}	Loc_{14}	Loc_{15}	Loc_{16}	n_{eff}
1	0.0%	0.0%	0.0%	0.0%	0.0%	0.0%	0.0%	1.00002
2	0.0%	0.0%	0.0%	0.0%	0.0%	0.0%	0.0%	1.00002
3	0.0%	0.0%	0.0%	0.0%	0.0%	0.0%	0.0%	1.00002
4	0.0%	0.0%	0.0%	0.0%	0.0%	0.0%	0.0%	1.00002
5	0.0%	100.0	0.0%	0.0%	0.0%	0.0%	0.0%	1.00002
6	0.0%	0.0%	100.0%	0.0%	0.0%	0.0%	0.0%	1.00002
7	0.1%	0.0%	0.0%	0.0%	0.0%	0.0%	0.0%	2.10565
8	0.0%	0.0%	0.0%	0.0%	0.0%	0.0%	0.0%	2.09572
9	0.0%	0.0%	0.0%	0.0%	0.0%	0.0%	0.0%	2.07485
10	0.2%	0.0%	0.0%	0.0%	0.0%	0.0%	0.0%	2.09599
11	0.4%	0.0%	0.0%	0.0%	0.0%	0.0%	0.0%	2.07451
12	0.0%	0.3%	0.0%	0.1%	0.0%	0.0%	0.0%	2.02040
13	0.0%	0.0%	0.0%	0.0%	0.0%	0.0%	0.0%	2.06526
14	0.6%	0.0%	0.3%	0.0%	0.1%	0.0%	0.0%	2.09208
15	444.9%	0.0%	0.0%	0.0%	0.0%	0.0%	0.0%	2.06532
16	0.0%	49.2%	49.2%	0.4%	0.4%	0.4%	0.4%	2.06832
17	0.0%	45.7%	45.7%	1.6%	1.6%	1.5%	1.5%	2.38888
18	0.0%	52.5%	0.9%	45.3%	0.1%	0.6%	0.1%	2.07290
19	0.0%	52.9%	0.9%	0.6%	0.1%	44.9%	0.1%	2.07637
20	0.0%	0.9%	52.5%	0.1%	45.3%	0.1%	0.6%	2.07731
21	0.0%	0.9%	52.9%	0.1%	0.6%	0.1%	44.9%	2.07627
22	0.0%	0.8%	0.3%	0.1%	0.0%	0.3%	0.0%	2.65435
23	1.4%	0.3%	0.8%	0.0%	0.1%	0.0%	0.3%	2.65441

S1.4.5 IRC Step 08

1-center FNO analysis, $\varepsilon = 0.90000$		
MO	λ	Atoms
1	0.99999	C_1
2	0.99999	C_2
3	0.99999	C_5
4	0.99999	C_6
5	0.99999	C_{11}
6	0.99999	C_{12}
2-center FNO analysis, $\varepsilon = 0.90000$		
MO	λ	Atoms
7	0.97434	C_1–C_2
8	0.97484	C_1–H_3
9	0.98152	C_1–C_5
10	0.97478	C_2–H_4
11	0.98148	C_2–C_6
12	0.97277	C_5–H_7
13	0.98042	C_5–H_9
14	0.97276	C_6–H_8
15	0.98042	C_6–H_{10}
16	0.98298	C_{11}–C_{12}
17	0.91032	C_{11}–C_{12}
18	0.97772	C_{11}–H_{13}
19	0.97729	C_{11}–H_{15}
20	0.97771	C_{12}–H_{14}
21	0.97728	C_{12}–H_{16}
3-center FNO analysis, $\varepsilon = 0.90000$		
MO	λ	Atoms
22	0.93703	C_1–C_2–C_5
23	0.93703	C_1–C_2–C_6

Localization and atoms expanded by each FNO									
FNO	Loc_1	Loc_2	Loc_3	Loc_4	Loc_5	Loc_6	Loc_7	Loc_8	Loc_9
1	100.0%	0.0%	0.0%	0.0%	0.0%	0.0%	0.0%	0.0%	0.0%
2	0.0%	100.0%	0.0%	0.0%	0.0%	0.0%	0.0%	0.0%	0.0%
3	0.0%	0.0%	0.0%	0.0%	100.0%	0.0%	0.0%	0.0%	0.0%
4	0.0%	0.0%	0.0%	0.0%	0.0%	100.0%	0.0%	0.0%	0.0%
5	0.0%	0.0%	0.0%	0.0%	0.0%	0.0%	0.0%	0.0%	0.0%
6	0.0%	0.0%	0.0%	0.0%	0.0%	0.0%	0.0%	0.0%	0.0%
7	48.7%	48.7%	0.5%	0.5%	0.6%	0.6%	0.1%	0.1%	0.1%
8	51.8%	0.9%	45.7%	0.2%	1.0%	0.1%	0.1%	0.0%	0.2%
9	49.0%	0.5%	0.4%	0.1%	49.1%	0.1%	0.4%	0.0%	0.4%
10	0.9%	51.8%	0.2%	45.7%	0.1%	1.0%	0.0%	0.1%	0.0%
11	0.5%	49.0%	0.1%	0.4%	0.1%	49.1%	0.0%	0.4%	0.0%
12	0.8%	0.1%	0.1%	0.0%	52.9%	0.1%	44.4%	0.3%	0.6%
13	0.9%	0.1%	0.2%	0.0%	53.1%	0.0%	0.6%	0.0%	45.0%
14	0.1%	0.8%	0.0%	0.1%	0.1%	52.9%	0.3%	44.4%	0.0%
15	0.1%	0.9%	0.0%	0.2%	0.0%	53.1%	0.0%	0.6%	0.0%
16	0.0%	0.0%	0.0%	0.0%	0.0%	0.0%	0.0%	0.0%	0.0%
17	0.4%	0.4%	0.0%	0.0%	0.8%	0.8%	0.3%	0.3%	0.0%
18	0.0%	0.0%	0.0%	0.0%	0.2%	0.0%	0.2%	0.0%	0.0%
19	0.1%	0.0%	0.0%	0.0%	0.3%	0.0%	0.0%	0.0%	0.0%
20	-0.0%	0.0%	0.0%	0.0%	0.0%	0.2%	0.0%	0.2%	0.0%
21	0.0%	0.1%	0.0%	0.0%	0.0%	0.3%	0.0%	0.0%	0.0%
22	45.9%	8.5%	1.6%	0.2%	39.3%	0.3%	1.1%	0.0%	1.3%
23	8.5%	45.9%	0.2%	1.6%	0.3%	39.3%	0.0%	1.1%	0.0%

Localization and atoms expanded by each FNO								
FNO	Loc_{10}	Loc_{11}	Loc_{12}	Loc_{13}	Loc_{14}	Loc_{15}	Loc_{16}	n_{eff}
1	0.0%	0.0%	0.0%	0.0%	0.0%	0.0%	0.0%	1.00002
2	0.0%	0.0%	0.0%	0.0%	0.0%	0.0%	0.0%	1.00002
3	0.0%	0.0%	0.0%	0.0%	0.0%	0.0%	0.0%	1.00002
4	0.0%	0.0%	0.0%	0.0%	0.0%	0.0%	0.0%	1.00002
5	0.0%	100.0%	0.0%	0.0%	0.0%	0.0%	0.0%	1.00002
6	0.0%	0.0%	100.0%	0.0%	0.0%	0.0%	0.0%	1.00002
7	0.1%	0.0%	0.0%	0.0%	0.0%	0.0%	0.0%	2.10618
8	0.0%	0.0%	0.0%	0.0%	0.0%	0.0%	0.0%	2.09547
9	0.0%	0.0%	0.0%	0.0%	0.0%	0.0%	0.0%	2.07570
10	0.2%	0.0%	0.0%	0.0%	0.0%	0.0%	0.0%	2.09573
11	0.4%	0.0%	0.0%	0.0%	0.0%	0.0%	0.0%	2.07587
12	0.0%	0.3%	0.1%	0.2%	0.0%	0.0%	0.0%	2.09668
13	0.0%	0.0%	0.0%	0.0%	0.0%	0.0%	0.0%	2.06592
14	0.6%	0.1%	0.3%	0.0%	0.2%	0.0%	0.0%	2.09677
15	45.0%	0.0%	0.0%	0.0%	0.0%	0.0%	0.0%	2.06595
16	0.0%	49.1%	49.2%	0.4%	0.4%	0.4%	0.4%	2.06960
17	0.0%	45.5%	45.5%	1.5%	1.5%	1.4%	1.4%	2.40733
18	0.0%	52.4%	0.9%	45.3%	0.1%	0.6%	0.1%	2.08058
19	0.0%	52.9%	0.9%	0.6%	0.1%	44.9%	0.1%	2.07945
20	0.0%	0.9%	52.4%	0.1%	45.3%	0.1%	0.6%	2.08060
21	0.0%	0.9%	52.9%	0.1%	0.6%	0.1%	44.9%	2.07947
22	0.0%	0.9%	0.3%	0.1%	0.0%	0.3%	0.0%	2.67787
23	1.3%	0.3%	0.9%	0.0%	0.1%	0.0%	0.3%	2.67853

S1.4.6 IRC Step 10

1-center FNO analysis, $\varepsilon = 0.90000$		
MO	λ	Atoms
1	0.99999	C_1
2	0.99999	C_2
3	0.99999	C_5
4	0.99999	C_6
5	0.99999	C_{11}
6	0.99999	C_{12}
2-center FNO analysis, $\varepsilon = 0.90000$		
MO	λ	Atoms
7	0.97418	$C_1{-}C_2$
8	0.97496	$C_1{-}H_3$
9	0.98125	$C_1{-}C_5$
10	0.97477	$C_2{-}H_4$
11	0.98124	$C_2{-}C_6$
12	0.97153	$C_5{-}H_7$
13	0.98021	$C_5{-}H_9$
14	0.97153	$C_6{-}H_8$
15	0.98020	$C_6{-}H_{10}$
16	0.98265	$C_{11}{-}C_{12}$
17	0.90526	$C_{11}{-}C_{12}$
18	0.97658	$C_{11}{-}H_{13}$
19	0.97664	$C_{11}{-}H_{15}$
20	0.97660	$C_{12}{-}H_{14}$
21	0.97665	$C_{12}{-}H_{16}$
3-center FNO analysis, $\varepsilon = 0.90000$		
MO	λ	Atoms
22	0.93485	$C_1{-}C_2{-}C_5$
23	0.93485	$C_1{-}C_2{-}C_6$

Localization and atoms expanded by each FNO									
FNO	Loc_1	Loc_2	Loc_3	Loc_4	Loc_5	Loc_6	Loc_7	Loc_8	Loc_9
1	100.0%	0.0%	0.0%	0.0%	0.0%	0.0%	0.0%	0.0%	0.0%
2	0.0%	100.0%	0.0%	0.0%	0.0%	0.0%	0.0%	0.0%	0.0%
3	0.0%	0.0%	0.0%	0.0%	100.0%	0.0%	0.0%	0.0%	0.0%
4	0.0%	0.0%	0.0%	0.0%	0.0%	100.0%	0.0%	0.0%	0.0%
5	0.0%	0.0%	0.0%	0.0%	0.0%	0.0%	0.0%	0.0%	0.0%
6	0.0%	0.0%	0.0%	0.0%	0.0%	0.0%	0.0%	0.0%	0.0%
7	48.7%	48.7%	0.5%	0.5%	0.6%	0.6%	0.1%	0.1%	0.1%
8	51.8%	0.9%	45.7%	0.2%	1.0%	0.1%	0.1%	0.0%	0.2%
9	48.9%	0.5%	0.4%	0.1%	49.2%	0.1%	0.4%	0.0%	0.4%
10	0.9%	51.8%	0.2%	45.7%	0.1%	1.0%	0.0%	0.1%	0.0%
11	0.5%	48.9%	0.1%	0.4%	0.1%	49.2%	0.0%	0.4%	0.0%
12	0.8%	0.1%	0.1%	0.0%	52.9%	0.1%	44.3%	0.4%	0.6%
13	0.9%	0.1%	0.2%	0.0%	53.1%	0.0%	0.6%	0.0%	45.0%
14	0.1%	0.8%	0.0%	0.1%	0.1%	52.9%	0.4%	44.3%	0.0%
15	0.1%	0.9%	0.0%	0.2%	0.0%	53.1%	0.0%	0.6%	0.0%
16	0.0%	0.0%	0.0%	0.0%	0.0%	0.0%	0.0%	0.0%	0.0%
17	0.5%	0.5%	0.0%	0.0%	0.9%	0.9%	0.3%	0.3%	0.0%
18	0.0%	0.0%	0.0%	0.0%	0.2%	0.0%	0.2%	0.0%	0.0%
19	0.1%	0.0%	0.0%	0.0%	0.3%	0.0%	0.0%	0.0%	0.0%
20	0.0%	0.0%	0.0%	0.0%	0.0%	0.2%	0.0%	0.2%	0.0%
21	0.0%	0.1%	0.0%	0.0%	0.0%	0.3%	0.0%	0.0%	0.0%
22	45.9%	8.9%	1.6%	0.2%	38.7%	0.3%	1.1%	0.0%	1.3%
23	8.9%	45.9%	0.2%	1.6%	0.3%	38.7%	0.0%	1.1%	0.0%

Localization and atoms expanded by each FNO								
FNO	Loc_{10}	Loc_{11}	Loc_{12}	Loc_{13}	Loc_{14}	Loc_{15}	Loc_{16}	n_{eff}
1	0.0%	0.0%	0.0%	0.0%	0.0%	0.0%	0.0%	1.00002
2	0.0%	0.0%	0.0%	0.0%	0.0%	0.0%	0.0%	1.00002
3	0.0%	0.0%	0.0%	0.0%	0.0%	0.0%	0.0%	1.00002
4	0.0%	0.0%	0.0%	0.0%	0.0%	0.0%	0.0%	1.00002
5	0.0%	100.0%	0.0%	0.0%	0.0%	0.0%	0.0%	1.00002
6	0.0%	0.0%	100.0%	0.0%	0.0%	0.0%	0.0%	1.00002
7	0.1%	0.0%	0.0%	0.0%	0.0%	0.0%	0.0%	2.10686
8	0.0%	0.0%	0.0%	0.0%	0.0%	0.0%	0.0%	2.09492
9	0.0%	0.1%	0.0%	0.0%	0.0%	0.0%	0.0%	2.07683
10	0.2%	0.0%	0.0%	0.0%	0.0%	0.0%	0.0%	2.09575
11	0.4%	0.0%	0.1%	0.0%	0.0%	0.0%	0.0%	2.07687
12	0.0%	0.4%	0.1%	0.2%	0.0%	0.0%	0.0%	2.10185
13	0.0%	0.1%	0.0%	0.0%	0.0%	0.0%	0.0%	2.06691
14	0.6%	0.1%	0.4%	0.0%	0.2%	0.0%	0.0%	2.10186
15	45.0%	0.0%	0.1%	0.0%	0.0%	0.0%	0.0%	2.06699
16	0.0%	49.1%	49.1%	0.4%	0.4%	0.4%	0.4%	2.07096
17	0.0%	45.3%	45.3%	1.5%	1.5%	1.4%	1.4%	2.43391
18	0.0%	52.3%	0.9%	45.3%	0.1%	0.6%	0.1%	2.08570
19	0.0%	52.8%	0.9%	0.6%	0.1%	44.8%	0.1%	2.08225
20	0.0%	0.9%	52.3%	0.1%	45.3%	0.1%	0.6%	2.08562
21	0.0%	0.9%	52.8%	0.1%	0.6%	0.1%	44.8%	2.08223
22	0.0%	1.0%	0.4%	0.1%	0.0%	0.4%	0.0%	2.70968
23	1.3%	0.4%	1.0%	0.0%	0.1%	0.0%	0.4%	2.71011

S1.4.7 IRC Step 12

1-center FNO analysis, $\varepsilon = 0.90000$		
MO	λ	Atoms
1	0.99999	C_1
2	0.99999	C_2
3	0.99999	C_5
4	0.99999	C_6
5	0.99999	C_{11}
6	0.99999	C_{12}
2-center FNO analysis, $\varepsilon = 0.90000$		
MO	λ	Atoms
7	0.97404	$C_1{-}C_2$
8	0.97506	$C_1{-}H_3$
9	0.98090	$C_1{-}C_5$
10	0.97493	$C_2{-}H_4$
11	0.98091	$C_2{-}C_6$
12	0.97006	$C_5{-}H_7$
13	0.97985	$C_5{-}H_9$
14	0.97001	$C_6{-}H_8$
15	0.97984	$C_6{-}H_{10}$
16	0.98250	$C_{11}{-}C_{12}$
17	0.97570	$C_{11}{-}H_{13}$
18	0.97579	$C_{11}{-}H_{15}$
19	0.97555	$C_{12}{-}H_{14}$
20	0.97595	$C_{12}{-}H_{16}$
3-center FNO analysis, $\varepsilon = 0.90000$		
MO	λ	Atoms
21	0.93232	$C_1{-}C_2{-}C_5$
22	0.93228	$C_1{-}C_2{-}C_6$
23	0.90608	$C_5{-}C_{11}{-}C_{12}$

Localization and atoms expanded by each FNO									
FNO	Loc_1	Loc_2	Loc_3	Loc_4	Loc_5	Loc_6	Loc_7	Loc_8	Loc_9
1	100.0%	0.0%	0.0%	0.0%	0.0%	0.0%	0.0%	0.0%	0.0%
2	0.0%	100.0%	0.0%	0.0%	0.0%	0.0%	0.0%	0.0%	0.0%
3	0.0%	0.0%	0.0%	0.0%	100.0%	0.0%	0.0%	0.0%	0.0%
4	0.0%	0.0%	0.0%	0.0%	0.0%	100.0%	0.0%	0.0%	0.0%
5	0.0%	0.0%	0.0%	0.0%	0.0%	0.0%	0.0%	0.0%	0.0%
6	-0.0%	0.0%	0.0%	0.0%	0.0%	0.0%	0.0%	0.0%	0.0%
7	48.7%	48.7%	0.5%	0.5%	0.6%	0.6%	0.1%	0.1%	0.1%
8	51.8%	0.9%	45.7%	0.2%	1.0%	0.1%	0.1%	0.0%	0.2%
9	48.8%	0.5%	0.4%	0.1%	49.3%	0.1%	0.4%	0.0%	0.4%
10	0.9%	51.8%	0.2%	45.7%	0.1%	1.0%	0.0%	0.1%	0.0%
11	0.5%	48.8%	0.1%	0.4%	0.1%	49.3%	0.0%	0.4%	0.0%
12	0.8%	0.1%	0.1%	0.0%	52.8%	0.1%	44.2%	0.4%	0.6%
13	0.9%	0.1%	0.2%	0.0%	53.0%	0.0%	0.6%	0.0%	45.0%
14	0.1%	0.8%	0.0%	0.1%	0.1%	52.8%	0.4%	44.2%	0.0%
15	0.1%	0.9%	0.0%	0.2%	0.0%	53.0%	0.0%	0.6%	0.0%
16	0.0%	0.0%	0.0%	0.0%	0.0%	0.0%	0.0%	0.0%	0.0%
17	0.0%	0.0%	0.0%	0.0%	0.3%	0.0%	0.3%	0.0%	0.0%
18	0.2%	0.0%	0.0%	0.0%	0.4%	0.0%	0.0%	0.0%	0.0%
19	0.0%	0.0%	0.0%	0.0%	0.0%	0.3%	0.0%	0.3%	0.0%
20	0.0%	0.2%	0.0%	0.0%	0.0%	0.4%	0.0%	0.0%	0.0%
21	45.9%	9.6%	1.6%	0.2%	37.7%	0.4%	1.1%	0.0%	1.3%
22	9.6%	45.9%	0.2%	1.6%	0.4%	37.7%	0.0%	1.1%	0.0%
23	1.4%	0.9%	0.1%	0.0%	0.8%	1.2%	0.3%	0.4%	0.0%

Localization and atoms expanded by each FNO								
FNO	Loc_{10}	Loc_{11}	Loc_{12}	Loc_{13}	Loc_{14}	Loc_{15}	Loc_{16}	n_{eff}
1	0.0%	0.0%	0.0%	0.0%	0.0%	0.0%	0.0%	1.00002
2	0.0%	0.0%	0.0%	0.0%	0.0%	0.0%	0.0%	1.00002
3	0.0%	0.0%	0.0%	0.0%	0.0%	0.0%	0.0%	1.00002
4	0.0%	0.0%	0.0%	0.0%	0.0%	0.0%	0.0%	1.00002
5	0.0%	100.0%	0.0%	0.0%	0.0%	0.0%	0.0%	1.00002
6	0.0%	0.0% 1	00.0%	0.0%	0.0%	0.0%	0.0%	1.00002
7	0.1%	0.0%	0.0%	0.0%	0.0%	0.0%	0.0%	2.10745
8	0.0%	0.0%	0.0%	0.0%	0.0%	0.0%	0.0%	2.09447
9	0.0%	0.1%	0.0%	0.0%	0.0%	0.0%	0.0%	2.07828
10	0.2%	0.0%	0.0%	0.0%	0.0%	0.0%	0.0%	2.09503
11	0.4%	0.0%	0.1%	0.0%	0.0%	0.0%	0.0%	2.07824
12	0.0%	0.4%	0.1%	0.2%	0.0%	0.0%	0.0%	2.10813
13	0.0%	0.1%	0.0%	0.0%	0.0%	0.0%	0.0%	2.06873
14	0.6%	0.1%	0.4%	0.0%	0.2%	0.0%	0.0%	2.10834
15	45.0%	0.0%	0.1%	0.0%	0.0%	0.0%	0.0%	2.06879
16	0.0%	49.1%	49.1%	0.4%	0.4%	0.4%	0.4%	2.07160
17	0.0%	52.3%	0.9%	45.3%	0.1%	0.6%	0.1%	2.08957
18	0.0%	52.8%	0.9%	0.6%	0.1%	44.8%	0.1%	2.08592
19	0.0%	0.9%	52.3%	0.1%	45.3%	0.1%	0.6%	2.09022
20	0.0%	0.9%	52.8%	0.1%	0.6%	0.1%	44.8%	2.08522
21	0.0%	1.1%	0.5%	0.1%	0.0%	0.4%	0.0%	2.75375
22	1.3%	0.5%	1.1%	0.0%	0.1%	0.0%	0.4%	2.75447
23	0.0%	44.1%	45.2%	1.5%	1.5%	1.3%	1.4%	2.50159

S1.4.8 IRC Step 14

1-center FNO analysis, $\varepsilon = 0.90000$		
MO	λ	Atoms
1	0.99999	C_1
2	0.99999	C_2
3	0.99999	C_5
4	0.99999	C_6
5	0.99999	C_{11}
6	0.99999	C_{12}
2-center FNO analysis, $\varepsilon = 0.90000$		
MO	λ	Atoms
7	0.97392	$C_1{-}C_2$
8	0.97512	$C_1{-}H_3$
9	0.98043	$C_1{-}C_5$
10	0.97498	$C_2{-}H_4$
11	0.98048	$C_2{-}C_6$
12	0.96850	$C_5{-}H_7$
13	0.97933	$C_5{-}H_9$
14	0.96840	$C_6{-}H_8$
15	0.97941	$C_6{-}H_{10}$
16	0.98186	$C_{11}{-}C_{12}$
17	0.97456	$C_{11}{-}H_{13}$
18	0.97489	$C_{11}{-}H_{15}$
19	0.97434	$C_{12}{-}H_{14}$
20	0.97506	$C_{12}{-}H_{16}$
3-center FNO analysis, $\varepsilon = 0.90000$		
MO	λ	Atoms
21	0.92916	$C_1{-}C_2{-}C_5$
22	0.92905	$C_1{-}C_2{-}C_6$
23	0.90976	$C_5{-}C_{11}{-}C_{12}$

Localization and atoms expanded by each FNO									
FNO	Loc$_1$	Loc$_2$	Loc$_3$	Loc$_4$	Loc$_5$	Loc$_6$	Loc$_7$	Loc$_8$	Loc$_9$
1	100.0%	0.0%	0.0%	0.0%	0.0%	0.0%	0.0%	0.0%	0.0%
2	0.0%	100.0%	0.0%	0.0%	0.0%	0.0%	0.0%	0.0%	0.0%
3	0.0%	0.0%	0.0%	0.0%	100.0%	0.0%	0.0%	0.0%	0.0%
4	0.0%	0.0%	0.0%	0.0%	0.0%	100.0%	0.0%	0.0%	0.0%
5	-0.0%	0.0%	0.0%	0.0%	0.0%	0.0%	0.0%	0.0%	0.0%
6	-0.0%	0.0%	0.0%	0.0%	0.0%	0.0%	0.0%	0.0%	0.0%
7	48.7%	48.7%	0.5%	0.5%	0.6%	0.6%	0.1%	0.1%	0.1%
8	51.8%	0.9%	45.7%	0.2%	1.0%	0.1%	0.1%	0.0%	0.2%
9	48.6%	0.5%	0.4%	0.1%	49.5%	0.1%	0.4%	0.0%	0.4%
10	0.9%	51.8%	0.2%	45.7%	0.1%	1.0%	0.0%	0.1%	0.0%
11	0.5%	48.6%	0.1%	0.4%	0.1%	49.5%	0.0%	0.4%	0.0%
12	0.8%	0.1%	0.1%	0.0%	52.7%	0.1%	44.1%	0.4%	0.6%
13	0.9%	0.1%	0.2%	0.0%	52.9%	0.0%	0.6%	0.0%	45.0%
14	0.2%	0.8%	0.0%	0.1%	0.1%	52.7%	0.4%	44.1%	0.0%
15	0.1%	0.9%	0.0%	0.2%	0.0%	52.9%	0.0%	0.6%	0.0%
16	0.0%	0.0%	0.0%	0.0%	0.1%	0.1%	0.0%	0.0%	0.0%
17	0.0%	0.0%	0.0%	0.0%	0.4%	0.0%	0.3%	0.0%	0.0%
18	0.2%	0.0%	0.0%	0.0%	0.4%	0.0%	0.0%	0.0%	0.0%
19	0.0%	0.0%	0.0%	0.0%	0.0%	0.4%	0.0%	0.3%	0.0%
20	0.0%	0.2%	0.0%	0.0%	0.0%	0.4%	0.0%	0.0%	0.0%
21	45.9%	10.6%	1.6%	0.3%	36.4%	0.5%	1.0%	0.0%	1.2%
22	10.6%	45.9%	0.3%	1.6%	0.5%	36.4%	0.0%	1.0%	0.0%
23	0.5%	0.7%	0.0%	0.0%	3.8%	1.1%	0.6%	0.3%	0.1%

Localization and atoms expanded by each FNO								
FNO	Loc$_{10}$	Loc$_{11}$	Loc$_{12}$	Loc$_{13}$	Loc$_{14}$	Loc$_{15}$	Loc$_{16}$	n_{eff}
1	0.0%	0.0%	0.0%	0.0%	0.0%	0.0%	0.0%	1.00002
2	0.0%	0.0%	0.0%	0.0%	0.0%	0.0%	0.0%	1.00002
3	0.0%	0.0%	0.0%	0.0%	0.0%	0.0%	0.0%	1.00002
4	0.0%	0.0%	0.0%	0.0%	0.0%	0.0%	0.0%	1.00002
5	0.0%	100.0%	0.0%	0.0%	0.0%	0.0%	0.0%	1.00002
6	0.0%	0.0%	100.0%	0.0%	0.0%	0.0%	0.0%	1.00002
7	0.1%	0.0%	0.0%	0.0%	0.0%	0.0%	0.0%	2.10796
8	0.0%	0.0%	0.0%	0.0%	0.0%	0.0%	0.0%	2.09417
9	0.0%	0.1%	0.0%	0.0%	0.0%	0.0%	0.0%	2.08016
10	0.2%	0.0%	0.0%	0.0%	0.0%	0.0%	0.0%	2.09479
11	0.4%	0.0%	0.1%	0.0%	0.0%	0.0%	0.0%	2.07997
12	0.0%	0.5%	0.1%	0.2%	0.0%	0.0%	0.0%	2.11492
13	0.0%	0.1%	0.0%	0.0%	0.0%	0.1%	0.0%	2.07125
14	0.6%	0.1%	0.5%	0.0%	0.2%	0.0%	0.0%	2.11535
15	45.0%	0.0%	0.1%	0.0%	0.0%	0.0%	0.1%	2.07092
16	0.0%	49.1%	49.1%	0.4%	0.4%	0.4%	0.4%	2.07431
17	0.0%	52.2%	0.9%	45.3%	0.1%	0.6%	0.1%	2.09448
18	0.0%	52.7%	0.9%	0.6%	0.1%	44.8%	0.1%	2.08983
19	0.0%	0.9%	52.2%	0.1%	45.2%	0.1%	0.6%	2.09546
20	0.0%	0.9%	52.7%	0.1%	0.6%	0.1%	44.8%	2.08905
21	0.0%	1.3%	0.7%	0.1%	0.0%	0.4%	0.0%	2.81540
22	1.2%	0.7%	1.3%	0.0%	0.1%	0.0%	0.4%	2.81671
23	0.0%	45.3%	41.9%	1.6%	1.4%	1.5%	1.3%	2.61255

S1.4.9 IRC Step 16

1-center FNO analysis, $\varepsilon = 0.90000$		
MO	λ	Atoms
1	0.99999	C_1
2	0.99999	C_2
3	0.99999	C_5
4	0.99999	C_6
5	0.99999	C_{11}
6	0.99999	C_{12}
2-center FNO analysis, $\varepsilon = 0.90000$		
MO	λ	Atoms
7	0.97398	$C_1{-}C_2$
8	0.97516	$C_1{-}H_3$
9	0.97977	$C_1{-}C_5$
10	0.97504	$C_2{-}H_4$
11	0.97979	$C_2{-}C_6$
12	0.96705	$C_5{-}H_7$
13	0.97880	$C_5{-}H_9$
14	0.96685	$C_6{-}H_8$
15	0.97870	$C_6{-}H_{10}$
16	0.98118	$C_{11}{-}C_{12}$
17	0.97324	$C_{11}{-}H_{13}$
18	0.97437	$C_{11}{-}H_{15}$
19	0.97328	$C_{12}{-}H_{14}$
20	0.97421	$C_{12}{-}H_{16}$
3-center FNO analysis, $\varepsilon = 0.90000$		
MO	λ	Atoms
21	0.93232	$C_1{-}C_2{-}C_5$
22	0.93228	$C_1{-}C_2{-}C_6$
23	0.90608	$C_5{-}C_{11}{-}C_{12}$

Localization and atoms expanded by each FNO									
FNO	Loc_1	Loc_2	Loc_3	Loc_4	Loc_5	Loc_6	Loc_7	Loc_8	Loc_9
1	100.0%	0.0%	0.0%	0.0%	0.0%	0.0%	0.0%	0.0%	0.0%
2	0.0%	100.0%	0.0%	0.0%	0.0%	0.0%	0.0%	0.0%	0.0%
3	0.0%	0.0%	0.0%	0.0%	100.0%	0.0%	0.0%	0.0%	0.0%
4	0.0%	0.0%	0.0%	0.0%	0.0%	100.0%	0.0%	0.0%	0.0%
5	-0.0%	0.0%	0.0%	0.0%	0.0%	0.0%	0.0%	0.0%	0.0%
6	-0.0%	0.0%	0.0%	0.0%	0.0%	0.0%	0.0%	0.0%	0.0%
7	48.7%	48.7%	0.5%	0.5%	0.6%	0.6%	0.1%	0.1%	0.1%
8	51.8%	0.9%	45.7%	0.2%	1.0%	0.1%	0.1%	0.0%	0.2%
9	48.4%	0.5%	0.4%	0.1%	49.6%	0.1%	0.4%	0.0%	0.4%
10	0.9%	51.8%	0.2%	45.7%	0.1%	1.0%	0.0%	0.1%	0.0%
11	0.5%	48.4%	0.1%	0.4%	0.1%	49.6%	0.0%	0.4%	0.0%
12	0.8%	0.1%	0.1%	0.0%	52.6%	0.1%	44.1%	0.4%	0.6%
13	0.9%	0.1%	0.2%	0.0%	52.8%	0.0%	0.6%	0.0%	45.0%
14	0.2%	0.8%	0.0%	0.1%	0.1%	52.6%	0.4%	44.1%	0.0%
15	0.1%	0.9%	0.0%	0.2%	0.0%	52.8%	0.0%	0.6%	0.0%
16	0.0%	0.0%	0.0%	0.0%	0.1%	0.1%	0.0%	0.0%	0.0%
17	0.1%	0.0%	0.0%	0.0%	0.4%	0.0%	0.3%	0.0%	0.0%
18	0.2%	0.0%	0.0%	0.0%	0.5%	0.0%	0.0%	0.0%	0.0%
19	0.0%	0.1%	0.0%	0.0%	0.0%	0.4%	0.0%	0.3%	0.0%
20	0.0%	0.2%	0.0%	0.0%	0.0%	0.5%	0.0%	0.0%	0.0%
21	45.9%	12.4%	1.6%	0.3%	34.2%	0.6%	0.9%	0.0%	1.1%
22	12.5%	45.9%	0.3%	1.6%	0.6%	34.2%	0.0%	0.9%	0.0%
23	0.6%	0.9%	0.0%	0.1%	6.1%	1.4%	0.7%	0.3%	0.2%

Localization and atoms expanded by each FNO								
FNO	Loc_{10}	Loc_{11}	Loc_{12}	Loc_{13}	Loc_{14}	Loc_{15}	Loc_{16}	n_{eff}
1	0.0%	0.0%	0.0%	0.0%	0.0%	0.0%	0.0%	1.00002
2	0.0%	0.0%	0.0%	0.0%	0.0%	0.0%	0.0%	1.00002
3	0.0%	0.0%	0.0%	0.0%	0.0%	0.0%	0.0%	1.00002
4	0.0%	0.0%	0.0%	0.0%	0.0%	0.0%	0.0%	1.00002
5	0.0%	100.0%	0.0%	0.0%	0.0%	0.0%	0.0%	1.00002
6	0.0%	0.0%	100.0%	0.0%	0.0%	0.0%	0.0%	1.00002
7	0.1%	0.0%	0.0%	0.0%	0.0%	0.0%	0.0%	2.10769
8	0.0%	0.0%	0.0%	0.0%	0.0%	0.0%	0.0%	2.09399
9	0.0%	0.1%	0.0%	0.0%	0.0%	0.0%	0.0%	2.08283
10	0.2%	0.0%	0.0%	0.0%	0.0%	0.0%	0.0%	2.09453
11	0.4%	0.0%	0.1%	0.0%	0.0%	0.0%	0.0%	2.08271
12	0.0%	0.6%	0.1%	0.2%	0.0%	0.0%	0.0%	2.12140
13	0.0%	0.2%	0.0%	0.1%	0.0%	0.1%	0.0%	2.07392
14	0.6%	0.1%	0.6%	0.0%	0.2%	0.0%	0.0%	2.12231
15	45.0%	0.0%	0.2%	0.0%	0.1%	0.0%	0.1%	2.07435
16	0.0%	49.1%	49.1%	0.4%	0.4%	0.4%	0.4%	2.07717
17	0.0%	52.2%	0.9%	45.2%	0.1%	0.6%	0.1%	2.10000
18	0.0%	52.7%	0.9%	0.6%	0.1%	44.7%	0.1%	2.09194
19	0.0%	0.9%	52.2%	0.1%	45.2%	0.1%	0.6%	2.09980
20	0.0%	0.9%	52.7%	0.1%	0.6%	0.1%	44.7%	2.09263
21	0.0%	1.5%	0.8%	0.1%	0.1%	0.4%	0.1%	2.91058
22	1.1%	0.8%	1.5%	0.1%	0.1%	0.1%	0.4%	2.91173
23	0.0%	44.9%	39.3%	1.6%	1.3%	1.5%	1.2%	2.77181

S1.4.10 IRC Step 18

1-center FNO analysis, $\varepsilon = 0.90000$		
MO	λ	Atoms
1	0.99999	C_1
2	0.99999	C_2
3	0.99999	C_5
4	0.99999	C_6
5	0.99999	C_{11}
6	0.99999	C_{12}
2-center FNO analysis, $\varepsilon = 0.90000$		
MO	λ	Atoms
7	0.97432	$C_1{-}C_2$
8	0.97524	$C_1{-}H_3$
9	0.97875	$C_1{-}C_5$
10	0.97509	$C_2{-}H_4$
11	0.97881	$C_2{-}C_6$
12	0.96561	$C_5{-}H_7$
13	0.97808	$C_5{-}H_9$
14	0.96565	$C_6{-}H_8$
15	0.97814	$C_6{-}H_{10}$
16	0.98003	$C_{11}{-}C_{12}$
17	0.97247	$C_{11}{-}H_{13}$
18	0.97371	$C_{11}{-}H_{15}$
19	0.97251	$C_{12}{-}H_{14}$
20	0.97355	$C_{12}{-}H_{16}$
3-center FNO analysis, $\varepsilon = 0.90000$		
MO	λ	Atoms
21	0.91938	$C_1{-}C_2{-}C_5$
22	0.91939	$C_1{-}C_2{-}C_6$
4-center FNO analysis, $\varepsilon = 0.90000$		
MO	λ	Atoms
23	0.91286	$C_5{-}C_6{-}C_{11}{-}C_{12}$

Localization and atoms expanded by each FNO									
FNO	Loc_1	Loc_2	Loc_3	Loc_4	Loc_5	Loc_6	Loc_7	Loc_8	Loc_9
1	100.0%	0.0%	0.0%	0.0%	0.0%	0.0%	0.0%	0.0%	0.0%
2	0.0%	100.0%	0.0%	0.0%	0.0%	0.0%	0.0%	0.0%	0.0%
3	0.0%	0.0%	0.0%	0.0%	100.0%	0.0%	0.0%	0.0%	0.0%
4	0.0%	0.0%	0.0%	0.0%	0.0%	100.0%	0.0%	0.0%	0.0%
5	-0.0%	0.0%	0.0%	0.0%	0.0%	0.0%	0.0%	0.0%	0.0%
6	-0.0%	0.0%	0.0%	0.0%	0.0%	0.0%	0.0%	0.0%	0.0%
7	48.7%	48.7%	0.5%	0.5%	0.6%	0.6%	0.1%	0.1%	0.1%
8	51.9%	0.9%	45.7%	0.2%	1.0%	0.1%	0.1%	0.0%	0.2%
9	48.2%	0.5%	0.4%	0.1%	49.7%	0.1%	0.4%	0.0%	0.4%
10	0.9%	51.8%	0.2%	45.7%	0.1%	1.0%	0.0%	0.1%	0.0%
11	0.5%	48.2%	0.1%	0.4%	0.1%	49.7%	0.0%	0.4%	0.0%
12	0.8%	0.2%	0.1%	0.0%	52.5%	0.2%	44.1%	0.5%	0.7%
13	0.9%	0.1%	0.2%	0.0%	52.7%	0.0%	0.6%	0.0%	45.1%
14	0.2%	0.8%	0.0%	0.1%	0.2%	52.5%	0.5%	44.1%	0.0%
15	0.1%	0.9%	0.0%	0.2%	0.0%	52.7%	0.0%	0.6%	0.0%
16	0.0%	0.0%	0.0%	0.0%	0.1%	0.1%	0.0%	0.0%	0.0%
17	0.1%	0.0%	0.0%	0.0%	0.5%	0.0%	0.3%	0.0%	0.0%
18	0.2%	0.0%	0.0%	0.0%	0.5%	0.0%	0.0%	0.0%	0.0%
19	0.0%	0.1%	0.0%	0.0%	0.0%	0.5%	0.0%	0.3%	0.0%
20	0.0%	0.2%	0.0%	0.0%	0.0%	0.5%	0.0%	0.0%	0.0%
21	45.8%	15.7%	1.6%	0.4%	30.4%	0.9%	0.8%	0.0%	1.0%
22	15.7%	45.8%	0.4%	1.6%	0.9%	30.4%	0.0%	0.8%	0.0%
23	0.8%	0.8%	0.1%	0.1%	4.3%	4.3%	0.5%	0.5%	0.1%

Localization and atoms expanded by each FNO								
FNO	Loc_{10}	Loc_{11}	Loc_{12}	Loc_{13}	Loc_{14}	Loc_{15}	Loc_{16}	n_{eff}
1	0.0%	0.0%	0.0%	0.0%	0.0%	0.0%	0.0%	1.00002
2	0.0%	0.0%	0.0%	0.0%	0.0%	0.0%	0.0%	1.00002
3	0.0%	0.0%	0.0%	0.0%	0.0%	0.0%	0.0%	1.00002
4	0.0%	0.0%	0.0%	0.0%	0.0%	0.0%	0.0%	1.00002
5	0.0%	00.0%	0.0%	0.0%	0.0%	0.0%	0.0%	1.00002
6	0.0%	0.0%	100.0%	0.0%	0.0%	0.0%	0.0%	1.00002
7	0.1%	0.0%	0.0%	0.0%	0.0%	0.0%	0.0%	2.10627
8	0.0%	0.0%	0.0%	0.0%	0.0%	0.0%	0.0%	2.09358
9	0.0%	0.1%	0.0%	0.0%	0.0%	0.0%	0.0%	2.08698
10	0.2%	0.0%	0.0%	0.0%	0.0%	0.0%	0.0%	2.09425
11	0.4%	0.0%	0.1%	0.0%	0.0%	0.0%	0.0%	2.08673
12	0.0%	0.7%	0.2%	0.2%	0.0%	0.0%	0.0%	2.12809
13	0.0%	0.2%	0.0%	0.1%	0.0%	0.1%	0.0%	2.07753
14	0.7%	0.2%	0.7%	0.0%	0.2%	0.0%	0.0%	2.12793
15	45.1%	0.0%	0.2%	0.0%	0.1%	0.0%	0.1%	2.07726
16	0.0%	49.0%	49.0%	0.4%	0.4%	0.4%	0.4%	2.08202
17	0.0%	52.2%	0.9%	45.1%	0.1%	0.6%	0.1%	2.10282
18	0.0%	52.7%	0.9%	0.6%	0.1%	44.7%	0.1%	2.09463
19	0.0%	0.9%	52.2%	0.1%	45.1%	0.1%	0.6%	2.10265
20	0.0%	0.9%	52.7%	0.1%	0.6%	0.1%	44.7%	2.09534
21	0.0%	1.6%	1.1%	0.1%	0.1%	0.3%	0.1%	3.04943
22	1.0%	1.1%	1.6%	0.1%	0.1%	0.1%	0.3%	3.05044
23	0.1%	41.5%	41.5%	1.4%	1.4%	1.3%	1.3%	2.86184

S1.4.11 IRC Step 20

1-center FNO analysis, $\varepsilon = 0.90000$		
MO	λ	Atoms
1	0.99999	C_1
2	0.99999	C_2
3	0.99999	C_5
4	0.99999	C_6
5	0.99999	C_{11}
6	0.99999	C_{12}
2-center FNO analysis, $\varepsilon = 0.90000$		
MO	λ	Atoms
7	0.97505	C_1–C_2
8	0.97550	C_1–H_3
9	0.97727	C_1–C_5
10	0.97536	C_2–H_4
11	0.97722	C_2–C_6
12	0.96436	C_5–H_7
13	0.97729	C_5–H_9
14	0.96436	C_6–H_8
15	0.97733	C_6–H_{10}
16	0.97842	C_{11}–C_{12}
17	0.97193	C_{11}–C_{12}
18	0.97342	C_{11}–H_{13}
19	0.97191	C_{12}–H_{14}
20	0.97336	C_{12}–H_{16}
3-center FNO analysis, $\varepsilon = 0.90000$		
MO	λ	Atoms
21	0.93232	C_1–C_2–C_5
22	0.93228	C_1–C_2–C_6
23	0.90608	C_5–C_{11}–C_{12}
4-center FNO analysis, $\varepsilon = 0.90000$		
MO	λ	Atoms
23	0.90784	C_5–C_6–C_{11}–C_{12}

Localization and atoms expanded by each FNO									
FNO	Loc_1	Loc_2	Loc_3	Loc_4	Loc_5	Loc_6	Loc_7	Loc_8	Loc_9
1	100.0%	0.0%	0.0%	0.0%	0.0%	0.0%	0.0%	0.0%	0.0%
2	0.0%	100.0%	0.0%	0.0%	0.0%	0.0%	0.0%	0.0%	0.0%
3	0.0%	0.0%	0.0%	0.0%	100.0%	0.0%	0.0%	0.0%	0.0%
4	0.0%	0.0%	0.0%	0.0%	0.0%	100.0%	0.0%	0.0%	0.0%
5	-0.0%	0.0%	0.0%	0.0%	0.0%	0.0%	0.0%	0.0%	0.0%
6	-0.0%	0.0%	0.0%	0.0%	0.0%	0.0%	0.0%	0.0%	0.0%
7	48.7%	48.8%	0.5%	0.5%	0.6%	0.6%	0.1%	0.1%	0.1%
8	51.9%	0.9%	45.6%	0.2%	1.0%	0.1%	0.1%	0.0%	0.1%
9	48.1%	0.6%	0.4%	0.1%	49.6%	0.1%	0.4%	0.0%	0.5%
10	0.9%	51.9%	0.2%	45.6%	0.1%	1.0%	0.0%	0.1%	0.0%
11	0.6%	48.1%	0.1%	0.4%	0.1%	49.6%	0.0%	0.4%	0.0%
12	0.9%	0.2%	0.1%	0.0%	52.3%	0.2%	44.2%	0.5%	0.7%
13	0.9%	0.1%	0.1%	0.0%	52.5%	0.0%	0.6%	0.0%	45.2%
14	0.2%	0.9%	0.0%	0.1%	0.2%	52.3%	0.5%	44.2%	0.0%
15	0.1%	0.8%	0.0%	0.2%	0.0%	52.5%	0.0%	0.6%	0.0%
16	0.0%	0.0%	0.0%	0.0%	0.1%	0.1%	0.0%	0.0%	0.0%
17	0.1%	0.0%	0.0%	0.0%	0.6%	0.0%	0.2%	0.0%	0.0%
18	0.1%	0.0%	0.0%	0.0%	0.6%	0.0%	0.0%	0.0%	0.1%
19	0.0%	0.1%	0.0%	0.0%	0.0%	0.6%	0.0%	0.2%	0.0%
20	-0.0%	0.2%	0.0%	0.0%	0.0%	0.6%	0.0%	0.0%	0.0%
21	45.6%	21.4%	1.6%	0.7%	24.4%	1.4%	0.7%	0.1%	0.7%
22	21.4%	45.6%	0.7%	1.6%	1.4%	24.4%	0.1%	0.7%	0.0%
23	0.9%	0.9%	0.1%	0.1%	6.8%	6.8%	0.6%	0.6%	0.2%

Localization and atoms expanded by each FNO								
FNO	Loc_{10}	Loc_{11}	Loc_{12}	Loc_{13}	Loc_{14}	Loc_{15}	Loc_{16}	n_{eff}
1	0.0%	0.0%	0.0%	0.0%	0.0%	0.0%	0.0%	1.00002
2	0.0%	0.0%	0.0%	0.0%	0.0%	0.0%	0.0%	1.00002
3	0.0%	0.0%	0.0%	0.0%	0.0%	0.0%	0.0%	1.00002
4	0.0%	0.0%	0.0%	0.0%	0.0%	0.0%	0.0%	1.00002
5	0.0%	100.0%	0.0%	0.0%	0.0%	0.0%	0.0%	1.00002
6	0.0%	0.0%	100.0%	0.0%	0.0%	0.0%	0.0%	1.00002
7	0.1%	0.0%	0.0%	0.0%	0.0%	0.0%	0.0%	2.10313
8	0.0%	0.0%	0.0%	0.0%	0.0%	0.0%	0.0%	2.09229
9	0.0%	0.2%	0.0%	0.0%	0.0%	0.0%	0.0%	2.09322
10	0.2%	0.0%	0.0%	0.0%	0.0%	0.0%	0.0%	2.09292
11	0.5%	0.0%	0.2%	0.0%	0.0%	0.0%	0.0%	2.09339
12	0.0%	0.7%	0.2%	0.2%	0.0%	0.0%	0.0%	2.13460
13	0.0%	0.3%	0.0%	0.1%	0.0%	0.1%	0.0%	2.08174
14	0.7%	0.2%	0.7%	0.0%	0.2%	0.0%	0.0%	2.13458
15	45.2%	0.0%	0.3%	0.0%	0.1%	0.0%	0.1%	2.08156
16	0.0%	48.9%	48.9%	0.4%	0.4%	0.4%	0.4%	2.08885
17	0.0%	52.2%	0.9%	45.0%	0.1%	0.6%	0.1%	2.10471
18	0.0%	52.6%	0.9%	0.6%	0.1%	44.7%	0.1%	2.09599
19	0.0%	0.9%	52.2%	0.1%	45.0%	0.1%	0.6%	2.10481
20	0.1%	0.9%	52.6%	0.1%	0.6%	0.1%	44.7%	2.09626
21	0.0%	1.6%	1.3%	0.1%	0.1%	0.3%	0.1%	3.17910
22	0.7%	1.3%	1.6%	0.1%	0.1%	0.1%	0.3%	3.18005
23	0.2%	38.8%	38.8%	1.3%	1.3%	1.3%	1.3%	3.21744

S1.4.12 IRC Step 22

1-center FNO analysis, $\varepsilon = 0.90000$		
MO	λ	Atoms
1	0.99999	C_1
2	0.99999	C_2
3	0.99999	C_5
4	0.99999	C_6
5	0.99999	C_{11}
6	0.99999	C_{12}
2-center FNO analysis, $\varepsilon = 0.90000$		
MO	λ	Atoms
7	0.97686	$C_1{-}C_2$
8	0.97677	$C_1{-}H_3$
9	0.97345	$C_1{-}C_5$
10	0.97661	$C_2{-}H_4$
11	0.97333	$C_2{-}C_6$
12	0.96285	$C_5{-}H_7$
13	0.97544	$C_5{-}H_9$
14	0.96297	$C_6{-}H_8$
15	0.97541	$C_6{-}H_{10}$
16	0.97323	$C_{11}{-}C_{12}$
17	0.97117	$C_{11}{-}H_{13}$
18	0.97220	$C_{11}{-}H_{15}$
19	0.97119	$C_{12}{-}H_{14}$
20	0.97224	$C_{12}{-}H_{16}$
3-center FNO analysis, $\varepsilon = 0.90000$		
MO	λ	Atoms
21	0.91509	$C_1{-}C_2{-}C_5$
22	0.91503	$C_1{-}C_2{-}C_6$
23	0.90097	$C_1{-}C_5{-}C_{11}$

Localization and atoms expanded by each FNO									
FNO	Loc_1	Loc_2	Loc_3	Loc_4	Loc_5	Loc_6	Loc_7	Loc_8	Loc_9
1	100.0%	0.0%	0.0%	0.0%	0.0%	0.0%	0.0%	0.0%	0.0%
2	0.0%	100.0%	0.0%	0.0%	0.0%	0.0%	0.0%	0.0%	0.0%
3	0.0%	0.0%	0.0%	0.0%	100.0%	0.0%	0.0%	0.0%	0.0%
4	0.0%	0.0%	0.0%	0.0%	0.0%	100.0%	0.0%	0.0%	0.0%
5	-0.0%	0.0%	0.0%	0.0%	0.0%	0.0%	0.0%	0.0%	0.0%
6	-0.0%	0.0%	0.0%	0.0%	0.0%	0.0%	0.0%	0.0%	0.0%
7	48.8%	48.8%	0.5%	0.5%	0.6%	0.6%	0.1%	0.1%	0.1%
8	52.1%	0.9%	45.5%	0.2%	0.9%	0.1%	0.1%	0.0%	0.1%
9	48.3%	0.6%	0.5%	0.1%	49.0%	0.1%	0.5%	0.0%	0.5%
10	0.9%	52.1%	0.2%	45.5%	0.1%	0.9%	0.0%	0.1%	0.0%
11	0.6%	48.3%	0.1%	0.5%	0.1%	49.0%	0.0%	0.5%	0.0%
12	1.0%	0.2%	0.1%	0.0%	51.6%	0.2%	44.7%	0.4%	0.7%
13	0.9%	0.1%	0.1%	0.0%	51.9%	0.0%	0.7%	0.0%	45.6%
14	0.2%	1.0%	0.0%	0.1%	0.2%	51.6%	0.4%	44.7%	0.0%
15	0.1%	0.9%	0.0%	0.1%	0.0%	51.9%	0.0%	0.7%	0.0%
16	0.1%	0.0%	0.0%	0.0%	0.3%	0.3%	0.0%	0.0%	0.0%
17	0.1%	0.0%	0.0%	0.0%	0.6%	0.1%	0.1%	0.0%	0.0%
18	0.2%	0.0%	0.0%	0.0%	0.6%	0.1%	0.0%	0.0%	0.1%
19	0.0%	0.1%	0.0%	0.0%	0.1%	0.6%	0.0%	0.1%	0.0%
20	0.0%	0.1%	0.0%	0.0%	0.1%	0.6%	0.0%	0.0%	0.0%
21	45.6%	34.2%	1.6%	1.2%	11.7%	2.2%	0.3%	0.2%	0.3%
22	34.2%	45.6%	1.2%	1.6%	2.2%	11.7%	0.2%	0.3%	0.0%
23	14.8%	0.9%	0.4%	0.0%	43.5%	0.9%	1.5%	0.0%	1.4%

Localization and atoms expanded by each FNO								
FNO	Loc_{10}	Loc_{11}	Loc_{12}	Loc_{13}	Loc_{14}	Loc_{15}	Loc_{16}	n_{eff}
1	0.0%	0.0%	0.0%	0.0%	0.0%	0.0%	0.0%	1.00002
2	0.0%	0.0%	0.0%	0.0%	0.0%	0.0%	0.0%	1.00002
3	0.0%	0.0%	0.0%	0.0%	0.0%	0.0%	0.0%	1.00002
4	0.0%	0.0%	0.0%	0.0%	0.0%	0.0%	0.0%	1.00002
5	0.0%	100.0%	0.0%	0.0%	0.0%	0.0%	0.0%	1.00002
6	0.0%	0.0%	100.0%	0.0%	0.0%	0.0%	0.0%	1.00002
7	0.1%	0.0%	0.0%	0.0%	0.0%	0.0%	0.0%	2.09542
8	0.0%	0.0%	0.0%	0.0%	0.0%	0.0%	0.0%	2.08597
9	0.0%	0.2%	0.0%	0.0%	0.0%	0.0%	0.0%	2.10990
10	0.2%	0.0%	0.0%	0.0%	0.0%	0.0%	0.0%	2.08668
11	0.5%	0.0%	0.2%	0.0%	0.0%	0.0%	0.0%	2.11044
12	0.0%	0.7%	0.1%	0.1%	0.0%	0.0%	0.0%	2.14526
13	0.0%	0.4%	0.0%	0.0%	0.0%	0.1%	0.0%	2.09252
14	0.7%	0.1%	0.7%	0.0%	0.1%	0.0%	0.0%	2.14462
15	45.6%	0.0%	0.4%	0.0%	0.0%	0.0%	0.1%	2.09266
16	0.0%	48.7%	48.7%	0.5%	0.5%	0.5%	0.5%	2.11106
17	0.0%	51.8%	1.0%	45.3%	0.2%	0.7%	0.1%	2.11007
18	0.0%	52.1%	0.9%	0.7%	0.1%	45.1%	0.2%	2.10454
19	0.0%	1.0%	51.8%	0.2%	45.3%	0.1%	0.7%	2.10997
20	0.1%	0.9%	52.1%	0.1%	0.7%	0.2%	45.1%	2.10434
21	0.0%	1.0%	1.3%	0.0%	0.1%	0.2%	0.1%	2.93923
22	0.3%	1.3%	1.0%	0.1%	0.0%	0.1%	0.2%	2.93971
23	0.1%	32.1%	1.7%	1.1%	0.1%	1.3%	0.1%	3.17282

S1.4.13 IRC Step 24

1-center FNO analysis, $\varepsilon = 0.90000$		
MO	λ	Atoms
1	0.99999	C_1
2	0.99999	C_2
3	0.99999	C_5
4	0.99999	C_6
5	0.99999	C_{11}
6	0.99999	C_{12}
2-center FNO analysis, $\varepsilon = 0.90000$		
MO	λ	Atoms
7	0.97844	C_1-C_2
8	0.97861	C_1-H_3
9	0.96855	C_1-C_5
10	0.97843	C_2-H_4
11	0.96851	C_2-C_6
12	0.95951	C_5-H_7
13	0.97217	C_5-H_9
14	0.90868	C_5-H_{11}
15	0.95962	C_6-H_8
16	0.97219	C_6-C_{10}
17	0.90862	C_6-C_{12}
18	0.96506	$C_{11}-C_{12}$
19	0.96967	$C_{11}-H_{13}$
20	0.97061	$C_{11}-H_{15}$
21	0.96953	$C_{12}-H_{14}$
22	0.97043	$C_{12}-H_{16}$
3-center FNO analysis, $\varepsilon = 0.90000$		
MO	λ	Atoms
23	0.90649	$C_1-C_2-H_3$

Localization and atoms expanded by each FNO									
FNO	Loc_1	Loc_2	Loc_3	Loc_4	Loc_5	Loc_6	Loc_7	Loc_8	Loc_9
1	100.0%	0.0%	0.0%	0.0%	0.0%	0.0%	0.0%	0.0%	0.0%
2	0.0%	100.0%	0.0%	0.0%	0.0%	0.0%	0.0%	0.0%	0.0%
3	0.0%	0.0%	0.0%	0.0%	100.0%	0.0%	0.0%	0.0%	0.0%
4	-0.0%	0.0%	0.0%	0.0%	0.0%	100.0%	0.0%	0.0%	0.0%
5	0.0%	0.0%	0.0%	0.0%	0.0%	0.0%	0.0%	0.0%	0.0%
6	0.0%	0.0%	0.0%	0.0%	0.0%	0.0%	0.0%	0.0%	0.0%
7	48.9%	48.9%	0.4%	0.4%	0.5%	0.5%	0.0%	0.0%	0.1%
8	52.5%	0.9%	45.3%	0.1%	0.8%	0.1%	0.0%	0.0%	0.2%
9	49.0%	0.7%	0.5%	0.1%	47.9%	0.2%	0.6%	0.0%	0.6%
10	0.9%	52.5%	0.1%	45.3%	0.1%	0.8%	0.0%	0.0%	0.0%
11	0.7%	49.0%	0.1%	0.5%	0.2%	47.9%	0.0%	0.6%	0.0%
12	1.2%	0.3%	0.1%	0.0%	50.4%	0.2%	45.5%	0.4%	0.8%
13	0.9%	0.1%	0.1%	0.0%	50.9%	0.0%	0.8%	0.0%	46.3%
14	2.0%	0.7%	0.0%	0.1%	45.7%	0.4%	1.1%	0.1%	1.0%
15	0.3%	1.1%	0.0%	0.1%	0.2%	50.4%	0.4%	45.5%	0.0%
16	0.1%	0.9%	0.0%	0.1%	0.0%	50.9%	0.0%	0.8%	0.0%
17	0.7%	2.0%	0.1%	0.0%	0.4%	45.7%	0.1%	1.1%	0.0%
18	0.1%	0.1%	0.0%	0.0%	0.4%	0.4%	0.0%	0.0%	0.0%
19	0.1%	0.0%	0.0%	0.0%	0.7%	0.0%	0.1%	0.0%	0.0%
20	0.2%	0.0%	0.0%	0.0%	0.7%	0.0%	0.1%	0.0%	0.1%
21	0.1%	0.1%	0.0%	0.0%	0.0%	0.7%	0.0%	0.1%	0.0%
22	0.0%	0.1%	0.0%	0.0%	0.0%	0.7%	0.0%	0.1%	0.0%
23	44.6%	44.4%	1.7%	1.7%	2.4%	2.4%	0.3%	0.3%	0.0%

Localization and atoms expanded by each FNO								
FNO	Loc_{10}	Loc_{11}	Loc_{12}	Loc_{13}	Loc_{14}	Loc_{15}	Loc_{16}	n_{eff}
1	0.0%	0.0%	0.0%	0.0%	0.0%	0.0%	0.0%	1.00002
2	0.0%	0.0%	0.0%	0.0%	0.0%	0.0%	0.0%	1.00002
3	0.0%	0.0%	0.0%	0.0%	0.0%	0.0%	0.0%	1.00002
4	0.0%	0.0%	0.0%	0.0%	0.0%	0.0%	0.0%	1.00002
5	0.0%	100.0%	0.0%	0.0%	0.0%	0.0%	0.0%	1.00001
6	0.0%	0.0%	100.0%	0.0%	0.0%	0.0%	0.0%	1.00001
7	0.1%	0.0%	0.0%	0.0%	0.0%	0.0%	0.0%	2.08871
8	0.0%	0.0%	0.0%	0.0%	0.0%	0.0%	0.0%	2.07660
9	0.0%	0.4%	0.0%	0.0%	0.0%	0.0%	0.0%	2.13094
10	0.2%	0.0%	0.0%	0.0%	0.0%	0.0%	0.0%	2.07741
11	0.6%	0.0%	0.4%	0.0%	0.0%	0.0%	0.0%	2.13114
12	0.0%	0.7%	0.1%	0.1%	0.0%	0.1%	0.0%	2.16529
13	0.0%	0.6%	0.0%	0.0%	0.0%	0.1%	0.0%	2.11052
14	0.0%	45.2%	1.4%	1.1%	0.0%	1.1%	0.0%	2.41551
15	0.8%	0.1%	0.7%	0.0%	0.1%	0.0%	0.1%	2.16479
16	46.3%	0.0%	0.6%	0.0%	0.0%	0.0%	0.1%	2.11045
17	1.0%	1.4%	45.2%	0.0%	1.1%	0.0%	1.1%	2.41585
18	0.0%	48.3%	48.3%	0.6%	0.6%	0.6%	0.6%	2.14662
19	0.0%	50.8%	0.9%	46.1%	0.2%	0.8%	0.1%	2.12112
20	0.0%	51.1%	0.9%	0.8%	0.1%	46.0%	0.2%	2.11637
21	0.0%	0.9%	50.8%	0.2%	46.1%	0.1%	0.8%	2.12172
22	0.1%	0.9%	51.0%	0.1%	0.8%	0.2%	46.0%	2.11714
23	0.0%	0.9%	0.9%	0.1%	0.1%	0.1%	0.1%	2.51213

S1.4.14 IRC Step 26

1-center FNO analysis, $\varepsilon = 0.90000$		
MO	λ	Atoms
1	0.99999	C_1
2	0.99999	C_2
3	0.99999	C_5
4	0.99999	C_6
5	0.99999	C_{11}
6	0.99999	C_{12}
2-center FNO analysis, $\varepsilon = 0.90000$		
MO	λ	Atoms
7	0.97686	C_1-C_2
8	0.97677	C_1-C_2
9	0.97345	C_1-H_3
10	0.97661	C_1-C_5
11	0.97333	C_2-H_4
12	0.97333	C_2-C_6
13	0.96285	C_5-H_7
14	0.97544	C_5-H_9
15	0.96297	C_5-C_{11}
16	0.97541	C_6-H_8
17	0.97323	C_6-H_{10}
18	0.97117	C_6-C_{12}
19	0.97220	$C_{11}-C_{12}$
20	0.97119	$C_{11}-H_{13}$
21	0.97224	$C_{11}-H_{15}$
22	0.97119	$C_{12}-H_{14}$
23	0.97224	$C_{12}-H_{16}$

Localization and atoms expanded by each FNO									
FNO	Loc_1	Loc_2	Loc_3	Loc_4	Loc_5	Loc_6	Loc_7	Loc_8	Loc_9
1	100.0%	0.0%	0.0%	0.0%	0.0%	0.0%	0.0%	0.0%	0.0%
2	0.0%	100.0%	0.0%	0.0%	0.0%	0.0%	0.0%	0.0%	0.0%
3	0.0%	0.0%	0.0%	0.0%	100.0%	0.0%	0.0%	0.0%	0.0%
4	0.0%	0.0%	0.0%	0.0%	0.0%	100.0%	0.0%	0.0%	0.0%
5	0.0%	0.0%	0.0%	0.0%	0.0%	0.0%	0.0%	0.0%	0.0%
6	-0.0%	0.0%	0.0%	0.0%	0.0%	0.0%	0.0%	0.0%	0.0%
7	49.0%	49.0%	0.4%	0.4%	0.5%	0.5%	0.0%	0.0%	0.1%
8	45.4%	45.3%	1.7%	1.7%	1.8%	1.9%	0.4%	0.4%	0.0%
9	52.8%	0.8%	45.2%	0.1%	0.7%	0.1%	0.0%	0.0%	0.2%
10	49.8%	0.8%	0.5%	0.1%	46.5%	0.2%	0.6%	0.0%	0.7%
11	0.8%	52.8%	0.1%	45.2%	0.1%	0.7%	0.0%	0.0%	0.0%
12	0.8%	49.8%	0.1%	0.5%	0.2%	46.5%	0.0%	0.6%	0.0%
13	1.3%	0.4%	0.0%	0.0%	49.3%	0.2%	46.3%	0.4%	0.9%
14	1.0%	0.1%	0.1%	0.0%	49.8%	0.0%	0.9%	0.0%	47.0%
15	1.3%	0.4%	0.0%	0.0%	47.1%	0.2%	0.9%	0.0%	0.8%
16	0.4%	1.3%	0.0%	0.0%	0.2%	49.3%	0.4%	46.3%	0.0%
17	0.1%	1.0%	0.0%	0.1%	0.0%	49.7%	0.0%	0.9%	0.0%
18	0.4%	1.3%	0.0%	0.0%	0.2%	47.1%	0.0%	0.9%	0.0%
19	0.1%	0.1%	0.0%	0.0%	0.6%	0.6%	0.0%	0.0%	0.1%
20	0.1%	0.0%	0.0%	0.0%	0.9%	0.0%	0.1%	0.0%	0.1%
21	0.2%	0.0%	0.0%	0.0%	0.9%	0.0%	0.1%	0.0%	0.1%
22	0.1%	0.1%	0.0%	0.0%	0.0%	0.9%	0.0%	0.1%	0.0%
23	0.0%	0.2%	0.0%	0.0%	0.0%	0.9%	0.0%	0.1%	0.0%

Localization and atoms expanded by each FNO								
FNO	Loc_{10}	Loc_{11}	Loc_{12}	Loc_{13}	Loc_{14}	Loc_{15}	Loc_{16}	n_{eff}
1	0.0%	0.0%	0.0%	0.0%	0.0%	0.0%	0.0%	1.00002
2	0.0%	0.0%	0.0%	0.0%	0.0%	0.0%	0.0%	1.00002
3	0.0%	0.0%	0.0%	0.0%	0.0%	0.0%	0.0%	1.00002
4	0.0%	0.0%	0.0%	0.0%	0.0%	0.0%	0.0%	1.00002
5	0.0%	100.0%	0.0%	0.0%	0.0%	0.0%	0.0%	1.00001
6	0.0%	0.0%	100.0%	0.0%	0.0%	0.0%	0.0%	1.00001
7	0.1%	0.0%	0.0%	0.0%	0.0%	0.0%	0.0%	2.08476
8	0.0%	0.5%	0.5%	0.1%	0.1%	0.1%	0.1%	2.42436
9	0.0%	0.0%	0.0%	0.0%	0.0%	0.0%	0.0%	2.06835
10	0.0%	0.6%	0.1%	0.1%	0.0%	0.0%	0.0%	2.15221
11	0.2%	0.0%	0.0%	0.0%	0.0%	0.0%	0.0%	2.06867
12	0.7%	0.1%	0.6%	0.0%	0.1%	0.0%	0.0%	2.15244
13	0.0%	0.9%	0.1%	0.1%	0.0%	0.1%	0.0%	2.18689
14	0.0%	0.8%	0.1%	0.1%	0.0%	0.1%	0.0%	2.13436
15	0.0%	46.5%	0.9%	0.9%	0.0%	0.8%	0.0%	2.27793
16	0.9%	0.1%	0.9%	0.0%	0.1%	0.0%	0.1%	2.18688
17	47.0%	0.1%	0.8%	0.0%	0.1%	0.0%	0.1%	2.13456
18	0.8%	0.9%	46.5%	0.0%	0.9%	0.0%	0.8%	2.27842
19	0.1%	47.8%	47.8%	0.7%	0.7%	0.7%	0.7%	2.18925
20	0.0%	49.7%	0.9%	47.0%	0.2%	0.9%	0.0%	2.13849
21	0.0%	49.8%	0.9%	0.9%	0.0%	46.8%	0.2%	2.13661
22	0.1%	0.9%	49.7%	0.2%	47.0%	0.0%	0.9%	2.13807
23	0.1%	0.9%	49.8%	0.0%	0.9%	0.2%	46.8%	2.13741

S1.4.15 IRC Step 28

1-center FNO analysis, $\varepsilon = 0.90000$		
MO	λ	Atoms
1	0.99999	C_1
2	0.99999	C_2
3	0.99999	C_5
4	0.99999	C_6
5	0.99999	C_{11}
6	0.99999	C_{12}
**2-center FNO analysis, $\varepsilon = 0.90000$**		
MO	λ	Atoms
7	0.97950	C_1-C_2
8	0.91012	C_1-C_2
9	0.97998	C_1-H_3
10	0.96117	C_1-C_5
11	0.97988	C_2-H_4
12	0.96110	C_2-C_6
13	0.95460	C_5-H_7
14	0.96391	C_5-H_9
15	0.94434	C_5-C_{11}
16	0.95464	C_6-H_8
17	0.96387	C_6-H_{10}
18	0.94448	C_6-C_{12}
19	0.95145	$C_{11}-C_{12}$
20	0.96389	$C_{11}-H_{13}$
21	0.96251	$C_{11}-H_{15}$
22	0.96400	$C_{12}-H_{14}$
23	0.96246	$C_{12}-H_{16}$

Localization and atoms expanded by each FNO									
FNO	Loc_1	Loc_2	Loc_3	Loc_4	Loc_5	Loc_6	Loc_7	Loc_8	Loc_9
1	100.0%	0.0%	0.0%	0.0%	0.0%	0.0%	0.0%	0.0%	0.0%
2	0.0%	100.0%	0.0%	0.0%	0.0%	0.0%	0.0%	0.0%	0.0%
3	0.0%	0.0%	0.0%	0.0%	100.0%	0.0%	0.0%	0.0%	0.0%
4	0.0%	0.0%	0.0%	0.0%	0.0%	100.0%	0.0%	0.0%	0.0%
5	0.0%	0.0%	0.0%	0.0%	0.0%	0.0%	0.0%	0.0%	0.0%
6	-0.0%	0.0%	0.0%	0.0%	0.0%	0.0%	0.0%	0.0%	0.0%
7	49.0%	49.0%	0.4%	0.4%	0.5%	0.5%	0.0%	0.0%	0.1%
8	45.5%	45.5%	1.7%	1.7%	1.7%	1.7%	0.4%	0.4%	0.0%
9	52.7%	0.8%	45.3%	0.1%	0.7%	0.1%	0.0%	0.0%	0.2%
10	50.2%	0.8%	0.5%	0.1%	45.9%	0.2%	0.7%	0.0%	0.7%
11	0.9%	52.7%	0.1%	45.3%	0.1%	0.7%	0.0%	0.0%	0.0%
12	0.8%	50.2%	0.1%	0.5%	0.2%	45.9%	0.0%	0.7%	0.0%
13	1.4%	0.4%	0.0%	0.0%	48.7%	0.1%	46.7%	0.2%	1.0%
14	1.0%	0.1%	0.2%	0.0%	49.1%	0.0%	0.9%	0.0%	47.3%
15	1.1%	0.3%	0.1%	0.0%	47.5%	0.2%	0.8%	0.0%	0.7%
16	0.4%	1.4%	0.0%	0.0%	0.1%	48.7%	0.2%	46.7%	0.0%
17	0.1%	1.0%	0.0%	0.2%	0.0%	49.1%	0.0%	0.9%	0.0%
18	0.3%	1.1%	0.0%	0.1%	0.2%	47.5%	0.0%	0.8%	0.0%
19	0.1%	0.1%	0.0%	0.0%	0.7%	0.7%	0.0%	0.0%	0.1%
20	0.2%	0.0%	0.0%	0.0%	1.0%	0.0%	0.2%	0.0%	0.1%
21	0.2%	0.0%	0.0%	0.0%	1.0%	0.0%	0.1%	0.0%	0.1%
22	0.1%	0.1%	0.0%	0.0%	0.0%	1.0%	0.0%	0.2%	0.0%
23	0.0%	0.2%	0.0%	0.0%	0.0%	1.0%	0.0%	0.1%	0.0%

Localization and atoms expanded by each FNO								
FNO	Loc_{10}	Loc_{11}	Loc_{12}	Loc_{13}	Loc_{14}	Loc_{15}	Loc_{16}	n_{eff}
1	0.0%	0.0%	0.0%	0.0%	0.0%	0.0%	0.0%	1.00002
2	0.0%	0.0%	0.0%	0.0%	0.0%	0.0%	0.0%	1.00002
3	0.0%	0.0%	0.0%	0.0%	0.0%	0.0%	0.0%	1.00002
4	0.0%	0.0%	0.0%	0.0%	0.0%	0.0%	0.0%	1.00002
5	0.0%	100.0%	0.0%	0.0%	0.0%	0.0%	0.0%	1.00002
6	0.0%	0.0%	100.0%	0.0%	0.0%	0.0%	0.0%	1.00002
7	0.1%	0.1%	0.1%	0.0%	0.0%	0.0%	0.0%	2.08427
8	0.0%	0.4%	0.4%	0.1%	0.1%	0.1%	0.1%	2.40748
9	0.0%	0.0%	0.0%	0.0%	0.0%	0.0%	0.0%	2.07010
10	0.0%	0.7%	0.1%	0.1%	0.0%	0.0%	0.0%	2.15944
11	0.2%	0.0%	0.0%	0.0%	0.0%	0.0%	0.0%	2.07055
12	0.7%	0.1%	0.7%	0.0%	0.1%	0.0%	0.0%	2.15977
13	0.0%	1.0%	0.1%	0.2%	0.0%	0.1%	0.0%	2.19186
14	0.0%	1.0%	0.1%	0.1%	0.0%	0.2%	0.0%	2.15042
15	0.0%	46.9%	0.7%	0.8%	0.0%	0.8%	0.0%	2.24050
16	1.0%	0.1%	1.0%	0.0%	0.1%	0.0%	0.1%	2.19168
17	47.3%	0.1%	1.0%	0.0%	0.1%	0.0%	0.2%	2.15060
18	0.7%	0.7%	46.9%	0.0%	0.8%	0.0%	0.8%	2.23983
19	0.1%	47.6%	47.6%	0.7%	0.7%	0.7%	0.7%	2.20778
20	0.0%	48.9%	0.9%	47.5%	0.2%	0.9%	0.0%	2.15091
21	0.0%	48.9%	0.9%	0.9%	0.0%	47.3%	0.3%	2.15688
22	0.1%	0.9%	48.9%	0.2%	47.5%	0.0%	0.9%	2.15041
23	0.1%	0.9%	48.9%	0.0%	0.9%	0.3%	47.3%	2.15713

S1.5 The F^{-}+CH_3F → FCH_3+ F^{-} reaction

Due to the symmetry of the fluorine self-substitution reaction only the forward reaction profile has been analyzed. The geometry of the products is equivalent to that of the reactants. The evolution of the degree of localization and the effective number of centers along the IRC is shown in the following subsections.

S1.5.1 IRC Step 1

1-center FNO analysis, $\varepsilon = 0.85000$		
MO	λ	Atoms
1	1.00177	C_1
2	1.00022	F_2
3	0.99852	F_2
4	0.98070	F_2
5	0.98067	F_2
6	1.00004	F_6
7	0.99977	F_6
8	0.99260	F_6
9	0.99259	F_6
10	0.97394	F_6
**2-center FNO analysis, $\varepsilon = 0.85000$**		
MO	λ	Atoms
11	0.99226	C_1-F_2
12	0.96012	C_1-H_3
13	0.96001	C_1-H_4
14	0.96025	C_1-H_5

Localization and atoms expanded by each FNO								
FNO	Loc_1	Loc_2	Loc_3	Loc_4	Loc_5	Loc_6	Loc_7	n_{eff}
1	100.2%	0.0%	0.0%	0.0%	0.0%	0.0%	0.99647	
2	0.0%	100.0%	0.0%	0.0%	0.0%	0.0%	0.99957	
3	0.1%	99.9%	0.0%	0.0%	0.0%	0.0%	1.00296	
4	1.4%	98.1%	0.0%	0.2%	0.3%	0.0%	1.03953	
5	1.4%	98.1%	0.3%	0.1%	0.1%	0.0%	1.03960	
6	0.0%	0.0%	0.0%	0.0%	0.0%	100.0%	0.99992	
7	0.0%	0.0%	0.0%	0.0%	0.0%	100.0%	1.00046	
8	0.1%	0.0%	0.3%	0.2%	0.1%	99.3%	1.01495	
9	0.1%	0.0%	0.1%	0.2%	0.3%	99.3%	1.01498	
10	1.2%	0.2%	0.4%	0.4%	0.4%	97.4%	1.05401	
11	18.9%	80.3%	0.2%	0.2%	0.2%	0.1%	1.46803	
12	51.3%	1.9%	44.8%	0.7%	0.7%	0.6%	2.15730	
13	51.3%	1.9%	0.7%	44.7%	0.7%	0.6%	2.15772	
14	51.3%	1.9%	0.7%	0.7%	44.8%	0.6%	2.15670	

S1.5.2 IRC Step 2

1-center FNO analysis, $\varepsilon = 0.85000$		
MO	λ	Atoms
1	1.00177	C_1
2	1.00022	F_2
3	0.99853	F_2
4	0.98073	F_2
5	0.98070	F_2
6	1.00004	F_6
7	0.99974	F_6
8	0.99230	F_6
9	0.99229	F_6
10	0.97310	F_6
**2-center FNO analysis, $\varepsilon = 0.85000$**		
MO	λ	Atoms
11	0.99224	C_1-F_2
12	0.95967	C_1-H_3
13	0.96001	C_1-H_4
14	0.96022	C_1-H_5

Localization and atoms expanded by each FNO							
FNO	Loc_1	Loc_2	Loc_3	Loc_4	Loc_5	Loc_6	Loc_7 n_{eff}
1	100.2%	0.0%	0.0%	0.0%	0.0%	0.0%	0.99647
2	0.0%	100.0%	0.0%	0.0%	0.0%	0.0%	0.99957
3	0.1%	99.9%	0.0%	0.0%	0.0%	0.0%	1.00295
4	1.4%	98.1%	0.0%	0.2%	0.3%	0.0%	1.03946
5	1.4%	98.1%	0.3%	0.1%	0.1%	0.0%	1.03953
6	0.0%	0.0%	0.0%	0.0%	0.0%	100.0%	0.99992
7	0.0%	0.0%	0.0%	0.0%	0.0%	100.0%	1.00051
8	0.1%	0.0%	0.2%	0.1%	0.3%	99.2%	1.01556
9	0.1%	0.0%	0.2%	0.3%	0.1%	99.2%	1.01557
10	1.3%	0.3%	0.4%	0.4%	0.4%	97.3%	1.05581
11	18.9%	80.4%	0.2%	0.2%	0.2%	0.1%	1.46727
12	51.2%	1.9%	44.7%	0.7%	0.7%	0.7%	2.15936
13	51.2%	1.9%	0.7%	44.8%	0.7%	0.7%	2.15783
14	51.3%	1.9%	0.7%	0.7%	44.8%	0.7%	2.15682

S1.5.3 IRC Step 3

1-center FNO analysis, $\varepsilon = 0.85000$		
MO	λ	Atoms
1	1.00177	C_1
2	1.00022	F_2
3	0.99853	F_2
4	0.98074	F_2
5	0.98072	F_2
6	1.00004	F_6
7	0.99972	F_6
8	0.99199	F_6
9	0.99198	F_6
10	0.97204	F_6
2-center FNO analysis, $\varepsilon = 0.85000$		
MO	λ	Atoms
11	0.99234	C_1-F_2
12	0.95991	C_1-H_3
13	0.95968	C_1-H_4
14	0.95971	C_1-H_5

Localization and atoms expanded by each FNO							
FNO	Loc_1	Loc_2	Loc_3	Loc_4	Loc_5	Loc_6	Loc_7 n_{eff}
1	100.2%	0.0%	0.0%	0.0%	0.0%	0.0%	0.99646
2	0.0%	100.0%	0.0%	0.0%	0.0%	0.0%	0.99957
3	0.1%	99.9%	0.0%	0.0%	0.0%	0.0%	1.00295
4	1.4%	98.1%	0.3%	0.2%	0.0%	0.0%	1.03945
5	1.4%	98.1%	0.0%	0.2%	0.3%	0.0%	1.03948
6	0.0%	0.0%	0.0%	0.0%	0.0%	100.0%	0.99991
7	0.0%	0.0%	0.0%	0.0%	0.0%	100.0%	1.00057
8	0.2%	0.0%	0.1%	0.2%	0.3%	99.2%	1.01619
9	0.2%	0.0%	0.3%	0.2%	0.1%	99.2%	1.01621
10	1.3%	0.3%	0.4%	0.4%	0.4%	97.2%	1.05809
11	18.8%	80.4%	0.2%	0.2%	0.2%	0.1%	1.46645
12	51.2%	1.9%	44.7%	0.7%	0.7%	0.7%	2.15820
13	51.2%	1.9%	0.7%	44.7%	0.7%	0.7%	2.15936
14	51.2%	1.9%	0.7%	0.7%	44.8%	0.7%	2.15935

S1.5.4 IRC Step 4

1-center FNO analysis, $\varepsilon = 0.85000$		
MO	λ	Atoms
1	1.00177	C_1
2	1.00022	F_2
3	0.99853	F_2
4	0.98074	F_2
5	0.98072	F_2
6	1.00004	F_6
7	0.99969	F_6
8	0.99167	F_6
9	0.99167	F_6
10	0.97110	F_6
2-center FNO analysis, $\varepsilon = 0.85000$		
MO	λ	Atoms
11	0.99228	C_1-F_2
12	0.95963	C_1-H_3
13	0.95950	C_1-H_4
14	0.95954	C_1-H_5

Localization and atoms expanded by each FNO								
FNO	Loc_1	Loc_2	Loc_3	Loc_4	Loc_5	Loc_6	Loc_7	n_{eff}
1	100.2%	0.0%	0.0%	0.0%	0.0%	0.0%	0.99646	
2	0.0%	100.0%	0.0%	0.0%	0.0%	0.0%	0.99957	
3	0.1%	99.9%	0.0%	0.0%	0.0%	0.0%	1.00295	
4	1.4%	98.1%	0.0%	0.3%	0.2%	0.0%	1.03945	
5	1.4%	98.1%	0.3%	0.1%	0.1%	0.0%	1.03949	
6	0.0%	0.0%	0.0%	0.0%	0.0%	100.0%	0.99991	
7	0.0%	0.0%	0.0%	0.0%	0.0%	100.0%	1.00063	
8	0.2%	0.0%	0.1%	0.3%	0.3%	99.2%	1.01684	
9	0.2%	0.0%	0.3%	0.2%	0.1%	99.2%	1.01685	
10	1.4%	0.3%	0.4%	0.4%	0.4%	97.1%	1.06013	
11	18.8%	80.4%	0.2%	0.2%	0.2%	0.1%	1.46578	
12	51.2%	1.9%	44.8%	0.7%	0.7%	0.7%	2.15963	
13	51.2%	1.9%	0.7%	44.8%	0.7%	0.7%	2.16020	
14	51.2%	1.9%	0.7%	0.7%	44.8%	0.7%	2.16002	

S1.5.5 IRC Step 5

1-center FNO analysis, $\varepsilon = 0.85000$		
MO	λ	Atoms
1	1.00178	C_1
2	1.00022	F_2
3	0.99852	F_2
4	0.98072	F_2
5	0.98072	F_2
6	1.00005	F_6
7	0.99965	F_6
8	0.99134	F_6
9	0.99133	F_6
10	0.96995	F_6
2-center FNO analysis, $\varepsilon = 0.85000$		
MO	λ	Atoms
11	0.99211	C_1-F_2
12	0.95928	C_1-H_3
13	0.95926	C_1-H_4
14	0.95912	C_1-H_5

Localization and atoms expanded by each FNO								
FNO	Loc_1	Loc_2	Loc_3	Loc_4	Loc_5	Loc_6	Loc_7	n_{eff}
1	100.2%	0.0%	0.0%	0.0%	0.0%	0.0%	0.99646	
2	0.0%	100.0%	0.0%	0.0%	0.0%	0.0%	0.99957	
3	0.1%	99.9%	0.0%	0.0%	0.0%	0.0%	1.00296	
4	1.4%	98.1%	0.3%	0.0%	0.3%	0.0%	1.03949	
5	1.4%	98.1%	0.1%	0.3%	0.1%	0.0%	1.03949	
6	0.0%	0.0%	0.0%	0.0%	0.0%	100.0%	0.99991	
7	0.0%	0.0%	0.0%	0.0%	0.0%	100.0%	1.00069	
8	0.2%	0.0%	0.1%	0.2%	0.4%	99.1%	1.01753	
9	0.2%	0.0%	0.3%	0.2%	0.1%	99.1%	1.01754	
10	1.5%	0.3%	0.4%	0.4%	0.4%	97.0%	1.06260	
11	18.8%	80.5%	0.2%	0.2%	0.2%	0.1%	1.46521	
12	51.2%	1.9%	44.8%	0.7%	0.7%	0.8%	2.16129	
13	51.2%	1.9%	0.7%	44.8%	0.7%	0.8%	2.16142	
14	51.1%	1.9%	0.7%	0.7%	44.8%	0.8%	2.16208	

S1.5.6 IRC Step 6

1-center FNO analysis, $\varepsilon = 0.85000$		
MO	λ	Atoms
1	1.00178	C_1
2	1.00022	F_2
3	0.99852	F_2
4	0.98074	F_2
5	0.98073	F_2
6	1.00005	F_6
7	0.99962	F_6
8	0.99101	F_6
9	0.99099	F_6
10	0.96882	F_6
2-center FNO analysis, $\varepsilon = 0.85000$		
MO	λ	Atoms
11	0.99226	$C_1{-}F_2$
12	0.95884	$C_1{-}H_3$
13	0.95906	$C_1{-}H_4$
14	0.95858	$C_1{-}H_5$

Localization and atoms expanded by each FNO								
FNO	Loc_1	Loc_2	Loc_3	Loc_4	Loc_5	Loc_6	Loc_7	n_{eff}
1	100.2%	0.0%	0.0%	0.0%	0.0%	0.0%	0.99645	
2	0.0%	100.0%	0.0%	0.0%	0.0%	0.0%	0.99957	
3	0.1%	99.9%	0.0%	0.0%	0.0%	0.0%	1.00296	
4	1.3%	98.1%	0.0%	0.3%	0.3%	0.0%	1.03945	
5	1.4%	98.1%	0.3%	0.1%	0.1%	0.0%	1.03947	
6	0.0%	0.0%	0.0%	0.0%	0.0%	100.0%	0.99990	
7	0.0%	0.0%	0.0%	0.0%	0.0%	100.0%	1.00076	
8	0.2%	0.0%	0.4%	0.2%	0.1%	99.1%	1.01821	
9	0.2%	0.0%	0.1%	0.3%	0.3%	99.1%	1.01824	
10	1.6%	0.3%	0.4%	0.4%	0.4%	96.9%	1.06507	
11	18.7%	80.5%	0.2%	0.2%	0.2%	0.1%	1.46427	
12	51.1%	2.0%	44.8%	0.7%	0.7%	0.8%	2.16348	
13	51.1%	2.0%	0.7%	44.8%	0.7%	0.8%	2.16249	
14	51.1%	2.0%	0.7%	0.7%	44.8%	0.8%	2.16461	

S1.5.7 IRC Step 7

1-center FNO analysis, $\varepsilon = 0.85000$		
MO	λ	Atoms
1	1.00178	C_1
2	1.00022	F_2
3	0.99852	F_2
4	0.98075	F_2
5	0.98074	F_2
6	1.00005	F_6
7	0.99958	F_6
8	0.99066	F_6
9	0.99065	F_6
10	0.96766	F_6
2-center FNO analysis, $\varepsilon = 0.85000$		
MO	λ	Atoms
11	0.99261	$C_1{-}F_2$
12	0.95861	$C_1{-}H_3$
13	0.95867	$C_1{-}H_4$
14	0.95861	$C_1{-}H_5$

Localization and atoms expanded by each FNO								
FNO	Loc_1	Loc_2	Loc_3	Loc_4	Loc_5	Loc_6	Loc_7	n_{eff}
1	100.2%	0.0%	0.0%	0.0%	0.0%	0.0%	0.99645	
2	0.0%	100.0%	0.0%	0.0%	0.0%	0.0%	0.99957	
3	0.1%	99.9%	0.0%	0.0%	0.0%	0.0%	1.00296	
4	1.3%	98.1%	0.3%	0.1%	0.1%	0.0%	1.03942	
5	1.4%	98.1%	0.0%	0.3%	0.2%	0.0%	1.03944	
6	0.0%	0.0%	0.0%	0.0%	0.0%	100.0%	0.99990	
7	0.0%	0.0%	0.0%	0.0%	0.0%	100.0%	1.00083	
8	0.2%	0.0%	0.4%	0.2%	0.1%	99.1%	1.01892	
9	0.2%	0.0%	0.1%	0.3%	0.4%	99.1%	1.01894	
10	1.6%	0.3%	0.4%	0.4%	0.4%	96.8%	1.06759	
11	18.7%	80.5%	0.2%	0.2%	0.2%	0.1%	1.46365	
12	51.1%	2.0%	44.8%	0.7%	0.7%	0.8%	2.16467	
13	51.1%	2.0%	0.7%	44.8%	0.7%	0.8%	2.16441	
14	51.1%	2.0%	0.7%	0.7%	44.8%	0.8%	2.16460	

S1.5.8 IRC Step 8

1-center FNO analysis, $\varepsilon = 0.85000$		
MO	λ	Atoms
1	1.00178	C_1
2	1.00022	F_2
3	0.99852	F_2
4	0.98074	F_2
5	0.98074	F_2
6	1.00005	F_6
7	0.99954	F_6
8	0.99031	F_6
9	0.99030	F_6
10	0.96634	F_6
2-center FNO analysis, $\varepsilon = 0.85000$		
MO	λ	Atoms
11	0.99206	$C_1{-}F_2$
12	0.95838	$C_1{-}H_3$
13	0.95850	$C_1{-}H_4$
14	0.95846	$C_1{-}H_5$

Localization and atoms expanded by each FNO							
FNO	Loc_1	Loc_2	Loc_3	Loc_4	Loc_5	Loc_6	Loc_7 n_{eff}
1	100.2%	0.0%	0.0%	0.0%	0.0%	0.0%	0.99644
2	0.0%	100.0%	0.0%	0.0%	0.0%	0.0%	0.99957
3	0.1%	99.9%	0.0%	0.0%	0.0%	0.0%	1.00296
4	1.3%	98.1%	0.1%	0.3%	0.2%	0.0%	1.03945
5	1.3%	98.1%	0.3%	0.0%	0.2%	0.0%	1.03946
6	0.0%	0.0%	0.0%	0.0%	0.0%	100.0%	0.99989
7	0.0%	0.0%	0.0%	0.0%	0.0%	100.0%	1.00091
8	0.2%	0.0%	0.4%	0.2%	0.2%	99.0%	1.01964
9	0.2%	0.0%	0.1%	0.3%	0.3%	99.0%	1.01967
10	1.7%	0.3%	0.4%	0.4%	0.4%	96.6%	1.07046
11	18.7%	80.5%	0.2%	0.2%	0.2%	0.1%	1.46277
12	51.0%	2.0%	44.8%	0.7%	0.7%	0.9%	2.16587
13	51.0%	2.0%	0.7%	44.8%	0.7%	0.9%	2.16536
14	51.0%	2.0%	0.7%	0.7%	44.8%	0.8%	2.16539

S1.5.9 IRC Step 9

1-center FNO analysis, $\varepsilon = 0.85000$		
MO	λ	Atoms
1	1.00179	C_1
2	1.00022	F_2
3	0.99852	F_2
4	0.98076	F_2
5	0.98075	F_2
6	1.00006	F_6
7	0.99950	F_6
8	0.98993	F_6
9	0.98993	F_6
10	0.96499	F_6
2-center FNO analysis, $\varepsilon = 0.85000$		
MO	λ	Atoms
11	0.99205	C_1-F_2
12	0.95834	C_1-H_3
13	0.95828	C_1-H_4
14	0.95804	C_1-H_5

Localization and atoms expanded by each FNO							
FNO	Loc_1	Loc_2	Loc_3	Loc_4	Loc_5	Loc_6	Loc_7 n_{eff}
1	100.2%	0.0%	0.0%	0.0%	0.0%	0.0%	0.99644
2	0.0%	100.0%	0.0%	0.0%	0.0%	0.0%	0.99957
3	0.1%	99.9%	0.0%	0.0%	0.0%	0.0%	1.00297
4	1.3%	98.1%	0.3%	0.0%	0.2%	0.0%	1.03941
5	1.3%	98.1%	0.0%	0.3%	0.2%	0.0%	1.03943
6	0.0%	0.0%	0.0%	0.0%	0.0%	100.0%	0.99989
7	0.0%	0.0%	0.0%	0.0%	0.0%	100.0%	1.00100
8	0.2%	0.0%	0.1%	0.3%	0.4%	99.0%	1.02041
9	0.2%	0.0%	0.4%	0.2%	0.1%	99.0%	1.02042
10	1.8%	0.4%	0.4%	0.4%	0.4%	96.5%	1.07341
11	18.6%	80.6%	0.2%	0.2%	0.2%	0.1%	1.46193
12	51.0%	2.0%	44.8%	0.7%	0.7%	0.9%	2.16616
13	51.0%	2.0%	0.7%	44.8%	0.7%	0.9%	2.16648
14	51.0%	2.0%	0.7%	0.7%	44.8%	0.9%	2.16759

S1.5.10 IRC Step 10

1-center FNO analysis, $\varepsilon = 0.85000$		
MO	λ	Atoms
1	1.00179	C_1
2	1.00022	F_2
3	0.99852	F_2
4	0.98080	F_2
5	0.98077	F_2
6	1.00006	F_6
7	0.99946	F_6
8	0.98956	F_6
9	0.98956	F_6
10	0.96340	F_6
2-center FNO analysis, $\varepsilon = 0.85000$		
MO	λ	Atoms
11	0.99196	C_1-F_2
12	0.95769	C_1-H_3
13	0.95791	C_1-H_4
14	0.95787	C_1-H_5

Localization and atoms expanded by each FNO								
FNO	Loc_1	Loc_2	Loc_3	Loc_4	Loc_5	Loc_6	Loc_7	n_{eff}
1	100.2%	0.0%	0.0%	0.0%	0.0%	0.0%	0.99643	
2	0.0%	100.0%	0.0%	0.0%	0.0%	0.0%	0.99957	
3	0.1%	99.9%	0.0%	0.0%	0.0%	0.0%	1.00298	
4	1.3%	98.1%	0.0%	0.2%	0.3%	0.0%	1.03934	
5	1.3%	98.1%	0.3%	0.1%	0.1%	0.0%	1.03940	
6	0.0%	0.0%	0.0%	0.0%	0.0%	100.0%	0.99988	
7	0.0%	0.0%	0.0%	0.0%	0.0%	99.9%	1.00109	
8	0.2%	0.0%	0.3%	0.1%	0.4%	99.0%	1.02117	
9	0.2%	0.0%	0.2%	0.4%	0.2%	99.0%	1.02118	
10	1.9%	0.4%	0.4%	0.4%	0.4%	96.3%	1.07690	
11	18.6%	80.6%	0.2%	0.2%	0.2%	0.1%	1.46125	
12	50.9%	2.0%	44.9%	0.6%	0.6%	0.9%	2.16936	
13	50.9%	2.0%	0.6%	44.9%	0.7%	0.9%	2.16832	
14	50.9%	2.0%	0.6%	0.6%	44.9%	0.9%	2.16847	

S1.5.11 IRC Step 11

1-center FNO analysis, $\varepsilon = 0.85000$		
MO	λ	Atoms
1	1.00180	C_1
2	1.00022	F_2
3	0.99851	F_2
4	0.98089	F_2
5	0.98088	F_2
6	1.00006	F_6
7	0.99940	F_6
8	0.98918	F_6
9	0.98917	F_6
10	0.96156	F_6
2-center FNO analysis, $\varepsilon = 0.85000$		
MO	λ	Atoms
11	0.99183	C_1-F_2
12	0.95800	C_1-H_3
13	0.95792	C_1-H_4
14	0.95759	C_1-H_5

Localization and atoms expanded by each FNO								
FNO	Loc_1	Loc_2	Loc_3	Loc_4	Loc_5	Loc_6	Loc_7	n_{eff}
1	100.2%	0.0%	0.0%	0.0%	0.0%	0.0%	0.99640	
2	0.0%	100.0%	0.0%	0.0%	0.0%	0.0%	0.99957	
3	0.1%	99.9%	0.0%	0.0%	0.0%	0.0%	1.00299	
4	1.3%	98.1%	0.1%	0.1%	0.3%	0.0%	1.03914	
5	1.3%	98.1%	0.3%	0.2%	0.0%	0.0%	1.03917	
6	0.0%	0.0%	0.0%	0.0%	0.0%	100.0%	0.99988	
7	0.0%	0.0%	0.0%	0.0%	0.0%	99.9%	1.00119	
8	0.2%	0.0%	0.1%	0.4%	0.2%	98.9%	1.02196	
9	0.2%	0.0%	0.4%	0.1%	0.3%	98.9%	1.02198	
10	2.1%	0.4%	0.5%	0.5%	0.5%	96.2%	1.08097	
11	18.6%	80.6%	0.2%	0.2%	0.2%	0.1%	1.46167	
12	50.9%	2.0%	44.9%	0.6%	0.6%	0.9%	2.16792	
13	50.9%	2.0%	0.6%	44.9%	0.6%	0.9%	2.16838	
14	50.9%	2.0%	0.6%	0.6%	44.9%	0.9%	2.16996	

S1.5.12 IRC Step 12

1-center FNO analysis, $\varepsilon = 0.85000$		
MO	λ	Atoms
1	1.00183	C_1
2	1.00021	F_2
3	0.99850	F_2
4	0.98112	F_2
5	0.98111	F_2
6	1.00006	F_6
7	0.99934	F_6
8	0.98882	F_6
9	0.98881	F_6
10	0.95932	F_6
2-center FNO analysis, $\varepsilon = 0.85000$		
MO	λ	Atoms
11	0.99171	$C_1{-}F_2$
12	0.95775	$C_1{-}H_3$
13	0.95785	$C_1{-}H_4$
14	0.95755	$C_1{-}H_5$

Localization and atoms expanded by each FNO								
FNO	Loc_1	Loc_2	Loc_3	Loc_4	Loc_5	Loc_6	Loc_7	n_{eff}
1	100.2%	0.0%	0.0%	0.0%	0.0%	0.0%	0.99636	
2	0.0%	100.0%	0.0%	0.0%	0.0%	0.0%	0.99957	
3	0.1%	99.9%	0.0%	0.0%	0.0%	0.0%	1.00300	
4	1.3%	98.1%	0.3%	0.0%	0.2%	0.0%	1.03865	
5	1.3%	98.1%	0.0%	0.3%	0.2%	0.0%	1.03868	
6	0.0%	0.0%	0.0%	0.0%	0.0%	100.0%	0.99987	
7	0.0%	0.0%	0.0%	0.0%	0.0%	99.9%	1.00131	
8	0.3%	0.0%	0.1%	0.3%	0.4%	98.9%	1.02271	
9	0.3%	0.0%	0.4%	0.2%	0.1%	98.9%	1.02273	
10	2.2%	0.5%	0.5%	0.5%	0.5%	95.9%	1.08592	
11	18.6%	80.6%	0.2%	0.2%	0.2%	0.1%	1.46235	
12	50.9%	1.9%	44.9%	0.6%	0.6%	1.0%	2.16909	
13	50.9%	1.9%	0.6%	44.9%	0.6%	1.0%	2.16863	
14	50.9%	1.9%	0.6%	0.6%	44.9%	1.0%	2.17005	

S1.5.13 IRC Step 13

1-center FNO analysis, $\varepsilon = 0.85000$		
MO	λ	Atoms
1	1.00185	C_1
2	1.00021	F_2
3	0.99849	F_2
4	0.98147	F_2
5	0.98145	F_2
6	1.00007	F_6
7	0.99927	F_6
8	0.98847	F_6
9	0.98847	F_6
10	0.95631	F_6
2-center FNO analysis, $\varepsilon = 0.85000$		
MO	λ	Atoms
11	0.99105	$C_1{-}F_2$
12	0.95830	$C_1{-}H_3$
13	0.95805	$C_1{-}H_4$
14	0.95800	$C_1{-}H_5$

Localization and atoms expanded by each FNO								
FNO	Loc_1	Loc_2	Loc_3	Loc_4	Loc_5	Loc_6	Loc_7	n_{eff}
1	100.2%	0.0%	0.0%	0.0%	0.0%	0.0%	0.99631	
2	0.0%	100.0%	0.0%	0.0%	0.0%	0.0%	0.99958	
3	0.1%	99.8%	0.0%	0.0%	0.0%	0.0%	1.00303	
4	1.3%	98.1%	0.2%	0.0%	0.3%	0.0%	1.03792	
5	1.3%	98.1%	0.1%	0.3%	0.1%	0.0%	1.03797	
6	0.0%	0.0%	0.0%	0.0%	0.0%	100.0%	0.99986	
7	0.0%	0.0%	0.0%	0.0%	0.0%	99.9%	1.00145	
8	0.3%	0.0%	0.4%	0.1%	0.4%	98.8%	1.02342	
9	0.3%	0.0%	0.2%	0.5%	0.2%	98.8%	1.02342	
10	2.5%	0.5%	0.5%	0.5%	0.5%	95.6%	1.09264	
11	18.6%	80.5%	0.2%	0.2%	0.2%	0.1%	1.46491	
12	51.0%	1.9%	44.8%	0.6%	0.6%	1.0%	2.16626	
13	51.0%	1.9%	0.6%	44.8%	0.6%	1.0%	2.16750	
14	51.0%	1.9%	0.6%	0.6%	44.8%	1.0%	2.16772	

S1.5.14 IRC Step 14

1-center FNO analysis, $\varepsilon = 0.85000$		
MO	λ	Atoms
1	1.00187	C_1
2	1.00021	F_2
3	0.99847	F_2
4	0.98195	F_2
5	0.98193	F_2
6	1.00007	F_6
7	0.99920	F_6
8	0.98818	F_6
9	0.98817	F_6
10	0.95266	F_6
2-center FNO analysis, $\varepsilon = 0.85000$		
MO	λ	Atoms
11	0.99055	$C_1{-}F_2$
12	0.95849	$C_1{-}H_3$
13	0.95901	$C_1{-}H_4$
14	0.95877	$C_1{-}H_5$

Localization and atoms expanded by each FNO								
FNO	Loc_1	Loc_2	Loc_3	Loc_4	Loc_5	Loc_6	Loc_7	n_{eff}
1	100.2%	0.0%	0.0%	0.0%	0.0%	0.0%	0.99627	
2	0.0%	100.0%	0.0%	0.0%	0.0%	0.0%	0.99959	
3	0.1%	99.8%	0.0%	0.0%	0.0%	0.0%	1.00307	
4	1.2%	98.2%	0.0%	0.2%	0.3%	0.0%	1.03692	
5	1.2%	98.2%	0.3%	0.1%	0.1%	0.0%	1.03697	
6	0.0%	0.0%	0.0%	0.0%	0.0%	100.0%	0.99986	
7	0.0%	0.0%	0.0%	0.0%	0.0%	99.9%	1.00161	
8	0.3%	0.0%	0.2%	0.2%	0.5%	98.8%	1.02402	
9	0.3%	0.0%	0.4%	0.3%	0.1%	98.8%	1.02405	
10	2.8%	0.6%	0.5%	0.5%	0.5%	95.3%	1.10083	
11	18.6%	80.4%	0.3%	0.3%	0.3%	0.1%	1.46736	
12	51.1%	1.8%	44.8%	0.6%	0.6%	1.1%	2.16509	
13	51.1%	1.9%	0.6%	44.8%	0.6%	1.1%	2.16266	
14	51.1%	1.9%	0.6%	0.6%	44.8%	1.1%	2.16377	

S1.5.15 IRC Step 15

1-center FNO analysis, $\varepsilon = 0.85000$		
MO	λ	Atoms
1	1.00187	C_1
2	1.00020	F_2
3	0.99845	F_2
4	0.98251	F_2
5	0.98249	F_2
6	1.00008	F_6
7	0.99911	F_6
8	0.98795	F_6
9	0.98794	F_6
10	0.94813	F_6
2-center FNO analysis, $\varepsilon = 0.85000$		
MO	λ	Atoms
11	0.99021	C_1-F_2
12	0.95922	C_1-H_3
13	0.95902	C_1-H_4
14	0.95936	C_1-H_5

Localization and atoms expanded by each FNO								
FNO	Loc_1	Loc_2	Loc_3	Loc_4	Loc_5	Loc_6	Loc_7	n_{eff}
1	100.2%	0.0%	0.0%	0.0%	0.0%	0.0%	0.99628	
2	0.0%	100.0%	0.0%	0.0%	0.0%	0.0%	0.99959	
3	0.1%	99.8%	0.0%	0.0%	0.0%	0.0%	1.00311	
4	1.2%	98.3%	0.2%	0.3%	0.1%	0.0%	1.03575	
5	1.2%	98.2%	0.2%	0.0%	0.3%	0.0%	1.03579	
6	0.0%	0.0%	0.0%	0.0%	0.0%	100.0%	0.99985	
7	0.0%	0.0%	0.0%	0.0%	0.0%	99.9%	1.00177	
8	0.3%	0.0%	0.2%	0.2%	0.5%	98.8%	1.02449	
9	0.3%	0.0%	0.4%	0.4%	0.1%	98.8%	1.02453	
10	3.1%	0.6%	0.5%	0.5%	0.5%	94.8%	1.11109	
11	18.6%	80.4%	0.3%	0.3%	0.3%	0.1%	1.46821	
12	51.2%	1.8%	44.7%	0.6%	0.6%	1.1%	2.16130	
13	51.2%	1.8%	0.6%	44.7%	0.6%	1.1%	2.16225	
14	51.2%	1.8%	0.6%	0.6%	44.7%	1.1%	2.16067	

S1.5.16 IRC Step 16

1-center FNO analysis, $\varepsilon = 0.85000$		
MO	λ	Atoms
1	1.00183	C_1
2	1.00020	F_2
3	0.99843	F_2
4	0.98304	F_2
5	0.98303	F_2
6	1.00008	F_6
7	0.99903	F_6
8	0.98777	F_6
9	0.98776	F_6
10	0.94292	F_6
2-center FNO analysis, $\varepsilon = 0.85000$		
MO	λ	Atoms
11	0.98966	C_1-F_2
12	0.95978	C_1-H_3
13	0.95976	C_1-H_4
14	0.95986	C_1-H_5

Localization and atoms expanded by each FNO								
FNO	Loc_1	Loc_2	Loc_3	Loc_4	Loc_5	Loc_6	Loc_7	n_{eff}
1	100.2%	0.0%	0.0%	0.0%	0.0%	0.0%	0.99634	
2	0.0%	100.0%	0.0%	0.0%	0.0%	0.0%	0.99960	
3	0.1%	99.8%	0.0%	0.0%	0.0%	0.0%	1.00314	
4	1.1%	98.3%	0.2%	0.0%	0.2%	0.0%	1.03464	
5	1.1%	98.3%	0.1%	0.3%	0.1%	0.0%	1.03467	
6	0.0%	0.0%	0.0%	0.0%	0.0%	100.0%	0.99984	
7	0.0%	0.0%	0.0%	0.0%	0.0%	99.9%	1.00194	
8	0.4%	0.0%	0.4%	0.1%	0.3%	98.8%	1.02486	
9	0.4%	0.0%	0.1%	0.4%	0.3%	98.8%	1.02490	
10	3.6%	0.7%	0.5%	0.5%	0.5%	94.3%	1.12299	
11	18.5%	80.5%	0.3%	0.3%	0.3%	0.1%	1.46691	
12	51.4%	1.7%	44.6%	0.6%	0.6%	1.1%	2.15822	
13	51.3%	1.7%	0.6%	44.6%	0.6%	1.1%	2.15832	
14	51.4%	1.7%	0.6%	0.6%	44.6%	1.1%	2.15786	

S1.5.17 IRC Step 17

1-center FNO analysis, $\varepsilon = 0.85000$		
MO	λ	Atoms
1	1.00178	C_1
2	1.00019	F_2
3	0.99842	F_2
4	0.98359	F_2
5	0.98356	F_2
6	1.00008	F_6
7	0.99895	F_6
8	0.98761	F_6
9	0.98760	F_6
10	0.93691	F_6
2-center FNO analysis, $\varepsilon = 0.85000$		
MO	λ	Atoms
11	0.98918	C_1-F_2
12	0.96030	C_1-H_3
13	0.96039	C_1-H_4
14	0.96050	C_1-H_5

FNO	Loc_1	Loc_2	Loc_3	Loc_4	Loc_5	Loc_6	Loc_7	n_{eff}
Localization and atoms expanded by each FNO								
1	100.2%	0.0%	0.0%	0.0%	0.0%	0.0%	0.99645	
2	0.0%	100.0%	0.0%	0.0%	0.0%	0.0%	0.99961	
3	0.1%	99.8%	0.0%	0.0%	0.0%	0.0%	1.00318	
4	1.1%	98.4%	0.0%	0.2%	0.3%	0.0%	1.03351	
5	1.1%	98.4%	0.3%	0.1%	0.1%	0.0%	1.03356	
6	0.0%	0.0%	0.0%	0.0%	0.0%	100.0%	0.99983	
7	0.0%	0.0%	0.0%	0.0%	0.0%	99.9%	1.00210	
8	0.4%	0.0%	0.2%	0.4%	0.2%	98.8%	1.02521	
9	0.4%	0.0%	0.4%	0.1%	0.4%	98.8%	1.02523	
10	4.1%	0.8%	0.5%	0.5%	0.5%	93.7%	1.13690	
11	18.3%	80.6%	0.3%	0.3%	0.3%	0.1%	1.46263	
12	51.5%	1.7%	44.6%	0.6%	0.6%	1.1%	2.15540	
13	51.5%	1.7%	0.6%	44.6%	0.6%	1.1%	2.15496	
14	51.5%	1.7%	0.6%	0.6%	44.6%	1.1%	2.15447	

S1.5.18 IRC Step 18

MO	λ	Atoms
1-center FNO analysis, $\varepsilon = 0.85000$		
1	1.00171	C_1
2	1.00019	F_2
3	0.99841	F_2
4	0.98411	F_2
5	0.98408	F_2
6	1.00009	F_6
7	0.99887	F_6
8	0.98749	F_6
9	0.98749	F_6
10	0.92997	F_6
2-center FNO analysis, $\varepsilon = 0.85000$		
MO	λ	Atoms
11	0.98912	C_1-F_2
12	0.96106	C_1-H_3
13	0.96101	C_1-H_4
14	0.96113	C_1-H_5

FNO	Loc_1	Loc_2	Loc_3	Loc_4	Loc_5	Loc_6	Loc_7	n_{eff}
Localization and atoms expanded by each FNO								
1	100.2%	0.0%	0.0%	0.0%	0.0%	0.0%	0.99659	
2	0.0%	100.0%	0.0%	0.0%	0.0%	0.0%	0.99962	
3	0.1%	99.8%	0.0%	0.0%	0.0%	0.0%	1.00319	
4	1.0%	98.4%	0.1%	0.1%	0.3%	0.0%	1.03243	
5	1.0%	98.4%	0.3%	0.2%	0.0%	0.0%	1.03249	
6	0.0%	0.0%	0.0%	0.0%	0.0%	100.0%	0.99982	
7	0.1%	0.0%	0.0%	0.0%	0.0%	99.9%	1.00226	
8	0.4%	0.0%	0.4%	0.3%	0.1%	98.7%	1.02544	
9	0.4%	0.0%	0.1%	0.3%	0.4%	98.7%	1.02545	
10	4.7%	0.9%	0.5%	0.5%	0.5%	93.0%	1.15320	
11	18.0%	80.9%	0.3%	0.3%	0.3%	0.1%	1.45460	
12	51.6%	1.6%	44.5%	0.5%	0.5%	1.2%	2.15155	
13	51.6%	1.6%	0.5%	44.5%	0.5%	1.2%	2.15189	
14	51.6%	1.6%	0.5%	0.5%	44.5%	1.2%	2.15127	

S1.5.19 IRC Step 19

1-center FNO analysis, $\varepsilon = 0.85000$		
MO	λ	Atoms
1	1.00163	C_1
2	1.00018	F_2
3	0.99840	F_2
4	0.98455	F_2
5	0.98454	F_2
6	1.00010	F_6
7	0.99879	F_6
8	0.98740	F_6
9	0.98737	F_6
10	0.92226	F_6
2-center FNO analysis, $\varepsilon = 0.85000$		
MO	λ	Atoms
11	0.98838	C_1-F_2
12	0.96119	C_1-H_3
13	0.96116	C_1-H_4
14	0.96110	C_1-H_5

Localization and atoms expanded by each FNO								
FNO	Loc_1	Loc_2	Loc_3	Loc_4	Loc_5	Loc_6	Loc_7	n_{eff}
1	100.2%	0.0%	0.0%	0.0%	0.0%	0.0%	0.99675	
2	0.0%	100.0%	0.0%	0.0%	0.0%	0.0%	0.99964	
3	0.1%	99.8%	0.0%	0.0%	0.0%	0.0%	1.00322	
4	1.0%	98.5%	0.2%	0.2%	0.0%	0.0%	1.03152	
5	1.0%	98.5%	0.1%	0.1%	0.3%	0.0%	1.03155	
6	0.0%	0.0%	0.0%	0.0%	0.0%	100.0%	0.99981	
7	0.1%	0.0%	0.0%	0.0%	0.0%	99.9%	1.00242	
8	0.5%	0.0%	0.3%	0.4%	0.1%	98.7%	1.02564	
9	0.5%	0.0%	0.2%	0.1%	0.4%	98.7%	1.02569	
10	5.3%	0.9%	0.5%	0.5%	0.5%	92.2%	1.17157	
11	17.6%	81.3%	0.3%	0.3%	0.3%	0.2%	1.44640	
12	51.6%	1.6%	44.5%	0.5%	0.5%	1.2%	2.15082	
13	51.6%	1.6%	0.5%	44.5%	0.5%	1.2%	2.15086	
14	51.6%	1.6%	0.5%	0.5%	44.5%	1.2%	2.15119	

S1.5.20 IRC Step 20

1-center FNO analysis, $\varepsilon = 0.85000$		
MO	λ	Atoms
1	1.00155	C_1
2	1.00018	F_2
3	0.99839	F_2
4	0.98499	F_2
5	0.98497	F_2
6	1.00010	F_6
7	0.99872	F_6
8	0.98729	F_6
9	0.98728	F_6
10	0.91398	F_6
2-center FNO analysis, $\varepsilon = 0.85000$		
MO	λ	Atoms
11	0.98727	C_1-F_2
12	0.96166	C_1-H_3
13	0.96150	C_1-H_4
14	0.96172	C_1-H_5

Localization and atoms expanded by each FNO								
FNO	Loc_1	Loc_2	Loc_3	Loc_4	Loc_5	Loc_6	Loc_7	n_{eff}
1	100.2%	0.0%	0.0%	0.0%	0.0%	0.0%	0.99692	
2	0.0%	100.0%	0.0%	0.0%	0.0%	0.0%	0.99965	
3	0.1%	99.8%	0.0%	0.0%	0.0%	0.0%	1.00323	
4	0.9%	98.5%	0.0%	0.2%	0.2%	0.0%	1.03061	
5	0.9%	98.5%	0.3%	0.1%	0.1%	0.0%	1.03065	
6	0.0%	0.0%	0.0%	0.0%	0.0%	100.0%	0.99980	
7	0.1%	0.0%	0.0%	0.0%	0.0%	99.9%	1.00256	
8	0.5%	0.0%	0.2%	0.1%	0.4%	98.7%	1.02586	
9	0.5%	0.0%	0.3%	0.4%	0.1%	98.7%	1.02588	
10	6.0%	1.0%	0.5%	0.5%	0.5%	91.4%	1.19161	
11	17.0%	81.7%	0.4%	0.4%	0.4%	0.2%	1.43612	
12	51.7%	1.6%	44.5%	0.5%	0.5%	1.2%	2.14859	
13	51.7%	1.6%	0.5%	44.5%	0.5%	1.2%	2.14930	
14	51.7%	1.6%	0.5%	0.5%	44.5%	1.2%	2.14841	

S1.5.21 IRC Step 21

1-center FNO analysis, $\varepsilon = 0.85000$		
MO	λ	Atoms
1	1.00146	C_1
2	1.00017	F_2
3	0.99839	F_2
4	0.98539	F_2
5	0.98536	F_2
6	1.00011	F_6
7	0.99865	F_6
8	0.98720	F_6
9	0.98718	F_6
10	0.90506	F_6
2-center FNO analysis, $\varepsilon = 0.85000$		
MO	λ	Atoms
11	0.98654	C_1-F_2
12	0.96175	C_1-H_3
13	0.96176	C_1-H_4
14	0.96233	C_1-H_5

Localization and atoms expanded by each FNO								
FNO	Loc_1	Loc_2	Loc_3	Loc_4	Loc_5	Loc_6	Loc_7	n_{eff}
1	100.1%	0.0%	0.0%	0.0%	0.0%	0.0%	0.99708	
2	0.0%	100.0%	0.0%	0.0%	0.0%	0.0%	0.99966	
3	0.1%	99.8%	0.0%	0.0%	0.0%	0.0%	1.00322	
4	0.9%	98.5%	0.1%	0.1%	0.3%	0.0%	1.02978	
5	0.9%	98.5%	0.2%	0.2%	0.0%	0.0%	1.02983	
6	0.0%	0.0%	0.0%	0.0%	0.0%	100.0%	0.99978	
7	0.1%	0.0%	0.0%	0.0%	0.0%	99.9%	1.00270	
8	0.5%	0.0%	0.3%	0.1%	0.4%	98.7%	1.02605	
9	0.5%	0.0%	0.2%	0.4%	0.1%	98.7%	1.02609	
10	6.8%	1.1%	0.5%	0.5%	0.5%	90.5%	1.21356	
11	16.4%	82.2%	0.4%	0.4%	0.4%	0.2%	1.42244	
12	51.7%	1.5%	44.5%	0.5%	0.5%	1.3%	2.14813	
13	51.7%	1.5%	0.5%	44.5%	0.5%	1.3%	2.14804	
14	51.7%	1.5%	0.5%	0.5%	44.5%	1.3%	2.14538	

S1.5.22 IRC Step 22

1-center FNO analysis, $\varepsilon = 0.85000$		
MO	λ	Atoms
1	1.00146	C_1
2	1.00016	F_2
3	0.99840	F_2
4	0.98572	F_2
5	0.98570	F_2
6	1.00011	F_6
7	0.99860	F_6
8	0.98707	F_6
9	0.98706	F_6
10	0.89611	F_6
2-center FNO analysis, $\varepsilon = 0.85000$		
MO	λ	Atoms
11	0.98571	C_1-F_2
12	0.96207	C_1-H_3
13	0.96196	C_1-H_4
14	0.96193	C_1-H_5

Localization and atoms expanded by each FNO								
FNO	Loc_1	Loc_2	Loc_3	Loc_4	Loc_5	Loc_6	Loc_7	n_{eff}
1	100.1%	0.0%	0.0%	0.0%	0.0%	0.0%	0.99708	
2	0.0%	100.0%	0.0%	0.0%	0.0%	0.0%	0.99968	
3	0.1%	99.8%	0.0%	0.0%	0.0%	0.0%	1.00321	
4	0.8%	98.6%	0.1%	0.3%	0.1%	0.0%	1.02911	
5	0.8%	98.6%	0.2%	0.1%	0.3%	0.0%	1.02913	
6	0.0%	0.0%	0.0%	0.0%	0.0%	100.0%	0.99977	
7	0.1%	0.0%	0.0%	0.0%	0.0%	99.9%	1.00281	
8	0.6%	0.0%	0.2%	0.4%	0.2%	98.7%	1.02631	
9	0.6%	0.0%	0.3%	0.1%	0.3%	98.7%	1.02633	
10	7.6%	1.2%	0.5%	0.5%	0.5%	89.6%	1.23598	
11	15.7%	82.8%	0.4%	0.4%	0.4%	0.2%	1.40653	
12	51.7%	1.5%	44.5%	0.5%	0.5%	1.3%	2.14680	
13	51.7%	1.5%	0.5%	44.5%	0.5%	1.3%	2.14720	
14	51.7%	1.5%	0.5%	0.5%	44.5%	1.3%	2.14737	

S1.5.23 IRC Step 23

1-center FNO analysis, $\varepsilon = 0.85000$		
MO	λ	Atoms
1	1.00147	C_1
2	1.00015	F_2
3	0.99842	F_2
4	0.98602	F_2
5	0.98601	F_2
6	0.85475	F_2
7	1.00012	F_6
8	0.99855	F_6
9	0.98691	F_6
10	0.98691	F_6
11	0.88734	F_6
2-center FNO analysis, $\varepsilon = 0.85000$		
MO	λ	Atoms
12	0.96234	C_1-H_3
13	0.96207	C_1-H_4
14	0.96190	C_1-H_5

Localization and atoms expanded by each FNO								
FNO	Loc_1	Loc_2	Loc_3	Loc_4	Loc_5	Loc_6	Loc_7	n_{eff}
1	100.1%	0.0%	0.0%	0.0%	0.0%	0.0%	0.99708	
2	0.0%	100.0%	0.0%	0.0%	0.0%	0.0%	0.99970	
3	0.1%	99.8%	0.0%	0.0%	0.0%	0.0%	1.00317	
4	0.8%	98.6%	0.3%	0.2%	0.1%	0.0%	1.02848	
5	0.8%	98.6%	0.1%	0.1%	0.3%	0.0%	1.02849	
6	11.8%	85.5%	0.5%	0.5%	0.5%	1.2%	1.34290	
7	0.0%	0.0%	0.0%	0.0%	0.0%	100.0%	0.99976	
8	0.1%	0.0%	0.0%	0.0%	0.0%	99.9%	1.00291	
9	0.6%	0.0%	0.3%	0.3%	0.1%	98.7%	1.02664	
10	0.6%	0.0%	0.2%	0.1%	0.4%	98.7%	1.02666	
11	8.5%	1.2%	0.5%	0.5%	0.5%	88.7%	1.25821	
12	51.7%	1.4%	44.5%	0.5%	0.5%	1.3%	2.14572	
13	51.7%	1.4%	0.5%	44.5%	0.5%	1.3%	2.14684	
14	51.7%	1.4%	0.5%	0.5%	44.5%	1.3%	2.14771	

S1.5.24 IRC Step 24

1-center FNO analysis, $\varepsilon = 0.85000$		
MO	λ	Atoms
1	1.00147	C_1
2	1.00014	F_2
3	0.99846	F_2
4	0.98653	F_2
5	0.98653	F_2
6	0.87006	F_2
7	1.00014	F_6
8	0.99846	F_6
9	0.98653	F_6
10	0.98653	F_6
11	0.87006	F_6
2-center FNO analysis, $\varepsilon = 0.85000$		
MO	λ	Atoms
12	0.96200	C_1-H_3
13	0.96207	C_1-H_4
14	0.96170	C_1-H_5

Localization and atoms expanded by each FNO								
FNO	Loc_1	Loc_2	Loc_3	Loc_4	Loc_5	Loc_6	Loc_7	n_{eff}
1	100.1%	0.0%	0.0%	0.0%	0.0%	0.0%	0.99707	
2	0.0%	100.0%	0.0%	0.0%	0.0%	0.0%	0.99973	
3	0.1%	99.8%	0.0%	0.0%	0.0%	0.0%	1.00308	
4	0.7%	98.7%	0.3%	0.3%	0.1%	0.0%	1.02743	
5	0.7%	98.7%	0.2%	0.1%	0.3%	0.0%	1.02743	
6	10.1%	87.0%	0.5%	0.5%	0.5%	1.2%	1.30293	
7	0.0%	0.0%	0.0%	0.0%	0.0%	100.0%	0.99973	
8	0.1%	0.0%	0.0%	0.0%	0.0%	99.8%	1.00308	
9	0.7%	0.0%	0.3%	0.3%	0.1%	98.7%	1.02743	
10	0.7%	0.0%	0.2%	0.1%	0.3%	98.7%	1.02743	
11	10.1%	1.2%	0.5%	0.5%	0.5%	87.0%	1.30293	
12	51.7%	1.4%	44.5%	0.5%	0.5%	1.4%	2.14734	
13	51.7%	1.4%	0.5%	44.5%	0.5%	1.4%	2.14684	
14	51.7%	1.4%	0.5%	0.5%	44.5%	1.4%	2.14857	

S1.6 FeF_6^{3-}

S1.6.1 FeF_6^{3-} *Oh* High spin, ($t_{2g}^3 e_g^2 -^6 A_{1g}$)

1-center FNO analysis, $\varepsilon = 0.70000$, $\alpha-$**orbitals**		
MO	λ	Atoms
1	1.00000	Fe_1
2	1.00000	Fe_1
3	1.00000	Fe_1
4	1.00000	Fe_1
5	1.00000	Fe_1
6	0.99885	Fe_1
7	0.99709	Fe_1
8	0.99709	Fe_1
9	0.99709	Fe_1
10	0.97411	Fe_1
11	0.97411	Fe_1
12	0.97411	Fe_1
13	0.97035	Fe_1
14	0.97035	Fe_1
15	1.00120	F_2
16	1.00000	F_2
17	0.98602	F_2
18	0.98602	F_2
19	0.95217	F_2
20	1.00120	F_3
21	1.00000	F_3
22	0.98602	F_3
23	0.98602	F_3
24	0.95217	F_3
25	1.00120	F_4
26	1.00000	F_4
27	0.98602	F_4
28	0.98602	F_4
29	0.95217	F_4
30	1.00120	F_5
31	1.00000	F_5
32	0.98602	F_5
33	0.98602	F_5
34	0.95217	F_5
35	1.00120	F_6
36	1.00000	F_6
37	0.98602	F_6
38	0.98602	F_6
39	0.95217	F_6
40	1.00000	F_7
41	0.99805	F_7
42	0.98318	F_7
43	0.98318	F_7
44	0.95047	F_7

Localization and atoms expanded by each FNO, $\alpha-$**orbitals**								
FNO	Loc_1	Loc_2	Loc_3	Loc_4	Loc_5	Loc_6	Loc_7	n_{eff}
1	100.0%	-0.0%	-0.0%	-0.0%	-0.0%	-0.0%	-0.0%	1.00000
2	100.0%	-0.0%	-0.0%	-0.0%	-0.0%	-0.0%	0.0%	1.00000
3	100.0%	-0.0%	-0.0%	-0.0%	-0.0%	-0.0%	0.0%	1.00000
4	100.0%	-0.0%	-0.0%	-0.0%	-0.0%	-0.0%	0.0%	1.00000
5	100.0%	-0.0%	-0.0%	-0.0%	-0.0%	-0.0%	0.0%	1.00000
6	99.9%	0.0%	0.0%	0.0%	0.0%	0.0%	0.0%	1.00231
7	99.7%	0.1%	0.1%	0.1%	0.1%	0.0%	0.0%	1.00584
8	99.7%	0.0%	0.0%	0.0%	0.0%	0.1%	0.1%	1.00584
9	99.7%	0.1%	0.1%	0.1%	0.1%	0.0%	0.0%	1.00584
10	97.4%	0.6%	0.2%	0.6%	0.2%	0.5%	0.5%	1.05371
11	97.4%	0.6%	0.5%	0.6%	0.5%	0.2%	0.2%	1.05371
12	97.4%	0.1%	0.6%	0.1%	0.6%	0.6%	0.6%	1.05370
13	97.0%	0.6%	0.9%	0.6%	0.9%	0.1%	0.1%	1.06182
14	97.0%	0.4%	0.1%	0.4%	0.1%	0.9%	0.9%	1.06182
15	0.0%	100.1%	0.0%	0.2%	0.0%	0.0%	-0.3%	0.99760
16	-0.0%	100.0%	0.0%	0.0%	0.0%	0.0%	0.0%	1.00000
17	0.7%	98.6%	0.2%	0.0%	0.2%	0.3%	-0.0%	1.02849
18	0.7%	98.6%	0.3%	0.0%	0.3%	0.2%	-0.1%	1.02849
19	3.9%	95.2%	0.2%	0.0%	0.2%	0.2%	0.1%	1.10107
20	0.0%	0.0%	100.1%	0.0%	0.2%	0.0%	-0.3%	0.99760
21	-0.0%	0.0%	100.0%	0.0%	0.0%	0.0%	0.0%	1.00000
22	0.7%	0.3%	98.6%	0.3%	0.0%	0.1%	-0.2%	1.02848
23	0.7%	0.1%	98.6%	0.1%	0.0%	0.3%	0.1%	1.02849
24	3.9%	0.2%	95.2%	0.2%	0.0%	0.2%	0.1%	1.10107
25	0.0%	0.2%	0.0%	100.1%	0.0%	0.0%	-0.3%	0.99760
26	-0.0%	0.0%	0.0%	100.0%	0.0%	0.0%	0.0%	1.00000
27	0.7%	0.0%	0.4%	98.6%	0.4%	0.1%	-0.2%	1.02847
28	0.7%	0.0%	0.1%	98.6%	0.1%	0.4%	0.1%	1.02849
29	3.9%	0.0%	0.2%	95.2%	0.2%	0.2%	0.1%	1.10107
30	0.0%	0.0%	0.2%	0.0%	100.1%	0.0%	-0.3%	0.99760
31	-0.0%	0.0%	0.0%	0.0%	100.0%	0.0%	0.0%	1.00000
32	0.7%	0.1%	0.0%	0.1%	98.6%	0.4%	0.1%	1.02849
33	0.7%	0.4%	0.0%	0.4%	98.6%	0.1%	-0.2%	1.02848
34	3.9%	0.2%	0.0%	0.2%	95.2%	0.2%	0.1%	1.10107
35	0.0%	0.0%	0.0%	0.0%	0.0%	100.1%	-0.2%	0.99761
36	-0.0%	0.0%	0.0%	0.0%	0.0%	100.0%	0.0%	1.00000
37	0.7%	0.4%	0.1%	0.4%	0.1%	98.6%	-0.3%	1.02847
38	0.7%	0.1%	0.4%	0.1%	0.4%	98.6%	-0.3%	1.02847
39	3.9%	0.2%	0.2%	0.2%	0.2%	95.2%	-0.1%	1.10106
40	-0.0%	0.0%	0.0%	0.0%	0.0%	0.0%	100.0%	1.00000
41	0.0%	0.0%	0.0%	0.0%	0.0%	0.2%	99.8%	1.00391
42	0.7%	0.1%	0.4%	0.1%	0.4%	0.0%	98.3%	1.03442
43	0.7%	0.4%	0.1%	0.4%	0.1%	0.0%	98.3%	1.03442
44	3.9%	0.2%	0.2%	0.2%	0.2%	0.0%	95.0%	1.10500

1-center FNO analysis, $\varepsilon = 0.70000$, $\beta-$**orbitals**		
MO	λ	Atoms
1	1.00000	Fe_1
2	1.00000	Fe_1
3	1.00000	Fe_1
4	1.00000	Fe_1
5	1.00000	Fe_1
6	0.99879	Fe_1
7	0.99658	Fe_1
8	0.99658	Fe_1
9	0.99658	Fe_1
10	1.00091	F_2
11	1.00000	F_2
12	0.97579	F_2
13	0.97579	F_2
14	0.91584	F_2
15	1.00091	F_3
16	1.00000	F_3
17	0.97579	F_3
18	0.97579	F_3
19	0.91584	F_3
20	1.00091	F_4
21	1.00000	F_4
22	0.97579	F_4
23	0.97579	F_4
24	0.91584	F_4
25	1.00091	F_5
26	1.00000	F_5
27	0.97579	F_5
28	0.97579	F_5
29	0.91584	F_5
30	1.00091	F_6
31	1.00000	F_6
32	0.97579	F_6
33	0.97579	F_6
34	0.91584	F_6
35	1.00000	F_7
36	0.99730	F_7
37	0.97272	F_7
38	0.97272	F_7
39	0.91408	F_7

Localization and atoms expanded by each FNO, $\beta-$**orbitals**								
FNO	Loc_1	Loc_2	Loc_3	Loc_4	Loc_5	Loc_6	Loc_7	n_{eff}
1	100.0%	-0.0%	-0.0%	-0.0%	-0.0%	-0.0%	-0.0%	1.00000
2	100.0%	-0.0%	-0.0%	-0.0%	-0.0%	-0.0%	0.0%	1.00000
3	100.0%	-0.0%	-0.0%	-0.0%	-0.0%	-0.0%	0.0%	1.00000
4	100.0%	-0.0%	-0.0%	-0.0%	-0.0%	-0.0%	0.0%	1.00000
5	100.0%	0.0%	0.0%	0.0%	0.0%	0.0%	0.0%	1.00000
6	99.9%	0.0%	0.0%	0.0%	0.0%	0.0%	0.0%	1.00242
7	99.7%	0.0%	0.1%	0.0%	0.1%	0.0%	0.0%	1.00688
8	99.7%	0.1%	0.0%	0.1%	0.0%	0.1%	0.1%	1.00688
9	99.7%	0.1%	0.0%	0.1%	0.0%	0.1%	0.1%	1.00688
10	0.1%	100.1%	0.0%	0.2%	0.0%	0.0%	-0.4%	0.99817
11	0.0%	100.0%	0.0%	0.0%	0.0%	0.0%	-0.0%	1.00000
12	1.5%	97.6%	0.1%	0.0%	0.1%	0.5%	0.2%	1.04995
13	1.5%	97.6%	0.5%	0.0%	0.5%	0.1%	-0.2%	1.04993
14	7.6%	91.6%	0.2%	0.2%	0.2%	0.2%	0.0%	1.18414
15	0.1%	0.0%	100.1%	0.0%	0.2%	0.0%	-0.4%	0.99817
16	0.0%	0.0%	100.0%	0.0%	0.0%	0.0%	-0.0%	1.00000
17	1.5%	0.5%	97.6%	0.5%	0.0%	0.1%	-0.2%	1.04993
18	1.5%	0.1%	97.6%	0.1%	0.0%	0.5%	0.2%	1.04995
19	7.6%	0.2%	91.6%	0.2%	0.2%	0.2%	0.0%	1.18414
20	0.1%	0.2%	0.0%	100.1%	0.0%	0.0%	-0.4%	0.99817
21	0.0%	0.0%	0.0%	100.0%	0.0%	0.0%	-0.0%	1.00000
22	1.5%	0.0%	0.5%	97.6%	0.5%	0.1%	-0.2%	1.04992
23	1.5%	0.0%	0.1%	97.6%	0.1%	0.5%	0.2%	1.04995
24	7.6%	0.2%	0.2%	91.6%	0.2%	0.2%	0.0%	1.18414
25	0.1%	0.0%	0.2%	0.0%	100.1%	0.0%	-0.4%	0.99817
26	0.0%	0.0%	0.0%	0.0%	100.0%	0.0%	-0.0%	1.00000
27	1.5%	0.4%	0.0%	0.4%	97.6%	0.2%	-0.1%	1.04994
28	1.5%	0.2%	0.0%	0.2%	97.6%	0.4%	0.1%	1.04996
29	7.6%	0.2%	0.2%	0.2%	91.6%	0.2%	0.0%	1.18414
30	0.1%	0.0%	0.0%	0.0%	0.0%	100.1%	-0.2%	0.99818
31	0.0%	0.0%	0.0%	0.0%	0.0%	100.0%	0.0%	1.00000
32	1.5%	0.5%	0.1%	0.5%	0.1%	97.6%	-0.3%	1.04992
33	1.5%	0.1%	0.5%	0.1%	0.5%	97.6%	-0.3%	1.04992
34	7.6%	0.2%	0.2%	0.2%	0.2%	91.6%	0.0%	1.18414
35	-0.0%	0.0%	0.0%	0.0%	0.0%	0.0%	100.0%	1.00000
36	0.1%	0.0%	0.0%	0.0%	0.0%	0.2%	99.7%	1.00541
37	1.5%	0.5%	0.1%	0.5%	0.1%	0.0%	97.3%	1.05657
38	1.5%	0.1%	0.5%	0.1%	0.5%	0.0%	97.3%	1.05657
39	7.6%	0.2%	0.2%	0.2%	0.2%	0.2%	91.4%	1.18868

S1.6.2 $\mathbf{FeF_6^{3-}}$ D_{4h} Low spin ($\mathbf{e}_g^4\mathbf{b}_{2g}^1 -^2 B_{2g}$)

1-center FNO analysis, $\varepsilon = 0.70000$ $\alpha-$**orbitals**		
MO	λ	Atoms
1	1.00000	Fe_1
2	1.00000	Fe_1
3	1.00000	Fe_1
4	1.00000	Fe_1
5	1.00000	Fe_1
6	0.99817	Fe_1
7	0.99621	Fe_1
8	0.99544	Fe_1
9	0.99544	Fe_1
10	0.96609	Fe_1
11	0.96609	Fe_1
12	0.96536	Fe_1
13	1.00067	F_2
14	1.00000	F_2
15	0.98433	F_2
16	0.98409	F_2
17	0.89917	F_2
18	1.00069	F_3
19	1.00000	F_3
20	0.98465	F_3
21	0.98465	F_3
22	0.90925	F_3
23	1.00067	F_4
24	1.00000	F_4
25	0.98433	F_4
26	0.98409	F_4
27	0.89917	F_4
28	1.00069	F_5
29	1.00000	F_5
30	0.98465	F_5
31	0.98465	F_5
32	0.90925	F_5
33	1.00067	F_6
34	1.00000	F_6
35	0.98433	F_6
36	0.98409	F_6
37	0.89917	F_6
38	1.00000	F_7
39	0.99634	F_7
40	0.98375	F_7
41	0.98167	F_7
42	0.89822	F_7

Localization and atoms expanded by each FNO $\alpha-$**orbitals**								
FNO	Loc_1	Loc_2	Loc_3	Loc_4	Loc_5	Loc_6	Loc_7	n_{eff}
1	100.0%	-0.0%	-0.0%	-0.0%	-0.0%	-0.0%	0.0%	1.00000
2	100.0%	-0.0%	-0.0%	-0.0%	-0.0%	-0.0%	0.0%	1.00000
3	100.0%	-0.0%	-0.0%	-0.0%	-0.0%	-0.0%	0.0%	1.00000
4	100.0%	-0.0%	-0.0%	-0.0%	-0.0%	-0.0%	0.0%	1.00000
5	100.0%	-0.0%	0.0%	-0.0%	0.0%	-0.0%	0.0%	1.00000
6	99.8%	0.0%	0.0%	0.0%	0.0%	0.0%	0.0%	1.00367
7	99.6%	0.0%	0.1%	0.0%	0.1%	0.0%	0.0%	1.00762
8	99.5%	0.1%	0.0%	0.1%	0.0%	0.1%	0.1%	1.00917
9	99.5%	0.1%	0.0%	0.1%	0.0%	0.1%	0.1%	1.00917
10	96.6%	0.9%	0.7%	0.9%	0.7%	0.1%	0.1%	1.07115
11	96.6%	0.1%	0.7%	0.1%	0.7%	0.9%	0.9%	1.07115
12	96.5%	0.8%	0.1%	0.8%	0.1%	0.8%	0.8%	1.07273
13	0.1%	100.1%	0.0%	0.2%	0.0%	0.0%	-0.4%	0.99864
14	0.0%	100.0%	0.0%	0.0%	0.0%	0.0%	-0.0%	1.00000
15	0.8%	98.4%	0.1%	0.0%	0.1%	0.5%	0.2%	1.03199
16	0.6%	98.4%	0.4%	0.0%	0.4%	0.1%	0.0%	1.03252
17	9.0%	89.9%	0.2%	0.4%	0.2%	0.2%	0.1%	1.22460
18	0.1%	0.0%	100.1%	0.0%	0.2%	0.0%	-0.4%	0.99861
19	0.0%	0.0%	100.0%	0.0%	0.0%	0.0%	-0.0%	1.00000
20	0.5%	0.4%	98.5%	0.4%	0.0%	0.1%	0.1%	1.03136
21	0.5%	0.1%	98.5%	0.1%	0.0%	0.4%	0.4%	1.03136
22	8.0%	0.2%	90.9%	0.2%	0.3%	0.2%	0.2%	1.20021
23	0.1%	0.2%	0.0%	100.1%	0.0%	0.0%	-0.4%	0.99864
24	0.0%	0.0%	0.0%	100.0%	0.0%	0.0%	-0.0%	1.00000
25	0.8%	0.0%	0.1%	98.4%	0.1%	0.5%	0.2%	1.03199
26	0.6%	0.0%	0.4%	98.4%	0.4%	0.1%	0.0%	1.03252
27	9.0%	0.4%	0.2%	89.9%	0.2%	0.2%	0.1%	1.22460
28	0.1%	0.0%	0.2%	0.0%	100.1%	0.0%	-0.4%	0.99861
29	0.0%	0.0%	0.0%	0.0%	100.0%	0.0%	-0.0%	1.00000
30	0.5%	0.1%	0.0%	0.1%	98.5%	0.4%	0.4%	1.03135
31	0.5%	0.4%	0.0%	0.4%	98.5%	0.1%	0.1%	1.03135
32	8.0%	0.2%	0.3%	0.2%	90.9%	0.2%	0.2%	1.20021
33	0.1%	0.0%	0.0%	0.0%	0.0%	100.1%	-0.2%	0.99866
34	0.0%	0.0%	0.0%	0.0%	0.0%	100.0%	0.0%	1.00000
35	0.8%	0.5%	0.1%	0.5%	0.1%	98.4%	-0.3%	1.03197
36	0.6%	0.1%	0.4%	0.1%	0.4%	98.4%	-0.0%	1.03252
37	9.0%	0.2%	0.2%	0.2%	0.2%	89.9%	0.3%	1.22461
38	-0.0%	0.0%	-0.0%	0.0%	-0.0%	0.0%	100.0%	1.00000
39	0.1%	0.0%	0.0%	0.0%	0.0%	0.2%	99.6%	1.00736
40	0.6%	0.1%	0.4%	0.1%	0.4%	0.0%	98.4%	1.03322
41	0.8%	0.5%	0.1%	0.5%	0.1%	0.0%	98.2%	1.03757
42	9.0%	0.2%	0.2%	0.2%	0.2%	0.4%	89.8%	1.22715

1-center FNO analysis, $\varepsilon = 0.70000$		$\beta-$**orbitals**
MO	λ	Atoms
1	1.00000	Fe_1
2	1.00000	Fe_1
3	1.00000	Fe_1
4	1.00000	Fe_1
5	1.00000	Fe_1
6	0.99814	Fe_1
7	0.99612	Fe_1
8	0.99522	Fe_1
9	0.99522	Fe_1
10	0.96362	Fe_1
11	0.96362	Fe_1
12	1.00096	F_2
13	1.00000	F_2
14	0.98412	F_2
15	0.97233	F_2
16	0.91076	F_2
17	1.00084	F_3
18	1.00000	F_3
19	0.98479	F_3
20	0.98479	F_3
21	0.91798	F_3
22	1.00096	F_4
23	1.00000	F_4
24	0.98412	F_4
25	0.97233	F_4
26	0.91076	F_4
27	1.00084	F_5
28	1.00000	F_5
29	0.98479	F_5
30	0.98479	F_5
31	0.91798	F_5
32	1.00096	F_6
33	1.00000	F_6
34	0.98412	F_6
35	0.97233	F_6
36	0.91076	F_6
37	1.00000	F_7
38	0.99659	F_7
39	0.98379	F_7
40	0.96955	F_7
41	0.90974	F_7

Localization and atoms expanded by each FNO, $\beta-$**orbitals**								
FNO	Loc_1	Loc_2	Loc_3	Loc_4	Loc_5	Loc_6	Loc_7	n_{eff}
1	100.0%	-0.0%	-0.0%	-0.0%	-0.0%	-0.0%	-0.0%	1.00000
2	100.0%	-0.0%	0.0%	-0.0%	0.0%	-0.0%	0.0%	1.00000
3	100.0%	-0.0%	0.0%	-0.0%	0.0%	-0.0%	0.0%	1.00000
4	100.0%	-0.0%	-0.0%	-0.0%	-0.0%	-0.0%	0.0%	1.00000
5	100.0%	-0.0%	-0.0%	-0.0%	-0.0%	-0.0%	0.0%	1.00000
6	99.8%	0.0%	0.0%	0.0%	0.0%	0.0%	0.0%	1.00372
7	99.6%	0.0%	0.2%	0.0%	0.2%	0.0%	0.0%	1.00781
8	99.5%	0.1%	0.0%	0.1%	0.0%	0.2%	0.2%	1.00962
9	99.5%	0.2%	0.0%	0.2%	0.0%	0.1%	0.1%	1.00962
10	96.4%	0.5%	0.8%	0.5%	0.8%	0.6%	0.6%	1.07666
11	96.4%	0.6%	0.8%	0.6%	0.8%	0.5%	0.5%	1.07666
12	0.1%	100.1%	0.0%	0.2%	0.0%	0.0%	-0.4%	0.99806
13	0.0%	100.0%	0.0%	0.0%	0.0%	0.0%	0.0%	1.00000
14	0.6%	98.4%	0.4%	0.0%	0.4%	0.1%	0.0%	1.03245
15	1.8%	97.2%	0.1%	0.0%	0.1%	0.6%	0.3%	1.05735
16	8.0%	91.1%	0.2%	0.3%	0.2%	0.2%	0.1%	1.19627
17	0.1%	0.0%	100.1%	0.0%	0.2%	0.0%	-0.4%	0.99830
18	0.0%	0.0%	100.0%	0.0%	0.0%	0.0%	-0.0%	1.00000
19	0.5%	0.4%	98.5%	0.4%	0.0%	0.1%	0.1%	1.03106
20	0.5%	0.1%	98.5%	0.1%	0.0%	0.4%	0.4%	1.03106
21	7.3%	0.2%	91.8%	0.2%	0.2%	0.2%	0.2%	1.17925
22	0.1%	0.2%	0.0%	100.1%	0.0%	0.0%	-0.4%	0.99806
23	0.0%	0.0%	0.0%	100.0%	0.0%	0.0%	0.0%	1.00000
24	0.6%	0.0%	0.4%	98.4%	0.4%	0.1%	0.0%	1.03245
25	1.8%	0.0%	0.1%	97.2%	0.1%	0.6%	0.3%	1.05735
26	8.0%	0.3%	0.2%	91.1%	0.2%	0.2%	0.1%	1.19627
27	0.1%	0.0%	0.2%	0.0%	100.1%	0.0%	-0.4%	0.99830
28	0.0%	0.0%	0.0%	0.0%	100.0%	0.0%	-0.0%	1.00000
29	0.5%	0.1%	0.0%	0.1%	98.5%	0.4%	0.4%	1.03105
30	0.5%	0.4%	0.0%	0.4%	98.5%	0.1%	0.1%	1.03105
31	7.3%	0.2%	0.2%	0.2%	91.8%	0.2%	0.2%	1.17925
32	0.1%	0.0%	0.0%	0.0%	0.0%	100.1%	-0.2%	0.99808
33	0.0%	0.0%	0.0%	0.0%	0.0%	100.0%	0.0%	1.00000
34	0.6%	0.1%	0.4%	0.1%	0.4%	98.4%	-0.0%	1.03244
35	1.8%	0.6%	0.1%	0.6%	0.1%	97.2%	-0.3%	1.05731
36	8.0%	0.2%	0.2%	0.2%	0.2%	91.1%	0.2%	1.19627
37	-0.0%	0.0%	-0.0%	0.0%	-0.0%	0.0%	100.0%	1.00000
38	0.1%	0.0%	0.0%	0.0%	0.0%	0.2%	99.7%	1.00685
39	0.6%	0.1%	0.4%	0.1%	0.4%	0.0%	98.4%	1.03315
40	1.7%	0.6%	0.1%	0.6%	0.1%	0.0%	97.0%	1.06337
41	8.0%	0.2%	0.2%	0.2%	0.2%	0.3%	91.0%	1.19893

S1.7 PtO_4^{2+}

1-center FNO analysis, $\varepsilon = 0.90000$		
MO	λ	Atoms
1	1.00000	Pt_1
2	1.00000	Pt_1
3	1.00000	Pt_1
4	1.00000	Pt_1
5	1.00000	Pt_1
6	1.00000	Pt_1
7	1.00000	Pt_1
8	1.00000	Pt_1
9	1.00000	Pt_1
10	1.00000	Pt_1
11	1.00000	Pt_1
12	1.00000	Pt_1
13	1.00000	Pt_1
14	1.00000	Pt_1
15	1.00000	Pt_1
16	0.99999	Pt_1
17	0.99999	Pt_1
18	0.99999	Pt_1
19	0.99998	Pt_1
20	0.99997	Pt_1
21	0.99997	Pt_1
22	0.99997	Pt_1
23	0.99996	Pt_1
24	0.99996	Pt_1
25	0.99996	Pt_1
26	0.99995	Pt_1
27	0.99995	Pt_1
28	0.99995	Pt_1
29	0.99994	Pt_1
30	0.99994	Pt_1
31	0.99701	Pt_1
32	0.99388	Pt_1
33	0.99388	Pt_1
34	0.99387	Pt_1
35	1.00000	O_2
36	0.98486	O_2
37	1.00000	O_3
38	0.98487	O_3
39	1.00000	O_4
40	0.98488	O_4
41	1.00000	O_5
42	0.98487	O_5
1-center FNO analysis, $\varepsilon = 0.90000$		
MO	λ	Atoms
43	0.98974	$Pt_1{-}O_2$
44	0.98207	$Pt_1{-}O_2$
45	0.98206	$Pt_1{-}O_2$
46	0.98975	$Pt_1{-}O_3$
47	0.98206	$Pt_1{-}O_3$
48	0.98206	$Pt_1{-}O_3$
49	0.98975	$Pt_1{-}O_4$
50	0.98206	$Pt_1{-}O_4$
51	0.98206	$Pt_1{-}O_4$
52	0.98975	$Pt_1{-}O_5$
53	0.98207	$Pt_1{-}O_5$
54	0.98206	$Pt_1{-}O_5$

Localization and atoms expanded by each FNO						
FNO	Loc_1	Loc_2	Loc_3	Loc_4	Loc_5	n_{eff}
1	100.0%	0.0%	0.0%	0.0%	0.0%	1.00000
2	100.0%	0.0%	0.0%	0.0%	0.0%	1.00000
3	100.0%	0.0%	0.0%	0.0%	0.0%	1.00000
4	100.0%	0.0%	0.0%	0.0%	0.0%	1.00000
5	100.0%	0.0%	0.0%	0.0%	0.0%	1.00000
6	100.0%	0.0%	0.0%	0.0%	0.0%	1.00000
7	100.0%	0.0%	0.0%	0.0%	0.0%	1.00000
8	100.0%	0.0%	0.0%	0.0%	0.0%	1.00000
9	100.0%	0.0%	0.0%	0.0%	0.0%	1.00000
10	100.0%	0.0%	0.0%	0.0%	0.0%	1.00000
11	100.0%	0.0%	0.0%	0.0%	0.0%	1.00000
12	100.0%	0.0%	0.0%	0.0%	0.0%	1.00000
13	100.0%	0.0%	0.0%	0.0%	0.0%	1.00000
14	100.0%	0.0%	0.0%	0.0%	0.0%	1.00000
15	100.0%	0.0%	0.0%	0.0%	0.0%	1.00001
16	100.0%	0.0%	0.0%	0.0%	0.0%	1.00003
17	100.0%	0.0%	0.0%	0.0%	0.0%	1.00003
18	100.0%	0.0%	0.0%	0.0%	0.0%	1.00003
19	100.0%	0.0%	0.0%	0.0%	0.0%	1.00003
20	100.0%	0.0%	0.0%	0.0%	0.0%	1.00006
21	100.0%	0.0%	0.0%	0.0%	0.0%	1.00006
22	100.0%	0.0%	0.0%	0.0%	0.0%	1.00006
23	100.0%	0.0%	0.0%	0.0%	0.0%	1.00009
24	100.0%	0.0%	0.0%	0.0%	0.0%	1.00009
25	100.0%	0.0%	0.0%	0.0%	0.0%	1.00009
26	100.0%	0.0%	0.0%	0.0%	0.0%	1.00010
27	100.0%	0.0%	0.0%	0.0%	0.0%	1.00010
28	100.0%	0.0%	0.0%	0.0%	0.0%	1.00010
29	100.0%	0.0%	0.0%	0.0%	0.0%	1.00011
30	100.0%	0.0%	0.0%	0.0%	0.0%	1.00011
31	99.7%	0.1%	0.1%	0.1%	0.1%	1.00600
32	99.4%	0.3%	0.1%	0.1%	0.1%	1.01234
33	99.4%	0.1%	0.2%	0.1%	0.3%	1.01235
34	99.4%	0.1%	0.2%	0.3%	0.1%	1.01236
35	0.0%	100.0%	0.0%	0.0%	0.0%	1.00000
36	1.2%	98.5%	0.1%	0.1%	0.1%	1.03081
37	0.0%	0.0%	100.0%	0.0%	0.0%	1.00000
38	1.2%	0.1%	98.5%	0.1%	0.1%	1.03079
39	0.0%	0.0%	0.0%	100.0%	0.0%	1.00000
40	1.2%	0.1%	0.1%	98.5%	0.1%	1.03078
41	0.0%	0.0%	0.0%	0.0%	100.0%	1.00000
42	1.2%	0.1%	0.1%	0.1%	98.5%	1.03079
43	44.5%	54.4%	0.3%	0.3%	0.3%	2.02123
44	40.7%	57.5%	0.7%	0.3%	0.9%	2.01393
45	40.7%	57.5%	0.5%	0.9%	0.3%	2.01391
46	44.5%	0.3%	54.4%	0.3%	0.3%	2.02115
47	40.7%	1.0%	57.5%	0.4%	0.4%	2.01387
48	40.7%	0.2%	57.5%	0.8%	0.8%	2.01387
49	44.5%	0.3%	0.3%	54.5%	0.3%	2.02111
50	40.7%	0.5%	1.0%	57.5%	0.4%	2.01388
51	40.7%	0.7%	0.2%	57.5%	0.8%	2.01389
52	44.5%	0.3%	0.3%	0.3%	54.4%	2.02115
53	40.7%	0.9%	0.3%	0.6%	57.5%	2.01389
54	40.7%	0.3%	0.9%	0.6%	57.5%	2.01387

S2 Generalized Fragment Natural Orbitals (FNO): Eigenvectors

Notice that the numbering of FNOs does not coincide with that in the text.

S2.1 CH_4

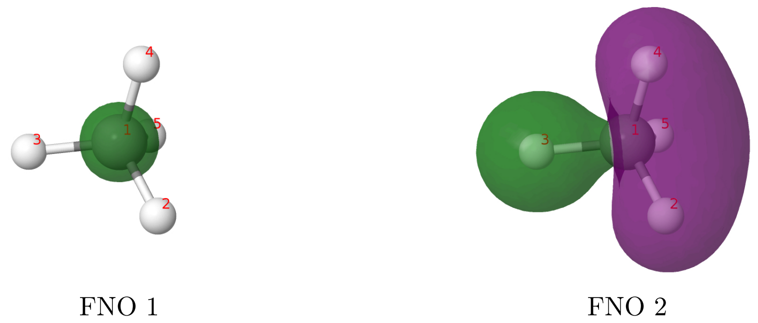

Figure S1: $|\varphi| = 0.05$ a.u. isosurface FNOs of the CH_4 molecule at the restricted Hartree-Fock RHF//def2-QZVPPD level of calculation.

S2.2 SO_4^{2-}

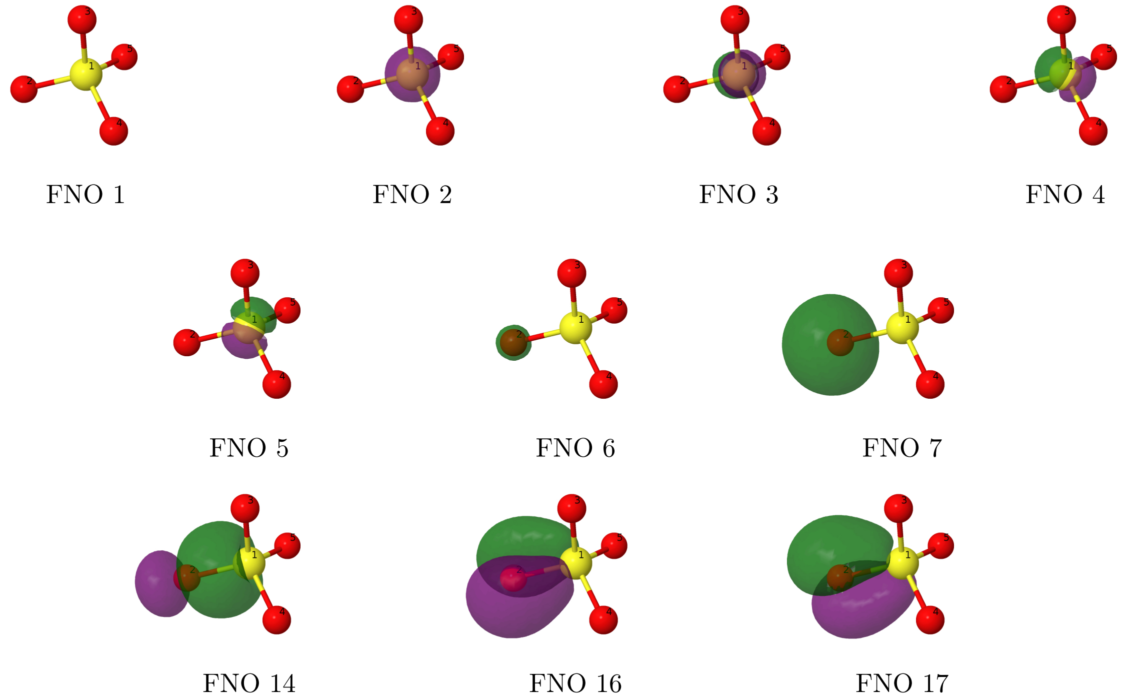

Figure S2: $|\varphi| = 0.05$ a.u. isosurface of the FNOs of the SO_4^{2-} molecule at the B3LYP//def2-QZVPPD level of calculation.

S2.3 N_2H_2

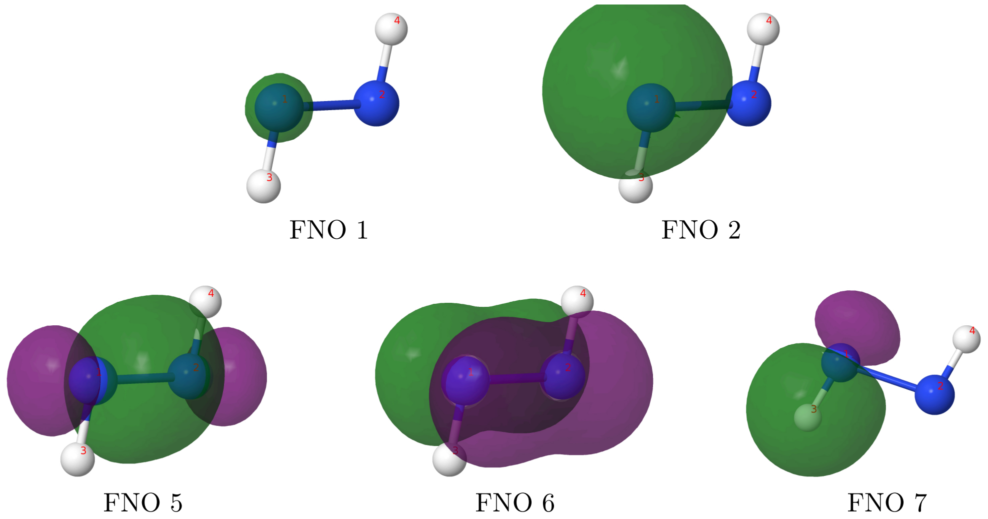

Figure S3: $|\varphi| = 0.05$ a.u. isosurface of the FNOs of the N_2H_2 molecule at the CAS[12,8]//6-311G(d) level of calculation.

S2.4 The cis-butadiene plus ethylene Diels-Alder (DA) reaction

S2.4.1 Reactants

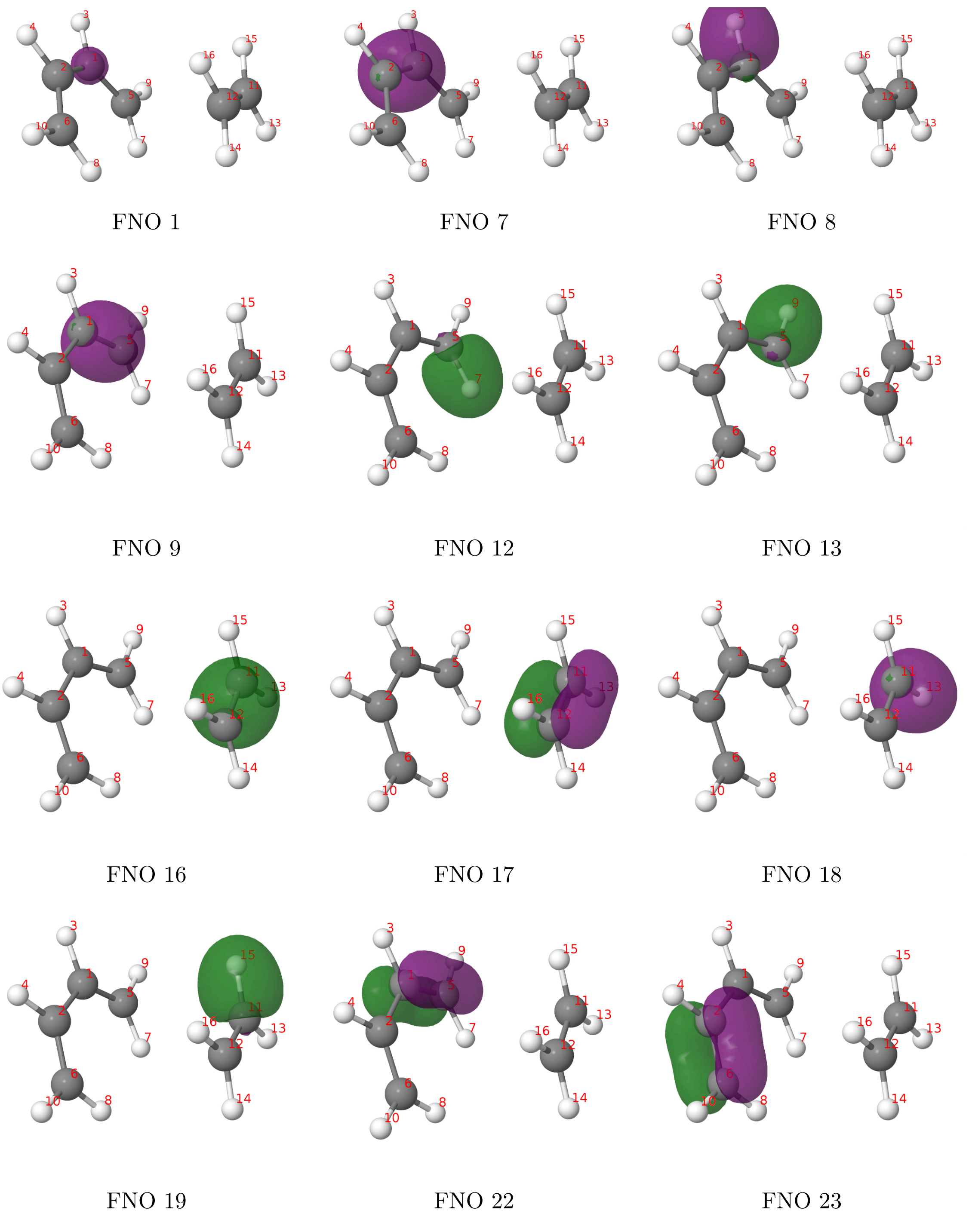

Figure S4: $|\varphi| = 0.1$ a.u. isosurface of the FNOs of the reactants at the B3LYP//aug-cc-pVDZ level of calculation.

S2.4.2 Transition state

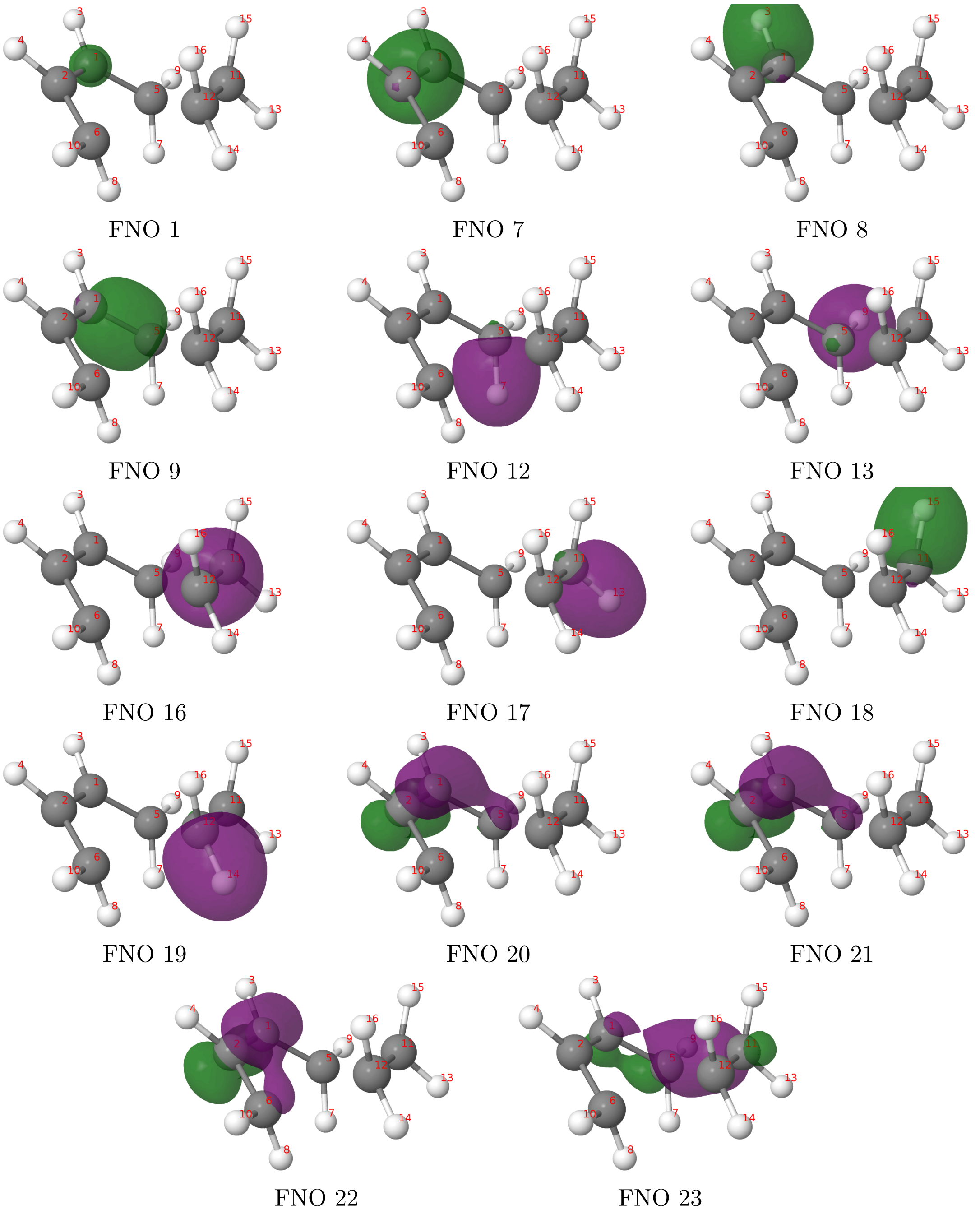

Figure S5: $|\varphi| = 0.1$ a.u. isosurface of the FNOs of the transition state at the B3LYP//aug-cc-pVDZ level of calculation.

S2.4.3 Products

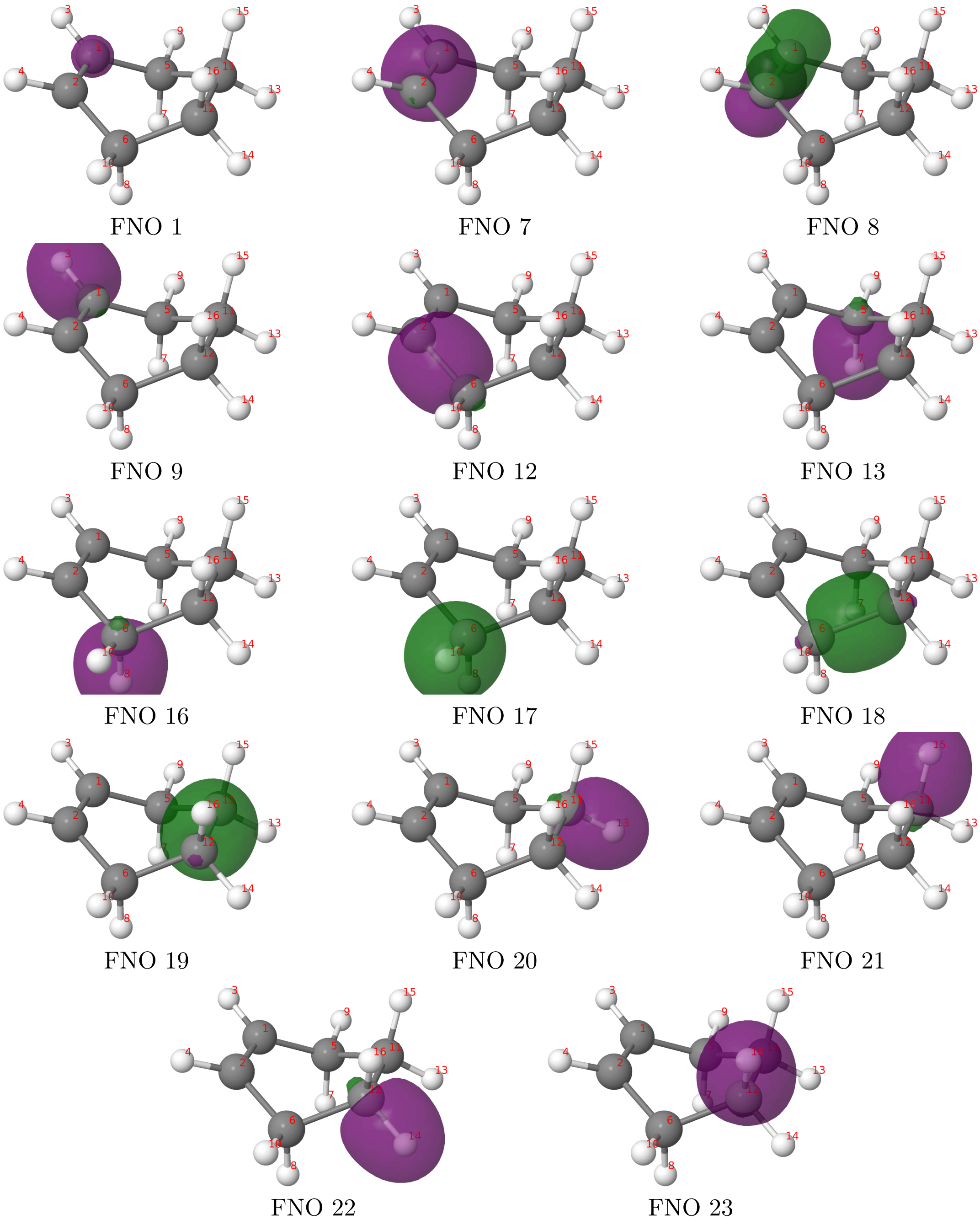

Figure S6: $|\varphi| = 0.1$ a.u. isosurface of the FNOs of the products at the B3LYP//aug-cc-pVDZ level of calculation.

S2.5 The $F^- + CH_3F \rightarrow FCH_3 + F^-$ reaction

S2.5.1 Reactants/Products

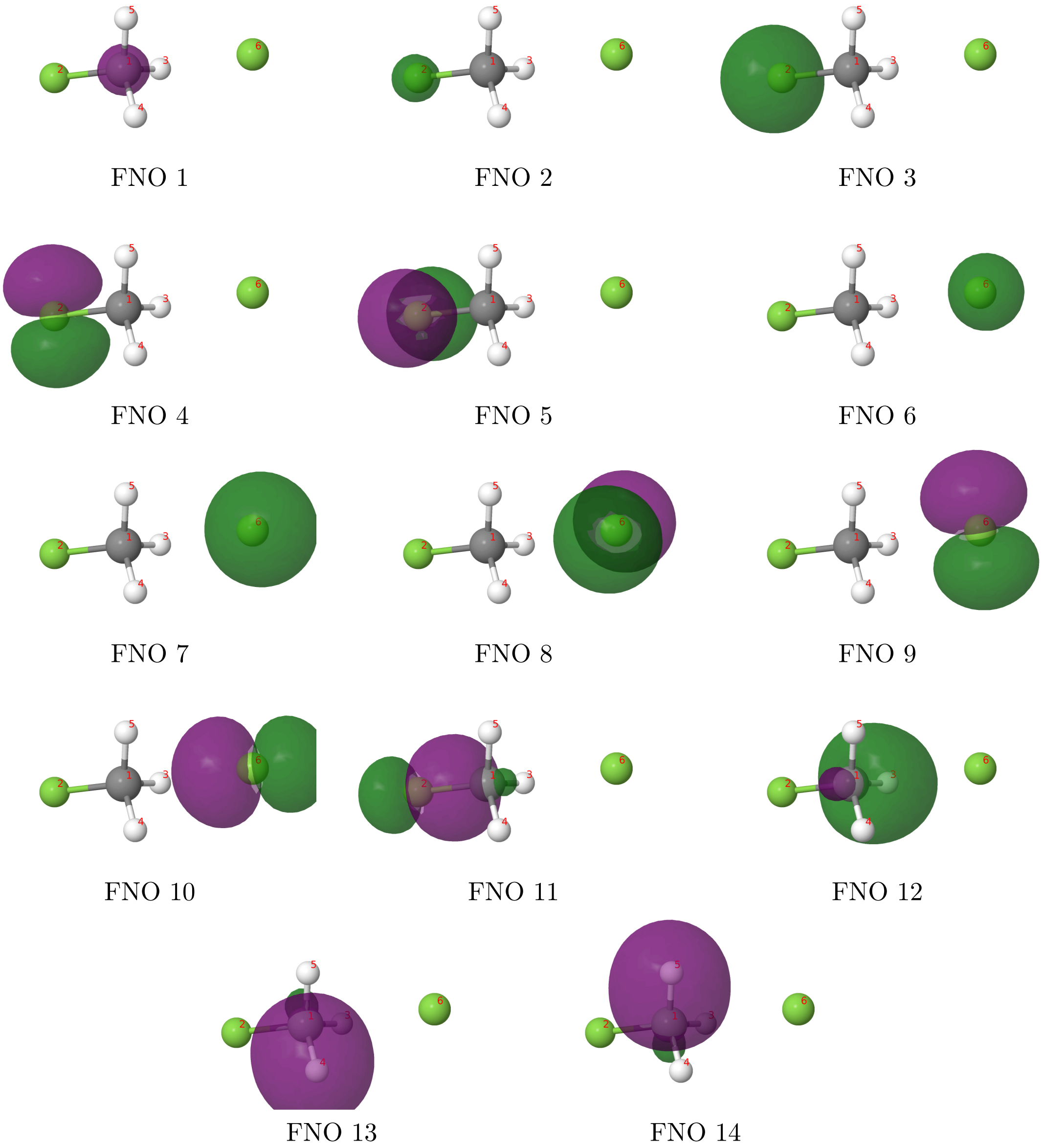

Figure S7: $|\varphi| = 0.05$ a.u. isosurface of the FNOs of the reactants at the B3LYP//aug-cc-pVDZ level of calculation.

S2.5.2 Transition State

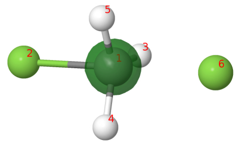
FNO 1

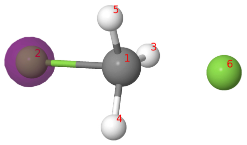
FNO 2

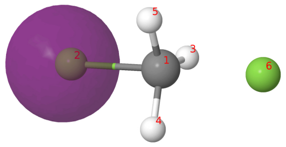
FNO 3

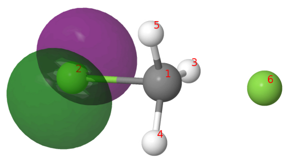
FNO 4

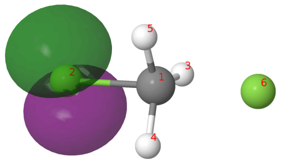
FNO 5

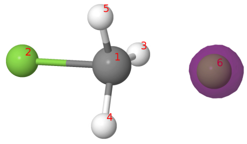
FNO 6

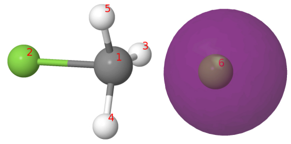
FNO 7

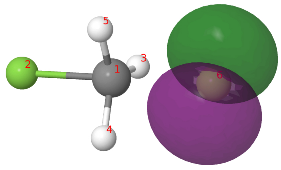
FNO 8

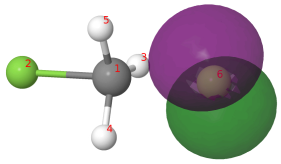
FNO 9

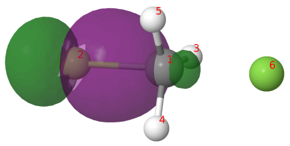
FNO 10

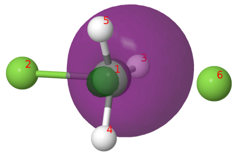
FNO 11

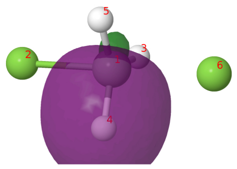
FNO 12

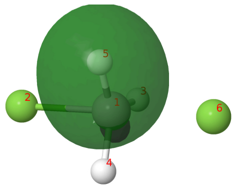
FNO 13

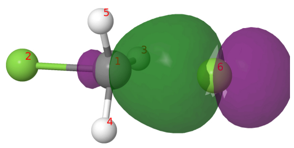
FNO 14

Figure S8: $|\varphi| = 0.05$ a.u. isosurface of the FNOs of the reactants at the B3LYP//aug-cc-pVDZ level of calculation.

S2.6 FeF_6^{3-} complex in Oh (high spin) and D_{4h} (low spin) multielectron states.

S2.6.1 FeF_6^{3-} Oh High spin, ($t_{2g}^3 e_g^2 -^6 A_{1g}$), $\alpha-$orbitals

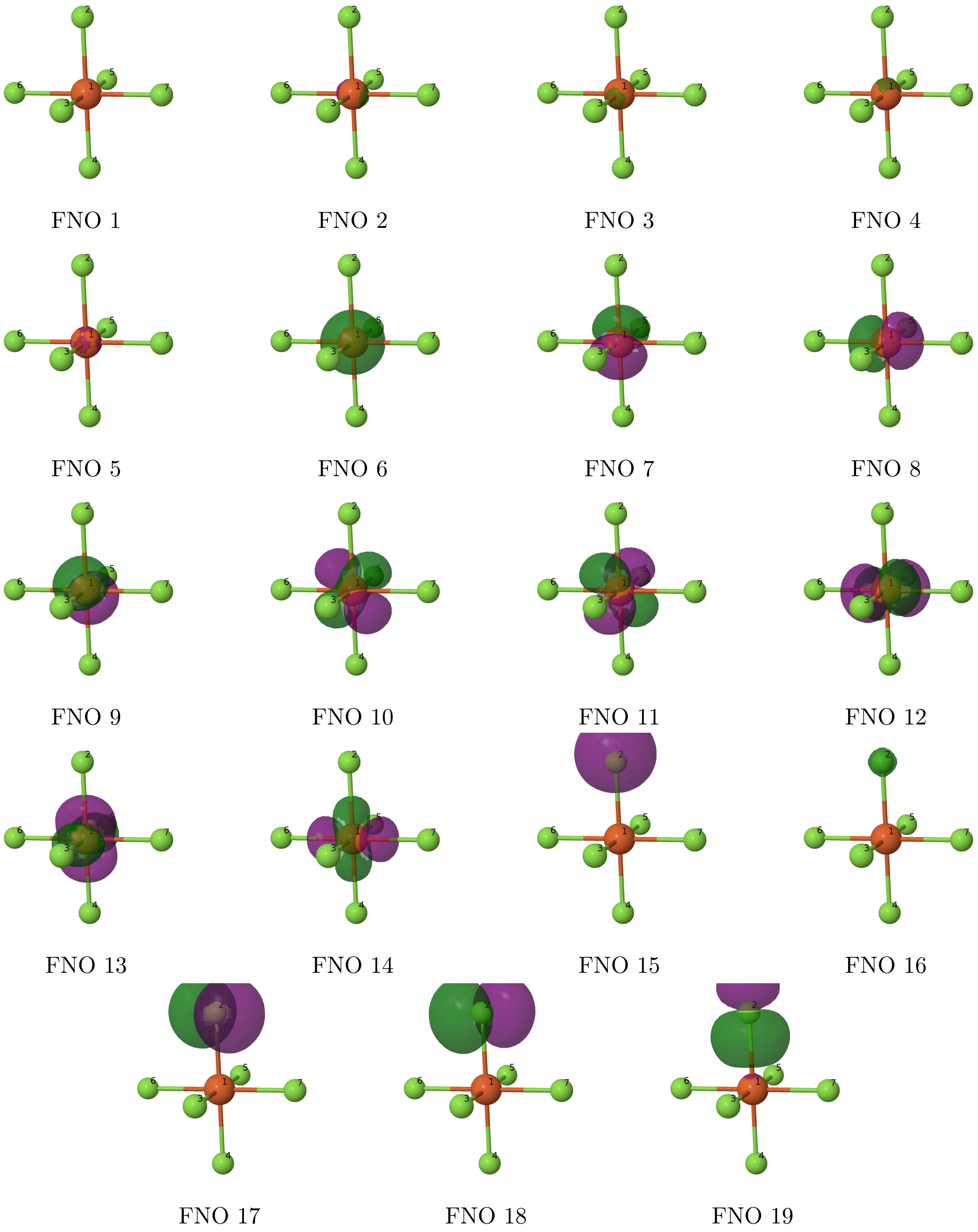

Figure S9: $|\varphi| = 0.05$ a.u. isosurface of the FNOs of the FeF_6^{3-} complex at the unrestricted DFT M06-2X//aug-cc-pVDZ level in the Oh high spin ($t_{2g}^3 e_g^2 -^6 A_{1g}$), $\alpha-$orbitals.

S2.6.2 FeF_6^{3-} Oh High spin, ($t_{2g}^3e_g^2$ $-^6 A_{1g}$), $\beta-$orbitals

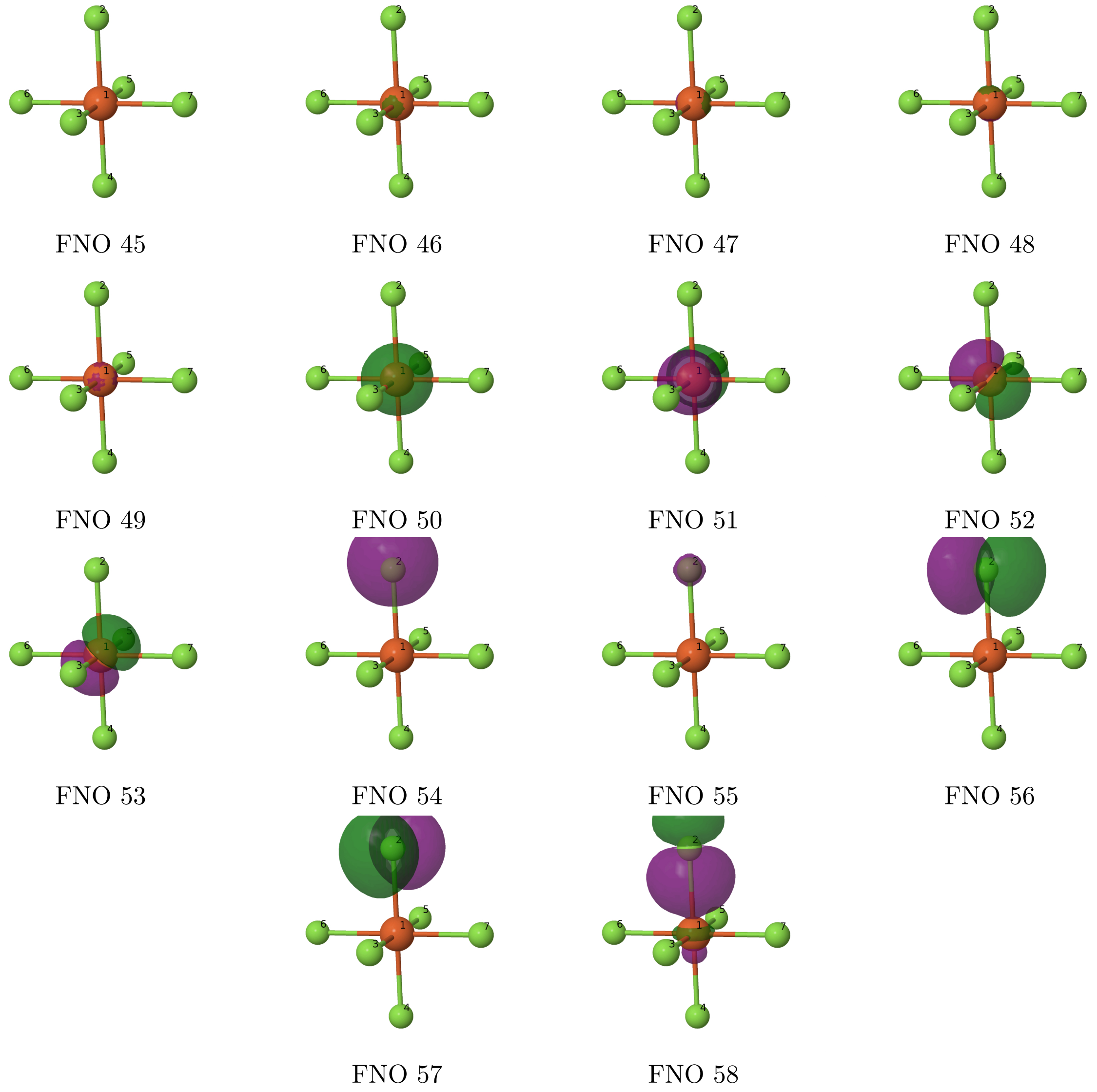

Figure S10: $|\varphi| = 0.05$ a.u. isosurface of the FNOs of the FeF_6^{3-} complex at the unrestricted DFT M06-2X//aug-cc-pVDZ level in the Oh high spin ($t_{2g}^3e_g^2$ $-^6 A_{1g}$), $\beta-$orbitals.

S2.6.3 FeF_6^{3-} D_{4h} Low spin ($e_g^4 b_{2g}^1 -^2 B_{2g}$), α−orbitals

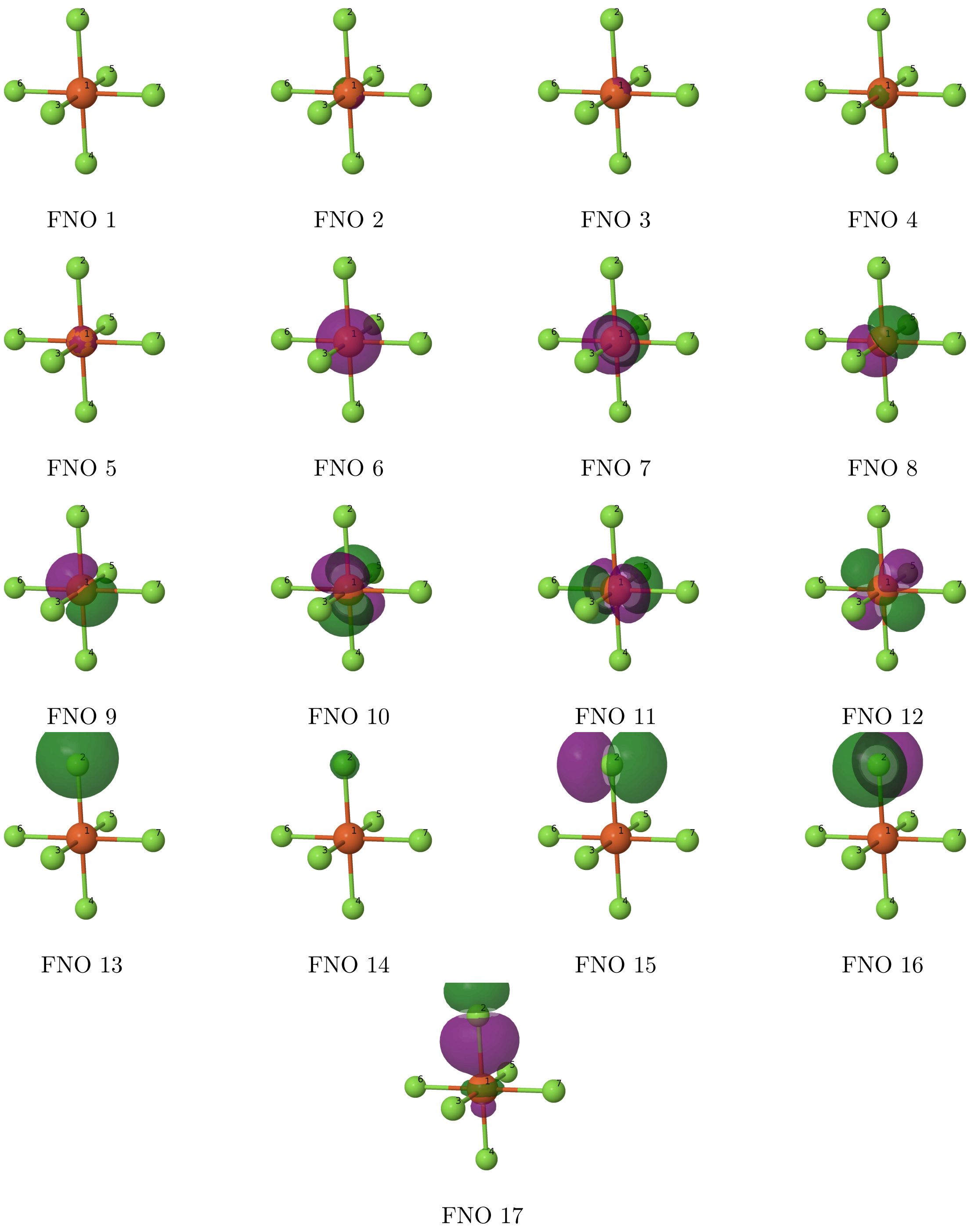

Figure S11: $|\varphi| = 0.05$ a.u. isosurface of the FNOs of the FeF_6^{3-} complex at the unrestricted DFT M06-2X//aug-cc-pVDZ level in the D_{4h} low spin ($e_g^4 b_{2g}^1 -^2 B_{2g}$), α−orbitals.

S2.6.4 FeF_6^{3-} D_{4h} Low spin ($e_g^4 b_{2g}^1 -^2 B_{2g}$) $\beta-$orbitals

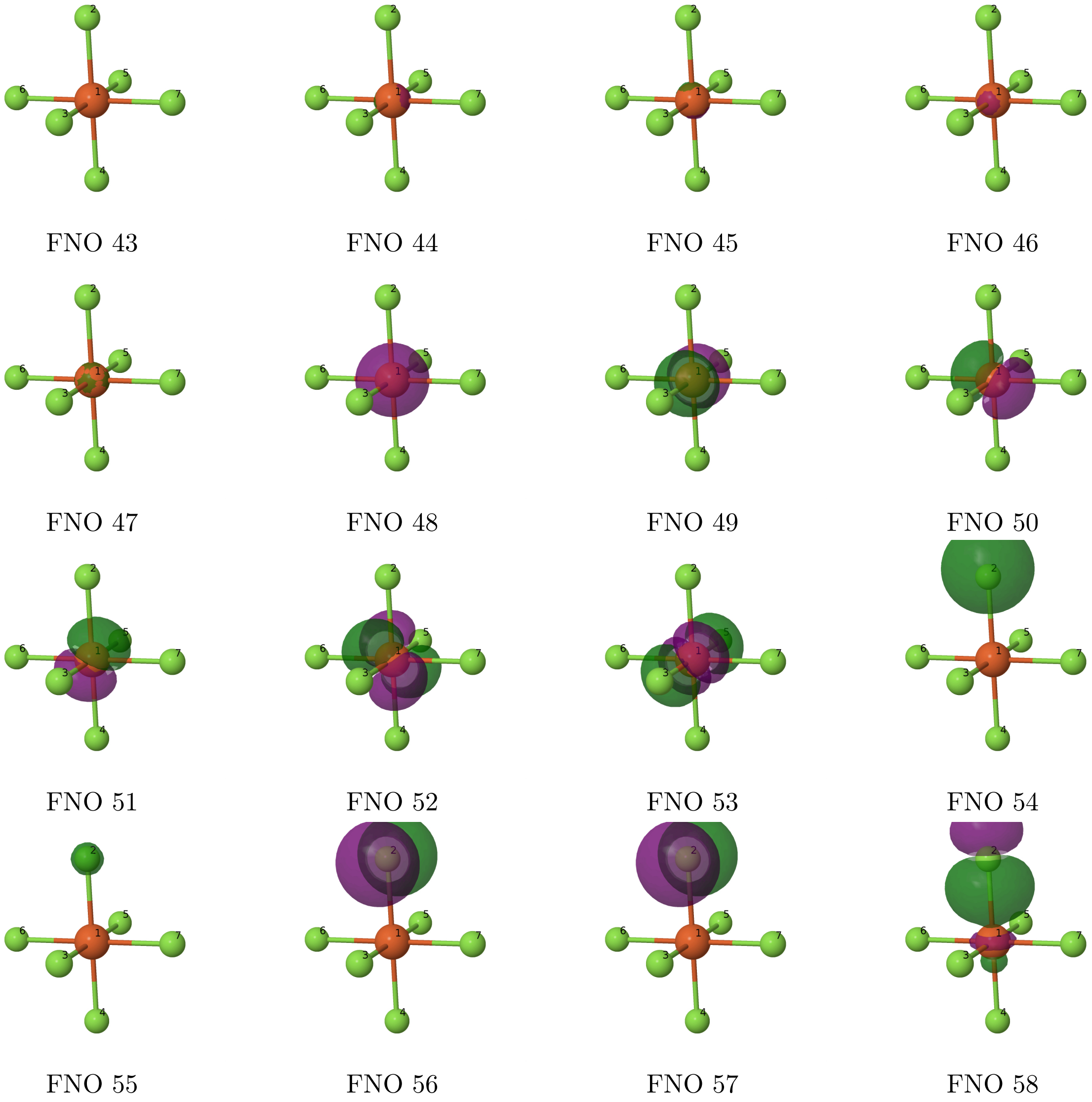

Figure S12: $|\varphi| = 0.05$ a.u. isosurface of the FNOs of the FeF_6^{3-}complex at the unrestricted DFT M06-2X//aug-cc-pVDZ level in the D_{4h} low spin ($e_g^4 b_{2g}^1 -^2 B_{2g}$), $\beta-$orbitals.

S2.7 PtO_4^{2+}

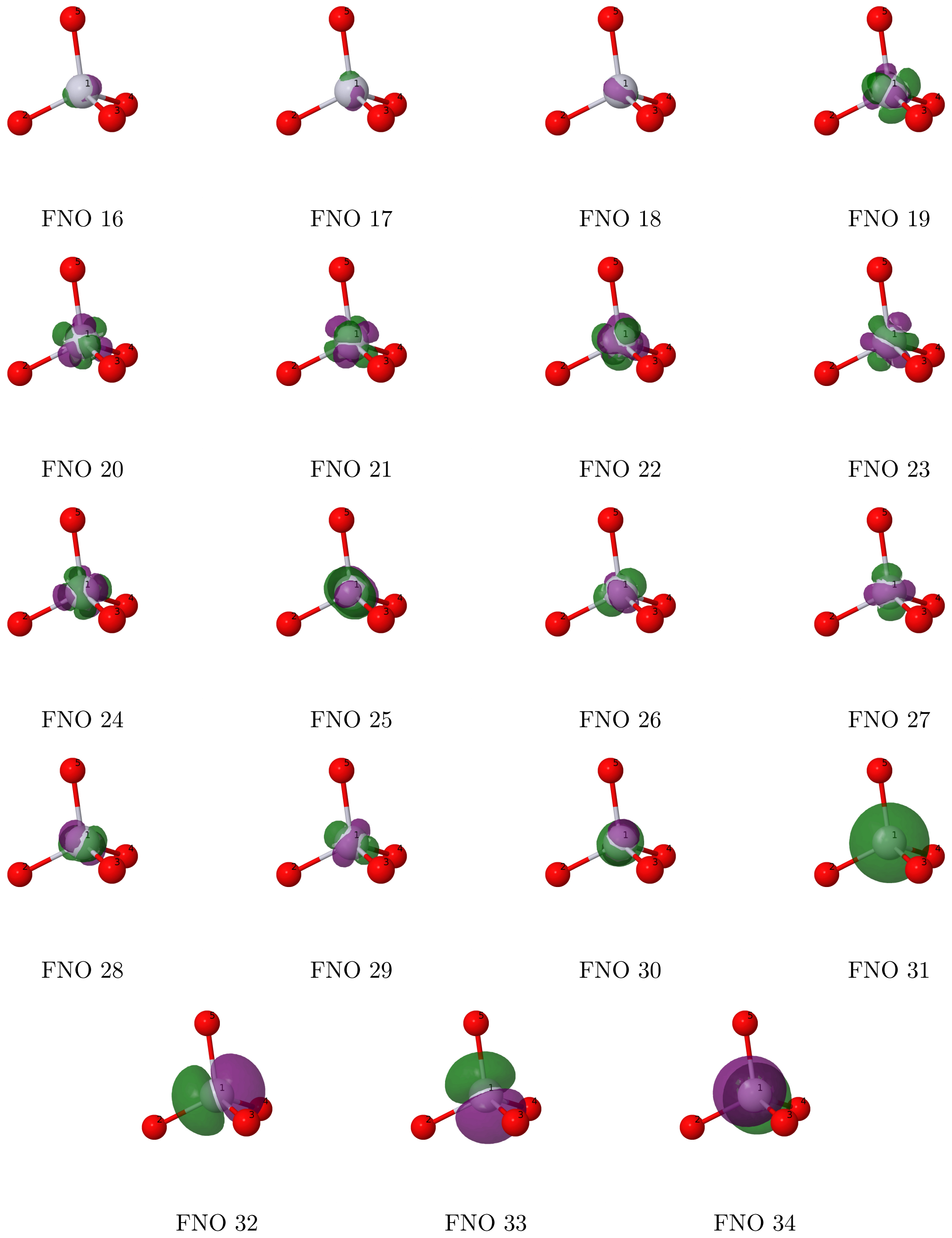

Figure S13: $|\varphi| = 0.05$ a.u. isosurface of the FNOs of the PtO_4^{2+} complex as obtained from heat bath CI (HCI) calculations using ADZP (Pt) def2-QZVPPD (O) basis sets. These FNOs correspond to the external shell of the Pt atom.

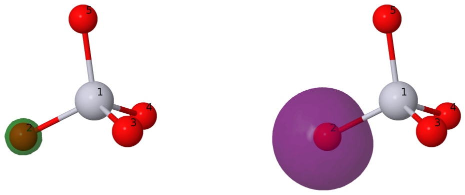

FNO 35 FNO 36

Figure S14: $|\varphi| = 0.05$ a.u. isosurface of the FNOs of the PtO_4^{2+} complex as obtained from heat bath CI (HCI) calculations using ADZP (Pt) def2-QZVPPD (O) basis sets. These FNOs are mainly localized on the oxygen atom.

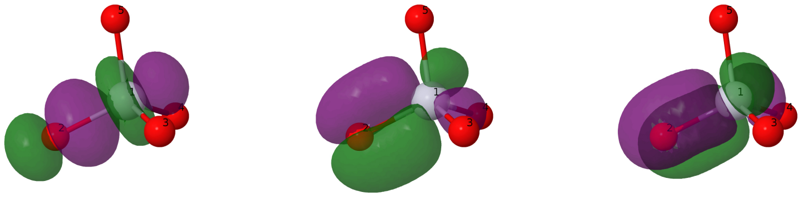

FNO 43 FNO 44 FNO 45

Figure S15: $|\varphi| = 0.05$ a.u. isosurface of the FNOs of the PtO_4^{2+} complex as obtained from heat batch CI (HCI) calculations using ADZP (Pt) def2-QZVPPD (O) basis sets. These FNOs correspond to the σ (left) and π (center and right) Pt-O bonds.

S3 Cartesian coordinates

S3.1 CH_4

```
C1  0.00000000  0.00000000  0.00000000
H2  1.20000000  1.20000000  1.20000000
H3 -1.20000000 -1.20000000  1.20000000
H4 -1.20000000  1.20000000 -1.20000000
H5  1.20000000 -1.20000000 -1.20000000
```

S3.2 SO_4^{2-}

```
S1  0.00000000  0.00000000  0.00000000
O2 -1.63990201 -1.63990201  1.63990201
O3  1.63990201  1.63990201  1.63990201
O4  1.63990201 -1.63990201 -1.63990201
O5 -1.63990201  1.63990201 -1.63990201
```

S3.3 N_2H_2

```
N1 -1.51178079  0.00000000  0.00000000
N2  1.51178079  0.00000000  0.00000000
H3 -1.78851470 -1.89098960  0.00000000
H4  1.78851470  1.89098960  0.00000000
```

S3.4 The cis-butadiene plus ethylene Diels-Alder (DA) reaction

S3.4.1 Reactants

```
C1  -2.74331542  0.56332736 -1.38838178
C2  -2.74350439  0.56313839  1.38857076
H3  -3.76225574  2.13274490 -2.26559265
H4  -3.76282266  2.13198901  2.26578162
C5  -1.62459755 -1.12665472 -2.91981584
C6  -1.62440858 -1.12703266  2.91962686
```

```
H7  -0.57731133 -2.74728384 -2.19964121
H8  -0.57674441 -2.74709487  2.19945224
H9  -1.73854803 -0.93484751 -4.96676717
H10 -1.73835906 -0.93560340  4.96676717
C11  4.17667268  0.59734243 -1.26158116
C12  4.17686165  0.59809832  1.26177013
H13  4.86906833 -1.01440498 -2.34571704
H14  4.86944628 -1.01289320  2.34666190
H15  3.48106449  2.20549936 -2.34458320
H16  3.48125347  2.20682217  2.34382731
```

S3.4.2 Transition state

```
C1  -2.42508554  0.62115298 -1.29843082
C2  -2.42508554  0.62115298  1.29843082
H3  -3.50090662  2.06320298 -2.30962327
H4  -3.50090662  2.06320298  2.30962327
C5  -0.62644421 -0.87021888 -2.66167925
C6  -0.62625524 -0.87021888  2.66149027
H7  -0.33882789 -2.81984932 -2.05262052
H8  -0.33863892 -2.81984932  2.05243154
H9  -0.58978352 -0.66593949 -4.71637846
H10 -0.58959455 -0.66612846  4.71618949
C11  2.84214809  0.29101782 -1.37496473
C12  2.84195912  0.29120680  1.37496473
H13  3.98467651 -1.15443369 -2.30489895
H14  3.98467651 -1.15405574  2.30508793
H15  2.93285495  2.12480805 -2.31151300
H16  2.93266597  2.12499703  2.31132402
```

S3.4.3 Products

```
C1  -2.34571704  0.63362517 -1.26687239
C2  -2.34590601  0.63362517  1.26687239
H3  -3.83425431  1.56280351 -2.35176416
H4  -3.83387636  1.56280351  2.35157519
C5  -0.20541323 -0.68615956 -2.60801102
C6  -0.20560220 -0.68615956  2.60801102
H7  -0.42632221 -2.75049637 -2.39314916
H8  -0.42632221 -2.75030740  2.39314916
H9  -0.23186940 -0.30519077 -4.64740346
H10 -0.23130248 -0.30481282  4.64740346
C11  2.36782683  0.10903720 -1.46812823
C12  2.36801581  0.10884822  1.46812823
H13  3.85277362 -1.14706376 -2.19831840
H14  3.85315157 -1.14725273  2.19812943
H15  2.81720371  2.01520394 -2.15806723
H16  2.81758165  2.01501497  2.15825621
```

S3.5 The $F^- + CH_3F \rightarrow FCH_3 + F^-$ reaction

S3.5.1 Reactants/Products

```
C1 -0.75552138  0.00012666 -0.00021348
F2 -3.46379758 -0.00011228  0.00017787
H3 -0.09624816  1.83210960 -0.67184363
H4 -0.09571606 -1.49729274 -1.25095715
H5 -0.09505693 -0.33414950  1.92176713
F6  3.99936974 -0.00004631  0.00007930
```

S3.5.2 Transition state

```
C1  0.00005744  0.00037643  0.00000000
F2  0.00005744 -0.00013937  3.40230072
H3 -2.03155810  0.21207733  0.00000000
```

H4	0.83093233	-1.86534426	0.00000000
H5	1.19924724	1.65351694	0.00000000
F6	0.00005744	-0.00013937	-3.40230072

S3.6 FeF_6^{3-} complex in Oh (high spin) and D_{4h} (low spin) multielectron states.

S3.6.1 FeF_6^{3-} Oh

Fe1	0.00000000	0.00000000	0.00000000
F2	0.00000000	0.00000000	3.77994079
F3	0.00000000	3.77994079	0.00000000
F4	0.00000000	0.00000000	-3.77994079
F5	0.00000000	-3.77994079	0.00000000
F6	3.77994079	0.00000000	0.00000000
F7	-3.77994079	0.00000000	0.00000000

S3.6.2 FeF_6^{3-} D_{4h}

Fe1	0.00000000	0.00000000	0.00000000
F2	0.00000000	3.60197779	0.00000000
F3	0.00000000	0.00000000	-3.72221158
F4	0.00000000	-3.60197779	0.00000000
F5	0.00000000	0.00000000	3.72221158
F6	3.60197779	0.00000000	0.00000000
F7	-3.60197779	0.00000000	0.00000000

S3.7 PtO_4^{2+}

Pt1	0.00000000	0.00000000	0.00000000
O2	-1.86844345	-1.86844345	1.86844345
O3	1.86844345	1.86844345	1.86844345
O4	1.86844345	-1.86844345	-1.86844345
O5	-1.86844345	1.86844345	-1.86844345
